# Depletion of oxysterol-binding proteins by OSW-1 triggers RIP1/RIP3-independent necroptosis and sensitization to cancer immunotherapy

**DOI:** 10.1038/s41418-025-01521-8

**Published:** 2025-05-06

**Authors:** Xinyan Lu, Dongshi Chen, Min Wang, Xiangping Song, Kaylee Ermine, Suisui Hao, Anupma Jha, Yixian Huang, Ying Kang, Haibo Qiu, Heinz-Josef Lenz, Song Li, Zhendong Jin, Jian Yu, Lin Zhang

**Affiliations:** 1 Department of Medicine, Keck School of Medicine of University of Southern California (USC), Los Angeles, CA USA; 2 Norris Comprehensive Cancer Center, Keck School of Medicine of USC, Los Angeles, CA USA; 3 Department of Pharmacology and Chemical Biology, University of Pittsburgh School of Medicine, Pittsburgh, PA USA; 4 UPMC Hillman Cancer Center, University of Pittsburgh School of Medicine, Pittsburgh, PA USA; 5 Department of Pharmaceutical Sciences, Center for Pharmacogenetics, School of Pharmacy, University of Pittsburgh, Pittsburgh, PA USA; 6 Department of Pharmaceutical Sciences and Experimental Therapeutics, College of Pharmacy, University of Iowa, Iowa City, IA USA

**Keywords:** Drug development, Cancer microenvironment

## Abstract

Oxysterol-binding proteins (OSBPs), lipid transfer proteins functioning at intracellular membrane contact sites, are recently found to be dysregulated in cancer and promote cancer cell survival. However, their role as potential targets in cancer therapy remains largely unexplored. In this study, we found OSW-1, a natural compound and OSBP inhibitor, potently and selectively kills colon cancer cells by activating a previously unknown necroptosis pathway that is independent of receptor-interacting protein 1 (RIP1) and RIP3. OSW-1 stabilizes p53 and degrades OSBPs to promote endoplasmic reticulum (ER) stress and glycogen synthase kinase 3β (GSK3β)/Tip60-mediated p53 acetylation at Lysine 120, which selectively induces its target PUMA. PUMA-mediated mitochondrial calcium influx activates calcium/calmodulin-dependent protein kinase IIδ (CamKIIδ) to promote mixed lineage kinase domain-like (MLKL) phosphorylation and necroptotic cell death. Furthermore, OSW-1-induced necroptosis is highly immunogenic and sensitizes syngeneic colorectal tumors to anti-PD-1 immunotherapy. Together, our results identified a novel RIP1/RIP3-independent necroptosis pathway underlying the extremely potent anticancer activity of OSW-1, which can be harnessed to develop new anticancer therapies by selectively stimulating antitumor immunity.

## Introduction

Induction of programmed cell death (PCD) is a critical mechanism of anticancer therapy. In addition to apoptosis, recent studies identified necroptosis as another key form of PCD in human physiology and pathology [1]. Necroptosis serves as a major cell death mechanism in response to severe stress and blocked apoptosis [2]. Cells undergoing necroptosis have morphological features including loss of plasma membrane integrity, translucent cytoplasm, and organelle swelling [3]. Necroptosis is characterized by the controlled release of intracellular contents such as the nuclear protein high mobility group box 1 (HMGB1) and ATP [4]. At the molecular level, induction of TNF-α-mediated necroptosis is governed by serine-threonine kinases receptor-interacting protein 1 (RIP1) and RIP3, and mixed lineage kinase domain-like (MLKL) pseudo kinase [5–7]. RIP1 and RIP3 form a functional amyloid signaling complex known as the necrosome [8], which recruits and phosphorylates MLKL, leading to plasma membrane permeabilization and eventual cell death [9]. While both RIP1 and RIP3 are not always required for necroptosis, either one seems to be indispensable for promoting MLKL phosphorylation and subsequent cell death [2, 3, 7, 10].

Necroptosis plays a critical role in tumor progression and anticancer therapy. Mutations or downregulation of *RIP1*, *RIP3*, and *MLKL* were found in various types of cancer [11–13]. Many anticancer agents can promote necroptosis in addition to apoptosis [14]. Compared to apoptosis, necroptosis often elicits a more robust immune response [4], a defense mechanism against tumor-causing mutations and viruses [15]. Targeting defective necroptosis in cancer cells to harness its immunogenic effect has not been extensively explored [16]. We recently identified a necroptosis pathway mediated by p53 upregulated modulator of apoptosis (PUMA) [17], a p53 target and a BH3-only Bcl-2 family protein [18], in the killing of a subset of colorectal cancer (CRC) cells expressing RIP3 [19]. However, this pathway often cannot be engaged in CRC cells due to frequent loss of *RIP3* expression. This prompted us to search for agents that can restore necroptosis in RIP3-deficient CRC cells.

A natural compound, OSW-1 (Supplementary Fig. 1A), is one of the most potent anticancer agents. OSW-1 is a steroidal saponin found in the bulbs of *Ornithogalum saudersiae* [20, 21]. It exhibited extremely potent cytotoxic activity of pico- to low-nanomolar half maximal inhibitory concentration (IC_50_) against cultured human cancer cells but was much less toxic (40-150-fold higher IC_50_) to non-transformed cells [22]. Despite its remarkable potency and selectivity in vitro, further development of OSW-1 has been hampered by dose-limiting toxicity in immune-deficient tumor models that is not well understood. Recent studies showed that OSW-1 treatment depletes oxysterol binding protein (OSBP) and OSBP-related protein 4 (ORP4) [23], members of ORP family of intracellular lipid binding/transfer proteins that are involved in inter-organelle lipid transfer over membrane contact sites [24]. Emerging evidence suggests that ORP family members are dysregulated in cancer and required for cancer cell survival [25–27]. However, their role as cancer therapeutic targets is not well understood.

In this study, we explored the therapeutic potential of targeting OSBPs using OSW-1 and found it is a potent inducer of necroptosis in CRC cells. The anticancer activity of OSW-1 is mediated by p53/PUMA-dependent necroptosis bypassing RIP1 and RIP3. Importantly, the antitumor activity of OSW-1 in vivo is largely mediated by necroptosis-triggered antitumor immunity, which strongly sensitizes CRC to anti-PD-1 immunotherapy.

## Results

### Identifying OSW-1 as a novel inducer of RIP1/RIP3-independent necroptosis

**Fig. 1:**
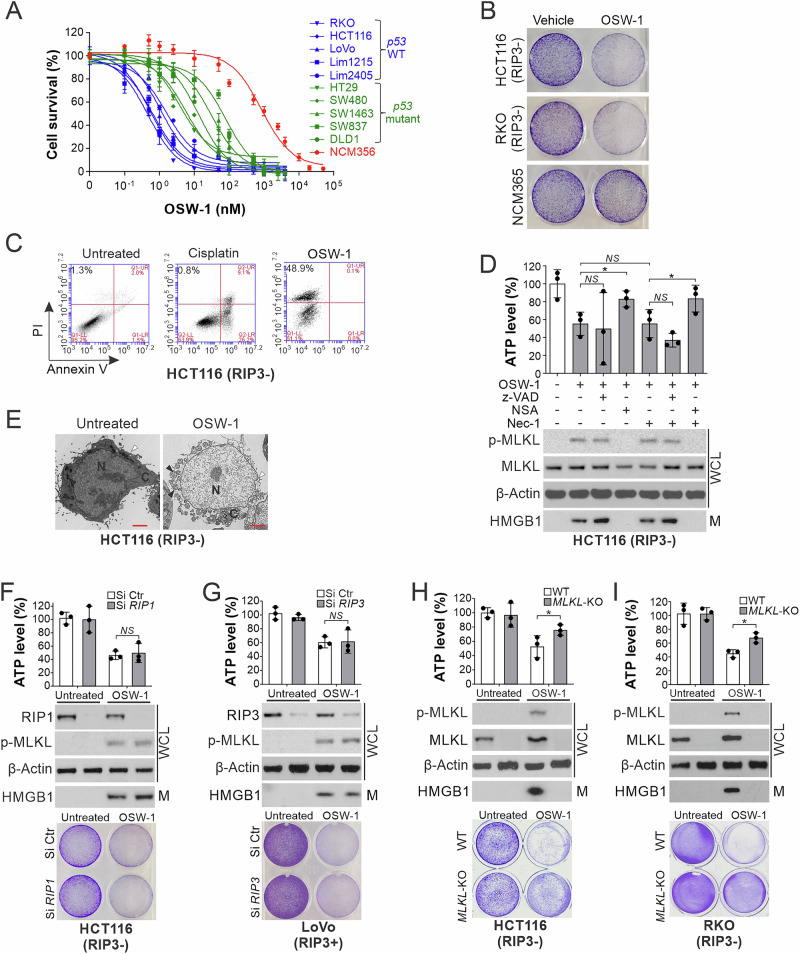
OSW-1 induces RIP1/RIP3-independent necroptosis in CRC cells. **A** MTS analysis of indicated CRC cell lines and NCM356 normal colonic epithelial cells treated with OSW-1 at indicated concentrations for 48 h. **B** Crystal violet staining of RIP3- HCT116 and RKO cells and NCM356 cells treated with OSW-1 (0.5 nM) for 24 h. **C** HCT116 cells treated with OSW-1 as in (**B**) were analyzed by Annexin V/PI staining followed by flow cytometry. Cells treated with cisplatin (50 μM) were used as a positive control for apoptosis. **D** HCT116 cells were treated for 24 h with OSW-1 (0.5 nM) alone or in combination with the RIP1 inhibitor Necrostatin-1 (Nec-1; 20 μM), the MLKL inhibitor Necrosulfonamide (NSA; 2 μM), and/or the pan-caspase inhibitor z-VAD-fmk (z-VAD; 10 μM). *Upper*, ATP levels in treated cells; *lower*, western blotting of total and phosphorylated MLKL (p-MLKL; S358) in whole cell lysates (WCL) and HMGB1 in 20-μl cell culture medium (M). **E** Transmission electron microscopy (TEM) of HCT116 cells with or without OSW-1 treatment as in (**B**). White arrowheads indicate plasma membrane, and black arrowheads denote mitochondria. Scale bars: 2 μm. **F** HCT116 cells transfected with control (Ctr) or *RIP1* siRNA were treated with OSW-1 as in (**B**). Necroptosis was analyzed by measuring ATP levels (*upper*), Western blotting of indicated proteins (*middle*), and crystal violet staining (*lower*). **G** LoVo cells transfected with Ctr or *RIP3* siRNA were treated with OSW-1 and analyzed for necroptosis as in (**F**). **H**, **I** WT and *MLKL*-KO **H** HCT116 and **I** RKO cells were treated with OSW-1 and analyzed for necroptosis as in (**F**). Quantitative results in (**A**, **D**, **F**–**I**) were expressed as means ± s.d. of three independent experiments. *NS*, *P* > 0.05; *; *P* < 0.05.

Our recent study revealed that frequent loss of *RIP3* expression compromises chemotherapy-induced necroptosis in CRC cells [19], and prompted us to search for agents that can bypass RIP3 to induce necroptosis. Upon analyzing over 50 anticancer agents, we identified a plant-derived compound OSW-1 (Supplementary Fig. 1A), which induces necroptosis in CRC cells with or without RIP3 expression (Supplementary Fig. 1B, C). OSW-1 is highly selective to CRC cell lines (IC_50_ 0.46–24.5 nM), compared to normal colonic epithelial NCM356 cells (IC_50_ 0.85 mM; over 1000-fold) (Fig. 1A, B). In RIP3-silenced (RIP3-) HCT116 cells, OSW-1-induced cell death showed little nuclear fragmentation or caspase activation (Supplementary Fig. 1D, E) and was not blocked by inhibitors of apoptosis, autophagy, ferroptosis, and paraptosis (Supplementary Fig. 1F–I). Several hallmarks of necroptosis were observed [19], including propidium iodide (PI)+/annexin V- staining, ATP depletion, extracellular release of nuclear HMGB1 and cytoplasmic LDH, and MLKL S358 phosphorylation (p-MLKL) (Fig. 1C, D and Supplementary Fig. 2A). Transmission electron microscopy (TEM) identified morphological features of necroptosis, including plasma membrane rupture but intact nuclear membrane, translucent cytosol, and mitochondrial swelling (Fig. 1E). OSW-1-induced necroptosis was abrogated by inhibition or knockdown (KD) of MLKL, but not RIP1 or RIP3, in CRC cell lines with or without RIP3 expression, including RIP3- HCT116, RKO, and Lim2405 cells, and RIP3+ LoVo and Lim1215 cells (Fig. 1D–G and Supplementary Fig. 2B–G). Furthermore, knockout (KO) of *MLKL* by CRISPR suppressed OSW-1-induced necroptosis in HCT116 and RKO cells (Fig. 1H, I). To our knowledge, OSW-1 is the first RIP1/RIP3-independent inducer of p-MLKL and necroptosis identified.

### OSW-1-induced necroptosis is mediated by p53-dependent transcriptional upregulation of *PUMA*

**Fig. 2:**
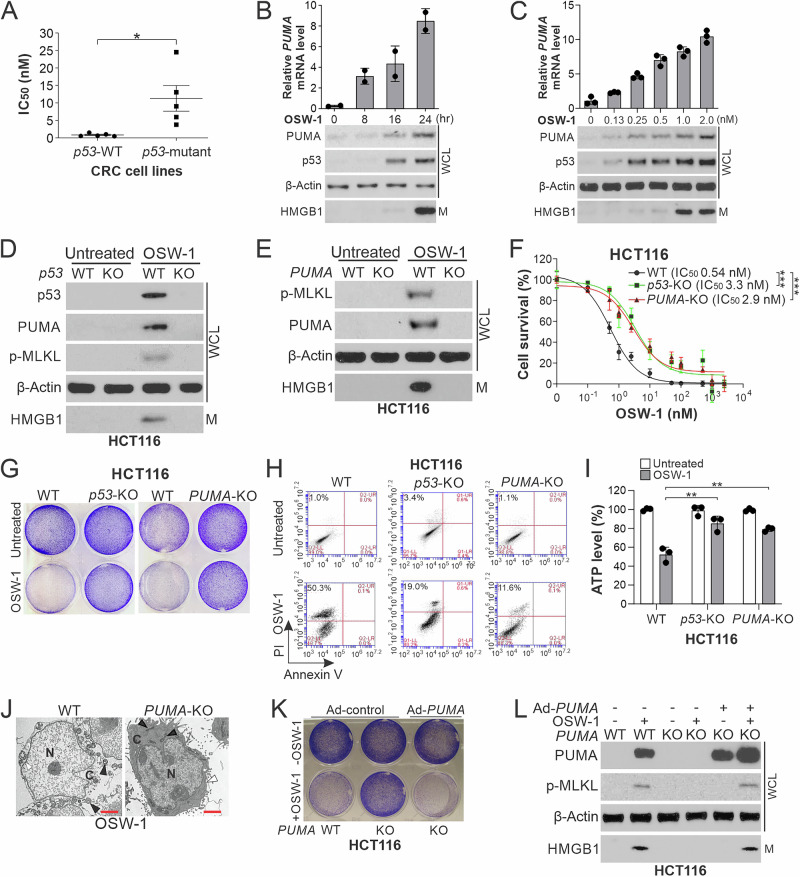
OSW-1-induced necroptosis requires p53-mediated transcriptional upregulation of *PUMA.* **A** Comparison of OSW-1 IC_50_ of *p53*-wildtype (WT) and *p53*-mutant CRC cell lines analyzed in Fig. 1A. **B**, **C** HCT116 cells were treated with (**B**) OSW-1 (0.5 nM) for indicated time, or (**C**) OSW-1 at indicated concentrations for 24 h. *Upper*, real time RT-PCR analysis of *PUMA* mRNA expression; *lower*, western blotting of indicated proteins in whole cell lysates (WCL) and HMGB1 in 20-μl cell culture medium (M). **D**, **E** Western blotting of indicated proteins in **D** WT and *p53*-knockout (*p53*-KO), **E** WT and *PUMA*-KO HCT116 cells treated with OSW-1 (0.5 nM) for 24 h. **F** MTS analysis of WT, *p53*-KO, and *PUMA*-KO HCT116 cells treated with OSW-1 at indicated concentrations for 48 h. **G-I** WT, *p53*-KO, and *PUMA*-KO HCT116 cells were treated with OSW-1 as in (**D**) and analyzed by (**G**) crystal violet staining of viable cells, **H** annexin-V/PI staining followed by flow cytometry, **I** measuring ATP levels, and **J** TEM. In (**J**), representative TEM images are shown. White arrowheads indicate plasma membrane, and black arrowheads denote mitochondria. Scale bars, 2 μm. **K**, **L** WT and *PUMA*-KO HCT116 cells with or without infection with an adenovirus expressing PUMA (Ad-*PUMA*) or control BH3-deleted PUMA (Ad-control) were treated with OSW-1 as in (**D**). **K** Crystal violet staining of viable cells. **L** Western blotting of indicated proteins. Quantitative results in (**B**, **C**, **F**, **I**) were expressed as means ± s.d. of two or three independent experiments. *, *P* < 0.05; **, *P* < 0.01; ***, *P* < 0.001.

We identified a correlation of OSW-1 sensitivity with the status of *p53* in CRC cells, with *p53*-wildtype (WT) CRC cell lines showing significantly lower IC_50_ (Figs. 1A and 2A). In sync with HMGB1 release, OSW-1 treatment upregulated p53 and PUMA expression in *p53*-WT HCT116 cells in a time- and dose-dependent manner (Fig. 2B, C). OSW-1 did not affect the levels of other Bcl-2 family proteins, including proapoptotic Noxa, Bid, Bim, Bax, and Bak, and antiapoptotic Bcl-2, Bcl-X_L_, and Mcl-1 (Supplementary Fig. 3A). The induction of PUMA by OSW-1 is mediated by transcriptional activation by WT p53, but not other transcriptional factors such as FOXO3a, p65, E2F1, or p73 (Fig. 2D and Supplementary Fig. 3B–F). KO or KD of *p53* or *PUMA* largely suppressed OSW-1-induced necroptosis in HCT116, LoVo, and RKO cells as indicated by increased IC_50_ and crystal violet staining, but reduced PI staining, ATP depletion, p-MLKL, and HMGB1 release, and changes in cell morphology (Fig. 2D–J and Supplementary Fig. 4A–E). OSW-1-induced necroptosis was restored by knock-in of WT *p53* in *p53*-mutant DLD1 cells (Supplementary Fig. 4F, G) or reconstituting PUMA in *PUMA*-KO HCT116 cells (Fig. 2K, L). In contrast to *PUMA* KO, KO of other p53 downstream apoptotic mediators, including *Noxa*, *Bid*, *Bim*, *BAX*, and *BAK*, did not affect OSW-1-induced necroptosis (Supplementary Fig. 4H). PUMA induces mitochondrial damage during apoptosis or necroptosis [17, 18]. Interestingly, we did not detect classical apoptotic markers in OSW-1-induced cell death, such as activation of initiator caspases 8 and 9, Bax multimerization, or substantial cytosolic cytochrome *c* release (Supplementary Fig. 4I–K). These results demonstrate that OSW-1-induced cell death is mediated by p53-dependent transcriptional upregulation of *PUMA*, leading to RIP1/RIP3-independent necroptosis rather than apoptosis.

### OSW-1 promotes p53 stabilization and K120 acetylation to upregulate *PUMA* transcription and induce necroptosis

**Fig. 3:**
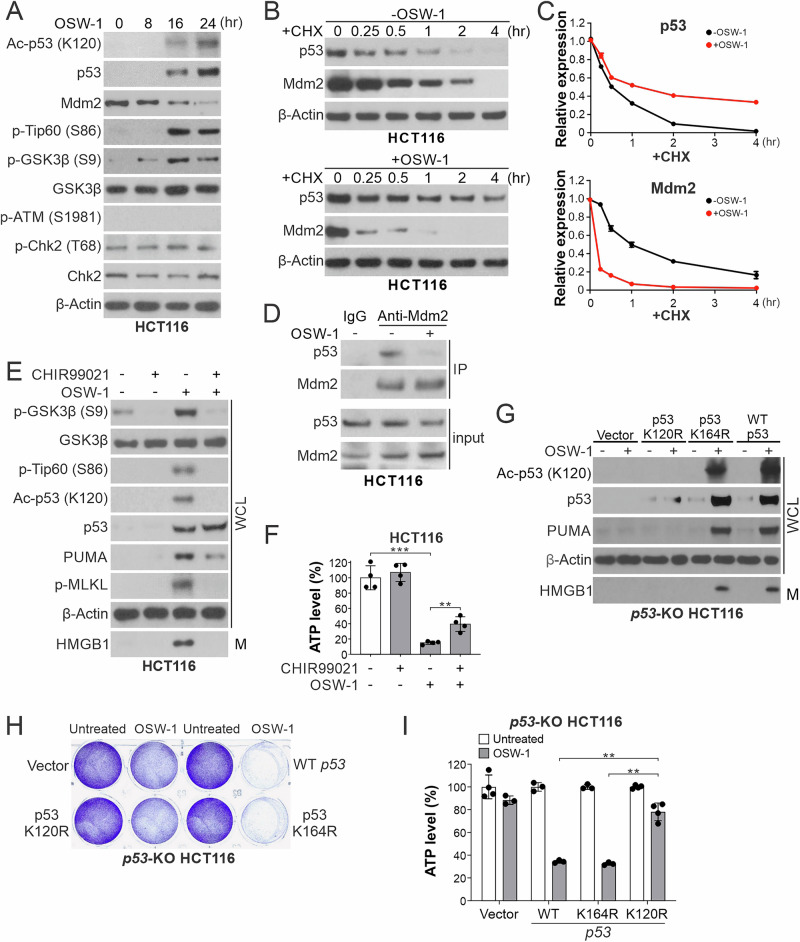
OSW-1 stabilizes p53 and promotes its K120 acetylation to selectively upregulate *PUMA* transcription and induce necroptosis. **A** Western blotting of indicated proteins in HCT116 cells treated with OSW-1 (0.5 nM) at indicated time points. **B**, **C** HCT116 cells were treated with OSW-1 (0.5 nM) along with the translation inhibitor cycloheximide (CHX; 10 μg/mL). **B** Western blotting of indicated proteins at indicated time points. **C** Quantification of western blots by NIH Image J and normalized to the loading control β-actin. **D** HCT116 cells with or without OSW-1 (0.5 nM) treatment for 24 h were subjected to immunoprecipitation (IP) to pull down Mdm2 followed by western blotting of indicated proteins. IgG was used as a negative control. **E**, **F** HCT116 cells were treated for 24 h with OSW-1 (0.5 nM) alone or in combination with the GSK3 inhibitor CHIR99021 (10 μM). **E** Western blotting of indicated proteins in whole cell lysates (WCL) and HMGB1 in 20-μl cell culture medium (M). **F** ATP levels in treated cells. **G**–**I** *p53*-KO HCT116 cells were transfected with control empty vector, WT, K164R, or K120R mutant p53 were treated with OSW-1 as in (**D**). **G** Western blotting of indicated proteins. **H** Crystal violet staining of viable cells. **I** ATP levels in treated cells. Quantitative results in (**F**) and (**I**) were expressed as means ± s.d. of four independent experiments. **, *P* < 0.01; ***, *P* < 0.001.

The rapid induction of p53 by OSW-1 is not associated with the classical DNA damage response [28], as shown by lack of increased phosphorylation of histone H2AX (S139), ATM (S1981), and Chk2 (T68) (Fig. 3A and Supplementary Fig. 5A). Mdm2, an E3 ubiquitin ligase and negative regulator of p53 stability, was downregulated at the protein but not mRNA level (Fig. 3A and Supplementary Fig. 5B). OSW-1 treatment extended the half-life of p53 determined by cycloheximide (CHX) chase, while decreasing that of Mdm2 (Fig. 3B, C), and also disrupted the p53/Mdm2 complex detected by immunoprecipitation (Fig. 3D).

Post-translational modifications (PTMs) of p53 are important for cell fate determination and target expression [29]. We detected markedly increased p53 lysine 120 (K120) acetylation, a PTM involved in p53/PUMA-mediated apoptosis and regulated by glycogen synthase kinase 3β (GSK3β)-dependent phosphorylation of Tip60 (S86), an acetyltransferase [30], in OSW-1-treated cells (Fig. 3A). GSK3 inhibition by CHIR99021 suppressed OSW-1-induced Tip60 phosphorylation, p53 K120 acetylation, PUMA upregulation, and necroptotic death (Fig. 3E, F). The functional role of p53 K120 acetylation was further determined by reconstituting *p53*-KO HCT116 cells with WT, acetylation-deficient K120R, or control K164R mutant (Fig. 3G). WT p53 and K164R mutant, but not K120R mutant, restored PUMA upregulation and cell death in *p53*-KO cells treated with OSW-1 (Fig. 3G–I). Together, these results indicate that OSW-1 downregulates Mdm2 to stabilize p53 and promotes p53 K120 acetylation to selectively upregulate *PUMA* transcription and induce necroptosis.

### OSW-1 induces p53 K120 acetylation and necroptosis via OSBP/ORP4 protein degradation and ER stress

**Fig. 4:**
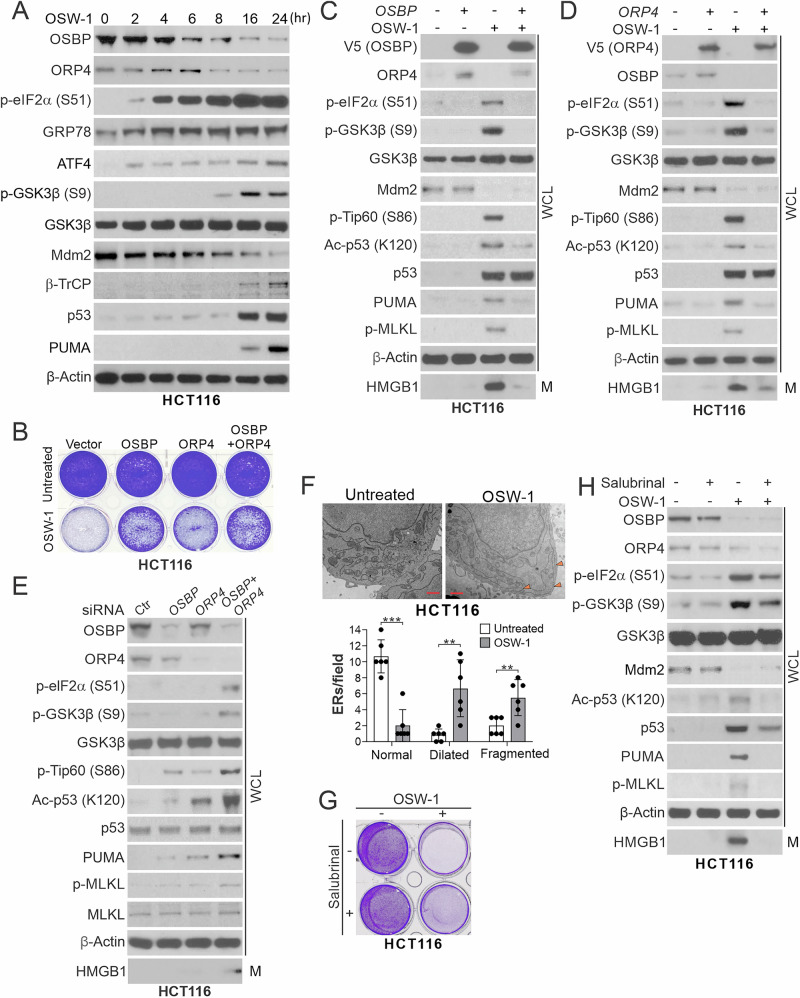
OSW-1 induces OSBP and ORP4 protein degradation and promotes ER stress to induce p53 K120 acetylation and necroptosis. **A** Western blotting of indicated proteins in HCT116 cells treated with OSW-1 (0.5 nM) at indicated time points. **B**–**D** HCT116 cells transfected with empty vector, OSBP, ORP4, or both OSBP and ORP4 were treated with OSW-1 (0.5 nM) for 24 h. **B** Crystal violet staining of viable cells. Western blotting of indicated proteins in whole cell lysates (WCL) and HMGB1 in 20-μl cell culture medium (M) from cells transfected with **C** OSBP or **D** ORP4. **E** Western blotting of indicated proteins in HCT116 cells transfected with control (Ctr), *OSBP*, *ORP4*, or both *OSBP* and *ORP4* siRNAs for 48 h. **F** TEM analysis of HCT116 cells treated with OSW-1 as in (**B**). *Upper*, representative TEM pictures with arrowheads indicating ERs (Scale bars, 1 μm); *lower*, quantification of dilated, fragmented, and normal ERs. Results were expressed as means ± s.d. from counting ERs in six randomly selected fields. **G**, **H** HCT116 cells were treated for 24 h with OSW-1 (0.5 nM) alone or in combination with Salubrinal (5 μM). **G** Crystal violet staining of viable cells. **H** Western blotting of indicated proteins. ** *P* < 0.01; *** *P* < 0.001.

OSW-1 has a steroidal moiety (Supplementary Fig. 1A) and can bind to intracellular membranes and target OSBP and ORP4 and disrupt lipid transport through ER and Golgi [31, 32]. OSW-1 treatment resulted in time-dependent downregulation of OSBP and ORP4 proteins, but not mRNA, along with p53/PUMA induction and Mdm2 degradation (Fig. 4A and Supplementary Fig. 5C). Transfection of OSBP and/or ORP4 rescued cell viability and inhibited OSW-1-induced p-MLKL and HMGB1 release, as well as GSK3β and Tip60 phosphorylation, p53 K120 acetylation, and PUMA induction (Fig. 4B–D). Conversely, KD of both OSBP and ORP4 cooperatively induced GSK3β and Tip60 phosphorylation, p53 K120 acetylation, and PUMA upregulation to levels sufficient to trigger p-MLKL and HMGB1 release (Fig. 4E).

We also detected time-dependent induction of ER stress response markers upon OSW-1 treatment, including phosphorylation of eIF2α (S51) and accumulation of GRP78 (Bip) and ATF4 (Fig. 4A). This response was suppressed by OSBP or ORP4 transfection (Fig. 4C, D), and recapitulated by OSBP and ORP4 KD (Fig. 4E). TEM analysis revealed morphological features of ER stress including dilated and fragmented ER in OSW-1-treated cells (Fig. 4F). Inhibiting ER stress using the p-eIF2α inhibitor Salubrinal [33] rescued cell viability and suppressed OSW-1-induced GSK3β phosphorylation, p53 K120 acetylation, PUMA induction, as well as p-MLKL and HMGB-1 release (Fig. 4G, H), suggesting that necroptosis induction via ER stress is an on-target effect of OSW-1.

Interestingly, OSBP/ORP4 transfection and ER stress inhibition only suppressed p53 K120 acetylation, but not p53 accumulation and Mdm2 degradation (Fig. 4C, D and Supplementary Fig. 5D, E), suggesting regulation of these events by a different pathway. We therefore analyzed Mdm2 regulators and found that its E3 ubiquitin ligase β-TrCP was induced at the time of Mdm2 degradation and p53 induction (Fig. 4A). KD of β-TrCP suppressed Mdm2 degradation and p53 induction by OSW-1 (Supplementary Fig. 5F). These results identified two independent arms of p53 activation, including β-TrCP-mediated Mdm2 degradation leading to p53 accumulation, and OSBP/ORP4 depletion and subsequent ER stress response resulting in GSK3β/Tip60-mediated p53 K120 acetylation, both of which are necessary for selective induction of PUMA and necroptosis.

### OSW-1-induced necroptosis involves CaMKIIδ-mediated MLKL phosphorylation

**Fig. 5:**
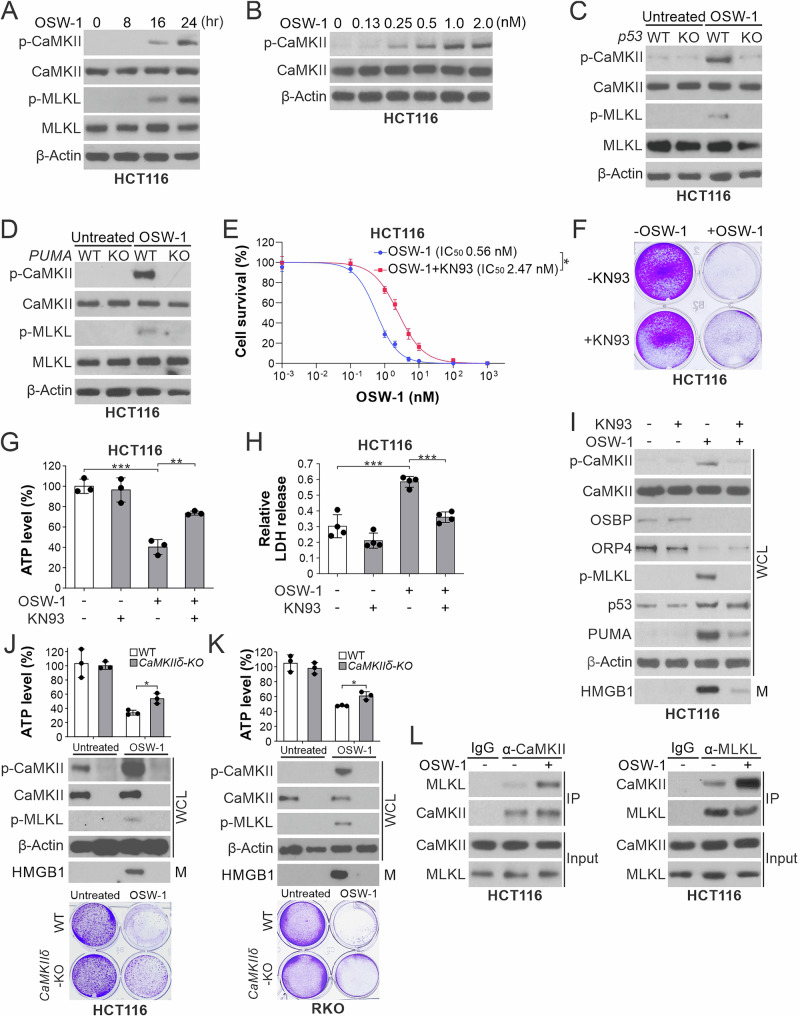
OSW-1-induced necroptosis is executed by CaMKIIδ-mediated MLKL phosphorylation. **A** Western blotting of indicated proteins in HCT116 cells treated with OSW-1 (0.5 nM) at indicated time points. **B** Western blotting of indicated proteins in HCT116 cells treated with OSW-1 at indicated concentrations for 24 h. **C**, **D** Western blotting of indicated proteins in (**C**) WT and *p53*-KO, **D** WT and *PUMA*-KO HCT116 cells treated with OSW-1 (0.5 nM) for 24 h. **E** MTS analysis of HCT116 cells treated for 48 h with OSW-1 at indicated concentrations alone or in combination with the CaMKII inhibitor KN93 (1 μM). **F**–**I** HCT116 cells were treated for 24 h with OSW-1 (0.5 nM) alone or in combination with KN93 (1 μM). **F** Crystal violet staining of viable cells. **G** ATP levels in treated cells. **H** LDH release from treated cells. **I** Western blotting of indicated proteins in whole cell lysates (WCL) and HMGB1 in 20-μl cell culture medium (M). **J**, **K **WT and *CaMKIIδ*-KO **J** HCT116 and **K** RKO cells were treated with OSW-1 as in (**C**). Necroptosis was analyzed by measuring ATP levels (*upper*), Western blotting of indicated proteins (*middle*), and crystal violet staining (*lower*). **L** HCT116 cells treated with OSW-1 as in (**C**) were subjected to IP to pull down CaMKII (*left*) or MLKL (*right*), followed by western blotting of indicated proteins. IgG was used as a negative control. Quantitative results in (**E**, **G**, **H**, **J**, **K**) were expressed as means ± s.d. of three independent experiments. *, *P* < 0.05; **, *P* < 0.01; ***, *P* < 0.001.

We investigated the mechanism by which PUMA promotes RIP1/RIP3-independent p-MLKL in OSW-1-induced necroptosis. OSW-1-induced cell death involved p53/PUMA-dependent mitochondrial calcium influx (Supplementary Fig. 6A, B) [34], which can potentially activate calcium/calmodulin-dependent protein kinase II (CaMKII) [35]. Indeed, we detected time- and dose-dependent CaMKII phosphorylation (p-CaMKII, T286) along with p-MLKL (Fig. 5A, B), which required p53 and PUMA (Fig. 5C, D), and was suppressed by OSBP or ORP4 transfection (Supplementary Fig. 6C). CaMKII inhibition by KN93 abrogated OSW-1-induced mitochondrial calcium influx (Supplementary Fig. 6D, E), rescued both short-term and long-term cell viability (Fig. 5E, F and Supplementary Fig. 6F), and suppressed necroptotic death (Fig. 5G–I). But KN93 did not affect upstream OSBP and ORP4 depletion and p53 induction (Fig. 5I).

Among 4 *CaMKII* isoforms (α, β, δ, and γ) [35], we only detected δ and γ in HCT116 cells by RT-PCR (Supplementary Fig. 6G, H). CaMKIIδ is more abundant, and KO or KD of *CaMKIIδ* suppressed OSW-1-induced cell death, p-MLKL, and HMGB1 release in HCT116 and RKO cells (Fig. 5J, K, and Supplementary Fig. 6I–K). However, the contribution of CaMKIIδ to cell death appears to be limited, as *CaMKIIδ* KO did not restore cell viability to the same extent as *p53* or *PUMA* KO (Fig. 2G). OSW-1 treatment enhanced the binding of CaMKII to MLKL, detected by reciprocal IP (Fig. 5L). Interestingly, CaMKII inhibition or *CaMKIIδ* KD not only suppressed mitochondrial calcium influx (Supplementary Fig. 6L, M) but also reduced the induction of PUMA, but not p53 (Fig. 5I and Supplementary Fig. 6K), suggesting a feedforward mechanism involved in OSW-1-induced necroptosis.

### OSW-1 suppresses tumor growth and induces antitumor immune response in a p53/PUMA-dependent manner

Necroptosis is a form of immunogenic cell death (ICD) due to the release of HMGB1, ATP, and other damage associated molecular pattern (DAMP) molecules. Treating CRC cells with OSW-1 markedly induced mRNA expression of *TNFA* and *IFNB1*, along with cell-surface level of the ER chaperone calreticulin (CRT), a pro-phagocytic signal to promote DC maturation, antigen presentation, and cytotoxic T cell priming [36], in a PUMA-dependent manner (Supplementary Fig. 7A, B). Co-culture of OSW-1-treated HCT116 cells with dendritic cells (DCs) differentiated from human peripheral blood mononuclear cells (PBMCs) from healthy donors induced DC phagocytosis of HCT116 cells, which was abolished by *p53* or *PUMA* KO (Supplementary Fig. 7C–E).

**Fig. 6:**
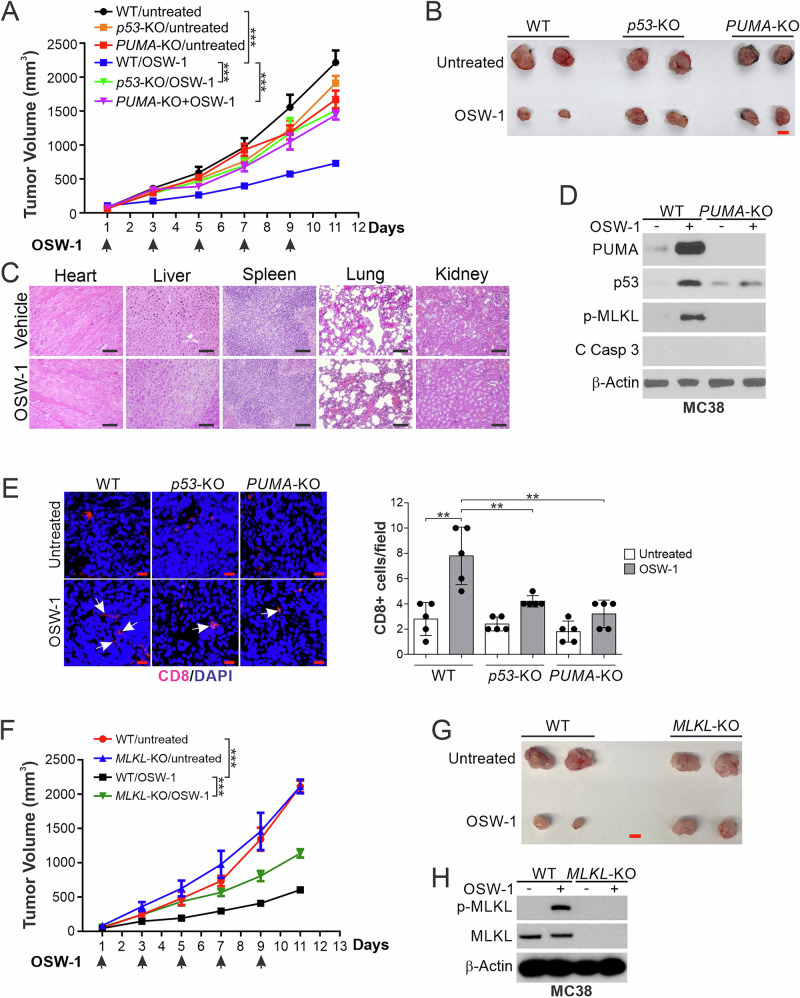
The in vivo antitumor activity of OSW-1 is mediated by p53/PUMA/MLKL-dependent necroptosis. **A**–**D** C57BL/6 mice were injected s.c. with 5 × 10^5^ WT, *p53*-KO, or *PUMA*-KO MC38 cells (n = 9–10 in each group). After 7 days, mice were treated with OSW-1 (i.p.; 12.5 μg/kg) every other day as indicated by arrows. **A** Tumor volume at indicated time points after treatment with statistical significance for indicated comparisons. **B** Representative tumors at the end of the experiment. Scale bars, 10 mm. **C** H&E staining of indicated organ tissues. Scale bars, 100 μm. **D** Western blotting of indicated proteins. **E** Paraffin-embedded tumor tissues from mice treated as in (**A**) and resected at day 11 were analyzed by immunostaining for CD8. *Left*, representative staining pictures with arrows indicating example cells with positive staining (Scale bars, 20 μm); *right*, quantification of CD8^+^ cells (n = 5 in each group). At least 300 nuclei from 3 randomly selected fields were counted for each tumor. **F**–**H** C57BL/6 mice were injected with WT or *MLKL*-KO MC38 cells and treated with OSW-1 as in (**A**) (n = 7 in each group). **F** Tumor volume at indicated time points after treatment with statistical significance for indicated comparisons. **G** Representative tumors at the end of the experiment. Scale bars, 10 mm. **H** Western blotting of indicated proteins. In (**D**, **H**), pooled lysates from three randomly selected tumors in each group were analyzed. **, *P* < 0.01; ***, *P* < 0.001.

We then used the MC38-C57BL/6 syngeneic tumor model to analyze the in vivo antitumor effects of OSW-1. At 12.5 μg/kg, OSW-1 markedly suppressed the growth of MC38 tumors in C57BL/6 mice without animal death, weight loss, or substantial cell loss in major organs (Fig. 6A–C and Supplementary Fig. 8A–C). Tumor suppression by OSW-1 was accompanied by p53 and PUMA induction and p-MLKL but without caspase activation (Fig. 6D). We detected infiltration of CD3+ and CD8+ T lymphocytes, as well as CD11c+ DCs (Fig. 6E and Supplementary Fig. 8D, E), and mRNA induction of *TNFA* and *IFNB1* in the tumors and spleens of OSW-1-treated mice (Supplementary Fig. 8F, G). *p53* or *PUMA* KO by CRISPR in MC38 cells abrogated the antitumor activity of OSW-1 and suppressed its necroptotic and immunogenic effects (Fig. 6A–E and Supplementary Fig. 8A–G). *MLKL* KO also suppressed the antitumor activity of OSW-1 and OSW-1-induced release of nuclear HMGB1 in MC38 tumors (Fig. 6F–H and Supplementary Fig. 9A–C). In nude mice, OSW-1 at up to 15 μg/kg did not show activity against HCT116 xenograft tumors, and escalation to 20 μg/kg led to animal death.

In addition, the in vivo efficacy of OSW-1 could be improved by nanoparticle packaging, which lowered the safe and effective dose of OSW-1 to 5 μg/kg against MC38 tumors in C57BL/6 mice (Supplementary Fig. 10A–C). OSW-1 in nanoparticles at 5–10 μg/kg was also effective against CT26 tumors established in BALB/c mice (Supplementary Fig. 10D, E).

### OSW-1 potentiates anti-PD-1 immunotherapy in a p53/PUMA-dependent manner

**Fig. 7:**
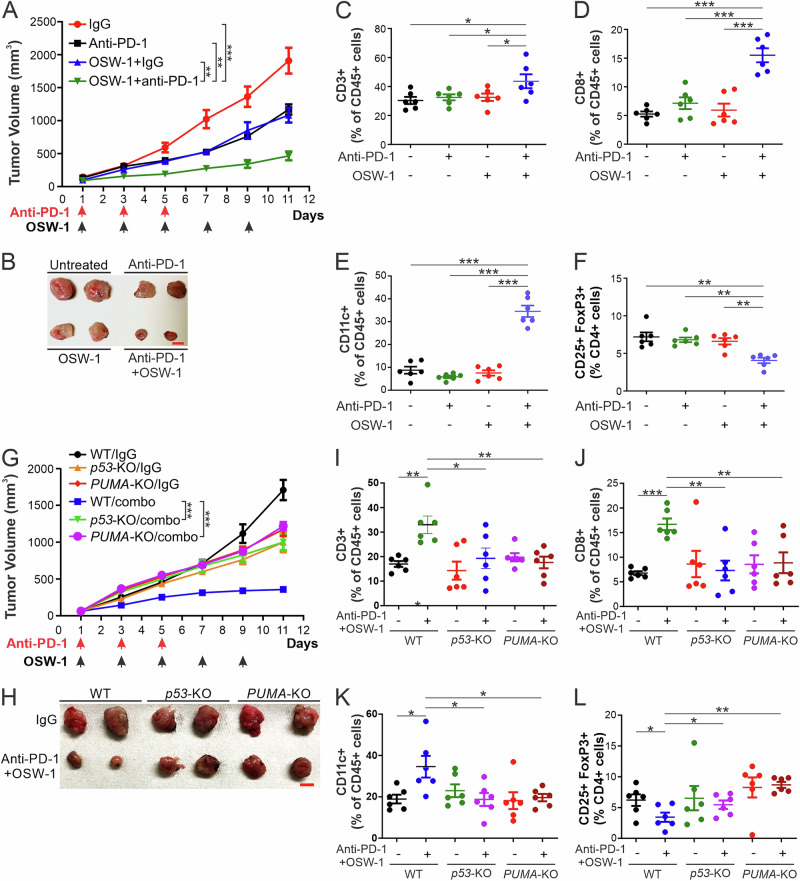
OSW-1 combined with anti-PD-1 antibody enhances tumor suppression and antitumor immune response. **A**–**F** C57BL/6 mice were injected s.c. with 5 × 10^5^ WT MC38 cells. After tumor growth for 7 days, mice were treated with OSW-1 (i.p.; 5 μg/kg), anti-mouse-PD-1 (i,p.; 150 μg/dose), or their combination as indicated in (**A**). **A** Tumor volume at indicated time points with statistical significance for indicated comparisons (n = 11–12 in each group). **B** Representative tumors at the end of the experiment. Scale bars, 10 mm. **C**–**F** Flow cytometry analysis of infiltrating lymphocytes in tumors resected on day 11 (n = 6 in each group): **C** CD3^+^/CD45^+^, **D** CD8^+^/CD45^+^, **E** CD11c^+^/CD45^+^, and **F** CD25^+^/FoxP3^+^/CD4^+^. **G**–**L** C57BL/6 mice were injected s.c. with 5 × 10^5^ WT, *p53*-KO, or *PUMA*-KO MC38 cells. After tumor growth for 7 days, mice were treated with OSW-1 (i.p.; 5 μg/kg), anti-mouse-PD-1 (i,p.; 150 μg/dose), or their combination as indicated in (**G**). **G** Tumor volume at indicated time points with statistical significance for indicated comparisons (n = 7–12 in each group). **H** Representative tumors at the end of the experiment. Scale bars, 10 mm. **I**–**L** Flow cytometry analysis of infiltrating lymphocytes in tumors resected on day 11 (n = 6 in each group): **I** CD3^+^/CD45^+^, **J** CD8^+^/CD45^+^, **K** CD11c^+^/CD45^+^, and **L** CD25^+^/FoxP3^+^/CD4^+^. *, *P* < 0.05; **, *P* < 0.01; **, *P* < 0.001.

Most CRCs do not respond to immunotherapy. We found the PD-1 ligand PD-L1 is depleted in cells undergoing OSW-1-induced necroptosis (Supplementary Fig. 3A). We therefore investigated if OSW-1 can sensitize CRC to anti-PD-1 immunotherapy. C57BL/6 mice bearing MC38 tumors were treated with anti-mouse PD-1 antibody at a dose (150 μg/dose i.p.) and schedule (3 every-other-day doses; Fig. 7A) previously described [37], with or without OSW-1 at a reduced dose (5 μg/kg). We observed a striking synergism between OSW-1 and anti-mouse PD-1 while either agent alone showed little efficacy (Fig. 7A, B and Supplementary Fig. 11A), along with significant extension of animal survival (Supplementary Fig. 11B). The combination was well tolerated without affecting body weight (Supplementary Fig. 11C). The combination treatment significantly increased infiltration of CD3+ T cells, CD8+ T cells, and CD11c+ DCs, and decreased infiltration of CD25+/FoxP3+ immunosuppressive regulatory T cells (Tregs) with no significant changes in CD4+ T cells in WT tumors (Fig. 7C–F and Supplementary Fig. 11D–G). Remarkably, the observed therapeutic and immunogenic effects were largely inhibited by *p53* and *PUMA* KO in MC38 tumors (Fig. 7G–L and Supplementary Fig. 12A–F), indicating a critical role of *p53*/*PUMA*-dependent cell death in mediating the antitumor immune response to this combination. These results indicate that OSW-1-induced and p53/PUMA-mediated necroptosis has potent antitumor and immunogenic effects, which can potentially be harnessed to improve CRC therapeutic response.

### The therapeutic effects of OSW-1 require antitumor immunity

We further evaluated the contribution of immune response to OSW-1-induced antitumor response in vivo. Depleting CD8+ T cells abolished the effects of OSW-1 on WT MC38 tumors (Supplementary Fig. 13A, B), confirming the role of CD8+ T cells in tumor suppression. WT MC38 tumors established in immunodeficient NSG mice were completely insensitive to OSW-1 alone or in combination with anti-PD-1 (Supplementary Fig. 13C–F), indicating that the in vivo activity of OSW-1 is dependent on antitumor immune response. Together, these results demonstrate that the in vivo antitumor effect of OSW-1 is mediated by p53/PUMA-dependent necroptosis and ensuing antitumor immune response.

## Discussion

Our results shed new insights into the highly potent anticancer activity of OSW-1 (Supplementary Fig. 14), which primarily induces p53/PUMA-mediated and RIP1/RIP3-independent necroptosis to kill CRC cells. Mechanistically, OSW-1 upregulates β-TrCP to degrade Mdm2 and stabilize p53, while depletion of OSBP and ORP4 triggers ER stress and GSK3β/Tip60-mediated p53 K120 acetylation. This leads to selective induction of PUMA, mitochondrial calcium influx, and CaMKIIδ-mediated MLKL phosphorylation, and eventually necroptotic death. Importantly, cell death induced by OSW-1 is highly immunogenic and potentiates cancer immunotherapy.

Despite several studies suggesting that OSW-1 induces apoptosis [21, 22, 34], our results clearly show that necroptosis, rather than apoptosis, is the major cell-killing mechanism of OSW-1. Our previous study showed that radiation-induced p53 with K120 acetylation can upregulate PUMA to induce apoptosis in mouse intestinal crypts [30]. PUMA was also found to contribute to ER stress-induced and p53-independent apoptosis in pancreatic β cells and neuronal cells [38, 39]. The differences in cell death phenotypes among these studies could be attributed to variations in p53 modifications, contributions from other cell death pathways, cell death inducers, and the cellular context. Lack of apoptosis in OSW-1-treated colon cancer cells could be explained by the inability of p53 to induce its downstream proapoptotic targets other than PUMA, including Noxa, Bim, Bid, and Bax. Therefore, the apoptotic signal does not reach a threshold level for triggering caspase activation and other apoptotic events such as Bax/Bak mitochondrial translocation and multimerization, mitochondrial outer membrane permeabilization (MOMP), and cytochrome *c* release. On the other hand, the induction of PUMA by OSW-1 almost exclusively drives necroptosis. PUMA was initially identified as a proapoptotic protein [18]. However, accumulating evidence indicates that PUMA primarily engages necroptosis under certain circumstances, such as cell death induced by TNF-α and SMAC mimetics in HT29 CRC cells [17], by 5-FU and other antimetabolites in some RIP3-expressing CRC cells [19], and by acetaminophen in mouse hepatocytes [40]. A critical role of PUMA in physiological and pathological necroptosis is also supported by animal model studies [17, 40].

PUMA mediates OSW-1-induced necroptosis by promoting mitochondrial calcium influx and calcium-mediated signaling, which was shown to be involved in OSW-1-induced cell death [34]. It is well known that calcium influx enhances the binding of calcium ions to the calcium sensor calmodulin, inducing its conformational change and activation. Activated calmodulin can, in turn, bind to CaMKII, promoting its phosphorylation and subsequent activation [41]. In addition to PUMA, calcium homeostasis and the ER stress response are also regulated by other BH3-only proteins such as Bim [42, 43]. The role of PUMA in OSW-1-induced calcium release may be attributed to its specific upregulation and/or unique biochemical activities. It has been shown that PUMA, but not Noxa, can increase intracellular calcium levels by inducing calcium release from the ER [44]. However, the exact mechanism by which PUMA modulates the calcium homeostasis is unclear, which appears to be independent of MOMP. Mitochondrial calcium inflex is a hallmark of dying cells and can cause accumulation of reactive oxygen species (ROS) and trigger the release of necrotic factors [45]. Interestingly, a PUMA-mediated feedforward amplification loop via calcium signaling seems to be involved in OSW-induced necroptosis (Fig. 5I, K). A similar PUMA-mediated signal amplification mechanism was also found in TNF-α-mediated necroptosis [17]. The mechanism by which PUMA amplifies necroptosis signal remains to be further characterized.

Our results indicate that MLKL phosphorylation and necroptosis induction can bypass the necrosome complex formed by RIP1 and RIP3. Just as apoptosis, necroptosis can be initiated via a cell extrinsic or intrinsic pathway. While most of previous studies focused on the extrinsic pathway involving TNF and death receptor signaling, severe intracellular stress such as ER/Golgi perturbation can also trigger necroptosis. In this case, it is CaMKIIδ, rather than RIP1 and RIP3, that mediates MLKL phosphorylation. In line with our findings, recent studies showed that CaMKII can promote RIP3-independent MLKL phosphorylation in response to serum and amino acid starvation [46, 47]. While CaMKII-mediated MLKL phosphorylation executes necroptosis in colon cancer cells treated with OSW-1 (Fig. 5) and smooth muscle cells treated with TNF-α plus z-VAD [46], it was found to facilitate autophagic flux and protect cells from starvation-induced death in L929 mouse fibroblast cells [47]. This protective effect may be due to the absence of additional stresses, such as ER stress and oncogenic stress, in the starved L929 cells. Additionally, CaMKII can also function as a key substrate of RIP3 in mediating ischemia- and oxidative stress-induced myocardial necroptosis [35]. These studies indicate that CaMKII-mediated signaling functions as a nodal for integrating multiple programmed cell death pathways and a potential therapeutic target.

OSW-1-induced and p53/PUMA-mediated necroptosis is an on-target effect of depleting the OSW-1 receptors OSBP and ORP4, though the underlying mechanism remains to be determined [23]. Dysregulation of the ORP family including OSBP and ORP4 has been implicated in cancer [25]. For example, ORP4 plays a key role in the development of T-cell acute lymphoblastic leukemia (T-ALL) [26]. In T-ALL cells, OSBP and ORP4 form a dimer to regulate the exchange of lipids between Golgi apparatus and plasma membrane, which is critical for oncogenic signaling such as PI3K/AKT activation [27]. However, the role of OSBP and ORP4 in CRC has not been described. Analysis of public databases revealed elevated expression of OSBP and ORP4 in CRC, which is associated with favorable clinical outcomes. The selectivity of OSW-1 against CRC cells could be explained by the oncogenic functions of OSBP and ORP4 in CRC cells, which remain to be elucidated in future studies.

CRCs are often defective in anticancer therapy-induced necroptosis due to frequent silencing of RIP3 and other necroptosis regulators [11]. OSW-1 can bypass the requirement of RIP1 and RIP3 to promote MLKL phosphorylation and necroptosis, which is potentially useful for overcoming therapeutic resistance in CRC. OSW-1 also showed promising activity against other cancer types, including leukemia, pancreatic cancer, and hepatocellular carcinoma [22]. However, developing OSW-1 as an anticancer agent has been hampered by several obstacles. OSW-1 was not readily available until the development of its total synthesis strategies [21, 48]. The in vivo efficacy and pharmacological properties of OSW-1 have not been well characterized largely due to dose-limiting toxicity in immune-deficient tumor models. Packaging OSW-1 in nanoparticles is potentially useful for reducing its effective dose and improving its therapeutic window. Chemical modifications of OSW-1 may also help improve its efficacy and reduce toxicity. Furthermore, the extreme potency of OSW-1 suggests a potential use as a payload for developing antibody-drug conjugates (ADCs), which have shown great promise in recent studies [49].

OSW-1-induced and p53/PUMA-mediated necroptosis is highly immunogenic and can prime the activation of DCs to engage adaptive immunity. Our results demonstrate potent in vivo efficacy and therapeutic window of OSW-1 in immunocompetent syngeneic mice. Importantly, the in vivo antitumor activity of OSW-1 largely relied on the immune response and was abolished by depletion of CD8+ T cells or in immunodeficient host. OSW-1 also potentiated CRC to anti-PD-1 immunotherapy. The clinical benefit of immune checkpoint inhibitors (ICIs) is currently limited to 10–15% of CRCs that are deficient in DNA mismatch repair [50]. A burning issue is to convert immunologically “cold” tumors into “hot” ones [51]. Our results provide a proof-of-principal for utilizing a necroptosis-inducing natural compound to improve the efficacy of PD-1 blockade. This may represent a promising strategy for targeting immune escape of CRC and those that are insensitive to ICIs including anti-PD-1, anti-PD-L1, and anti-CTLA4 antibodies.

In summary, we identified a novel p53/PUMA-mediated and RIP1/RIP3-independent necroptosis pathway underlying the extremely potent anticancer activity of OSW-1, which can be harnessed to develop new anticancer therapies by inducing antitumor immunity.

## Materials and methods

### Cell culture and drug treatment

Cell lines and sources are described in Supplementary Table 1. Authentication of parental cell lines was based on analysis of DNA mismatch repair (MMR) status and *p53*, *KRAS*, *BRAF*, *PIK3CA*, *FBW7*, and other driver mutations [52]. Cell lines were regularly checked for morphology and absence of mycoplasma contamination. Human CRC cell lines were cultured in McCoy’s 5A modified media (Invitrogen), and MC38 murine CRC cells were cultured in DMEM media (Invitrogen). Non-malignant human colonic epithelial cell line NCM356 was cultured in M3: Base A media (Incell). Cell culture media were supplemented with 10% Premium FBS (Atlanta Biologicals) and 100 units/ml penicillin plus 100 µg/ml streptomycin (Invitrogen). Cells were cultured in a 37 °C incubator at 5% CO_2_. Anticancer agents and chemicals used are listed in Supplementary Table 1. Stock solutions of all agents were prepared in DMSO and further diluted in culture media to final concentrations.

### Analysis of cell survival and death

Cells cultured overnight in 96-well plates at a density of 1 × 10^4^ cells/well were treated with OSW-1 for 48 h. Cell survival was assayed by 3-(4,5-dimethylthiazol-2-yl)-5-(3-carboxymethoxyphenyl)-2-(4-sulfophenyl)-2H-tetrazolium (MTS) assay (Promega) as described [37]. ATP level was measured using the CellTiter-Glo Luminescent Cell Viability Assay kit (Promega). Caspase activity was measured using the SensoLyte Homogeneous AMC Caspase-3/7 Assay kit (AnaSpec). LDH release was analyzed using 50-μl cell culture medium and the CytoTox 96 Non-Radioactive Cytotoxicity Assay kit (Promega), with the results normalized to the Maximum LDH Release Control. All assays were performed according to the manufacturer’s instructions. Chemiluminescence and absorbance were read on a Wallac Victor 1420 Multilabel Counter (Perkin Elmer). Each assay was conducted in triplicate.

Cells cultured overnight in 12-well plates at a density of 40-50% were treated with OSW-1 or other drugs for 24–48 h. Viable cells were visualized by staining with crystal violet solution (0.05% crystal violet in 3.7% Paraformaldehyde diluted in distilled water). Cell death was analyzed by cell staining with Annexin V/PI followed by flow cytometry (BD Accuri C6) as previously described [17]. Apoptosis was assayed by counting cells containing condensed and fragmented nuclei after nuclear staining of adherent and floating cells with Hoechst 33258 [53]. Cytosolic release of mitochondrial cytochrome *c* was analyzed by western blotting of cytoplasmic and mitochondrial fractions prepared by using the Mitochondrial Fractionation Kit (Active Motif) according to the manufacturer’s instructions. Ultrastructure of OSW-1-treated cells was analyzed by TEM using a JEOL JEM 1400 FLASH (JEOL USA) for image acquisition as described [17]. Long-term cell viability was determined by colony formation assay [54]. Briefly, 1 × 10^3^ drug-treated cells were plated into 6-well plates in drug-free medium at appropriate dilutions. After 14 days, colonies were detected by crystal violet staining and counted. Each assay was conducted in triplicate.

### Analysis of protein and mRNA expression

Western blotting was performed using antibodies listed in Supplementary Table 1 as previously described [55]. Original blot pictures are shown in the Supplementary Material. Band intensities were quantified using NIH ImageJ software. Total RNA isolation was done using Mini RNA Isolation II Kit (Zymo Research) according to the manufacturer’s protocol. One-µg total RNA was used to make cDNA by using SuperScript III reverse transcriptase (Invitrogen). mRNA expression was analyzed by real-time reverse transcriptase PCR (RT-PCR) using primers listed in Supplementary Table 2 and previously described cycle conditions [17].

### Transfection, adenoviral infection, and expression constructs

Cells plated at 30–40% density in 12-well plates were used for transfection and adenoviral infection. Transfection was performed using 600 ng of plasmid constructs (Supplementary Table 1) or 200 pmol small interfering RNA (siRNA; Supplementary Table 2) with Lipofectamine 2000 (Invitrogen) according to the manufacturer’s protocols. Adenoviral infection was performed as previously described using PUMA-expressing adenovirus (Ad-PUMA) or control adenovirus with BH3 domain deletion (Ad-ΔBH3) [56].

OSBP and ORP4 expression constructs were generated by sub-cloning of corresponding cDNA sequences from pLJM1-FLAG-GFP-OSBP (Addgene #134659) and MGC Human OSBP2/ORP4 Sequence-Verified cDNA (Horizon Discovery MHS6278-211690367) into pcDNA3.1-V5 vector (ThermoFisher #V81020). p53 K120R and K164R mutant expression constructs were generated by sub-cloning of corresponding cDNA sequences from pGLS3-5×HRE-p53(K120R) (Addgene #72554) and pGLS3-5×HRE-p53(K164R) (Addgene #72555) into pcDNA3.1-HA vector (Addgene #128034). All constructs were verified by sequencing. Details of constructs were available upon request.

### Gene targeting by CRISPR/Cas9

*MLKL* in HCT116, RKO, and MC38 cells and *CaMKII*δ in HCT116 and RKO cells were targeted by CRISPR/Cas9 using the single guide RNA (sgRNA) sequences listed in Supplementary Table 2 as described [17]. Briefly, DNA duplexes with the sgRNA sequences were cloned into the pSpCas9-2A-GFP vector (Addgene; #48138). For gene targeting, cells were transfected with sgRNA vectors and harvested after 72 h. Single GFP-positive cells were isolated by fluorescence activated cell sorting (FACS) and seeded in 96-well plates. After 2–3 weeks, single cell clones were expanded and analyzed for gene targeting by western blotting for MLKL or CaMKII. Gene targeting was subsequently verified by sequencing of the targeted genomic regions.

### Luciferase reporter assays

Cells were transfected with the *PUMA* reporter construct pBV-Luc-Frag A along with the transfection control β-galactosidase reporter pCMVβ (Promega). Collection of cell lysates, measurement of the luciferase activities, and normalization to pCMVβ were performed as described [17]. Reporter experiments were performed in triplicate and repeated three times.

### Immunoprecipitation

Immunoprecipitation (IP) was performed as previously described [53]. Briefly, OSW-1-treated cells were harvested and suspended in 1 mL of EBC buffer (50 mmol/L Tris-HCl, pH 7.5, 100 mmol/L NaCl, 0.5% Nonidet P-40) supplemented with a protease inhibitor cocktail (Roche Applied Sciences). After cell lysis by sonication, cell lysates were prepared by centrifugation at 10,000 × *g* for 10 min. IP was done by incubating cell lysates with 1–2 µg of IP antibodies mixed with Protein G/A-agarose beads (Sigma) at 4 °C overnight. Beads were washed 3 times with PBS containing 0.02% Tween 20 (pH 7.4), boiled in 2× Laemmli sample buffer, and subjected to SDS-PAGE and western blot analysis.

### Analysis of mitochondrial calcium

Mitochondrial calcium was detected by fluorescence imaging or flow cytometry of cells stained with 1 μM Rhod-2 (Invitrogen), a cell-permeable fluorescent Ca^2^^+^ indicator, at 37 °C for 45 min. For fluorescence imaging, cells plated and treated in chamber slides (ThermoFisher) were stained with Rhod-2 and MitoTracker Green FM (ThermoFisher), washed 3 times with PBS, and analyzed by a Nikon A1 Confocal Laser Microscope. For flow cytometry, cells plated and treated in a 12-well plate were stained with Rhod-2, washed 3 times with PBS, and analyzed by a FACS Calibur flow cytometer.

### Analysis of immunogenic cell death (ICD)

ICD was analyzed by detecting calreticulin (CRT) cell-surface translocation and phagocytosis by dendritic cells (DCs) as described [37]. For cell-surface CRT, cells plated in 12-well plates (3 × 10^5^ cells/well) and treated with 0.5 nM OSW-1 for 24 h were stained with a rabbit anti-CRT antibody (1:1000; Supplementary Table 1), and then Alexa Fluor488 anti-rabbit (1:1000; Supplementary Table 1), followed by flow cytometry using ACCURI C6 (BD Biosciences) with gating of PI-negative cells. Isotype-matched IgG antibodies were used as a negative control.

For DC phagocytosis, CRC cells were labeled with 2 μM of carboxyfluorescein succinimidyl ester (CFSE; Invitrogen), and then seeded and treated with 0.5 nM OSW-1 for 24 h in chamber slides (ThermoFisher) for fluorescence imaging, or in 48-well plates (1 × 10^5^ cells/well) for flow cytometry. DCs were prepared by differentiating CD14+ cells isolated from PBMCs, followed by verifying DC maturity and purity by flow cytometry of CD14, CD86, HLA-DR, and CD83 as described [37]. DCs were labeled with 5 μM CellTrace Far Red dye (Invitrogen), incubated with human IgG (20 μg/L × 10^6^ cells) for 30 min, and then added to and co-cultured with OSW-1-treated cells for 2 h in a 1:1 ratio. DC phagocytosis was detected with double staining by flow cytometry (ACCURI C6) and confirmed by fluorescence microscopy with a Nikon A1 Confocal Laser Microscope using green (for CFSE) and magenta (for Far Red) channels.

### Animal experiments

All animal experiments were approved by the University of Pittsburgh or USC Institutional Animal Care and Use Committee. Sample size was estimated based on previous experience [57, 58]. Mice were housed in a sterile environment with micro isolator cages and allowed access to water and chow *ad libitum*. Xenograft tumors were established by injecting 5 × 10^5^ parental or derivative MC38 cells into one flank of 5–6 week-old female C57BL/6 mice (Jackson Laboratory), or both flanks of 5–6 week-old female NOD.Cg-*Prkdc*scid *Il2rgtm1Wjl*/SzJ (NSG) mice (Jackson Laboratory). All treated animals were analyzed without randomization and blinding to the group allocation.

After tumor growth for 7 days, tumor-bearing mice were treated with indicated drugs alone or in combination, including: OSW-1 (Gift from Dr. Zhendong Jin and also purchased from Caymen Chemical), intraperitoneal (IP), 5.0–12.5 µg/kg every other day; anti-mouse-PD-1 (BioXCell), IP, 150 µg/dose every other day for three doses; anti-mouse-CD8a (BioXCell), 300 µg/dose every other day for 3 doses. Animal body weights were recorded every other day. Tumor volumes were measured by calipers and calculated according to the formula ½ × length × width^2^. Ethical endpoint represents a time point when tumors reached 2 cm or more in any dimension. Tumors were collected at indicated time points and processed for analysis of necroptosis and immune markers.

### Immunostaining of tumor tissues

Tumor tissues were dissected and fixed in 10% formalin and embedded in paraffin. H&E staining was done with steps including dewaxing, dehydration, hematoxylin staining, differentiation, bluing, eosin, dehydration, and clearing. Immunostaining was performed on 5-μm paraffin-embedded tumor sections using kits and antibodies listed in Supplementary Table 1 as described [55]. Staining signals were detected with AlexaFluor 592-conjugated secondary antibody with 4′ 6-Diamidino-2-phenylindole (DAPI) for nuclear counter staining. Staining images were captured on Olympus XI71 fluorescence microscope.

### Flow cytometry analysis of tumor-infiltrating lymphocytes

Tumors were collected from mice treated with OSW-1 and/or anti-PD-1 antibody 24 h after the final treatment. Tumor tissues were suspended for 1 h at 37 °C with shaking in 5 mL of base RPMI-1640 media with 10× dissociation cocktail containing collagenase I, collagenase IV, and DNase I. Single-cell suspensions were prepared by passing cells through a 70-μm cell strainer. Cells were stained with combinations of fluorochrome-conjugated antibodies listed in Supplementary Table 1, including those for mouse CD16/32, CD45, CD3, CD4, CD8, CD11c, CD25, and FoxP3. Cells were subsequently stained with Zombie Aqua (Biolegend) for detecting viable cells, fixed in 1% formalin, and then subjected to flow cytometry (ACCURI C6).

### Preparation of OSW-1-loaded emulsion nanoparticles (NPs)

Packaging of OSW-1 in nanoparticles was performed similarly as previously described [59]. OSW-1 (0.4 mL in dichloromethane, 10 mg/mL), PEG_2000_-Fmoc_2_-OA_2_ (2.5 mL in dichloromethane, 10 mg/mL), soy phosphatidylcholines (1 mL in dichloromethane, 50 mg/mL), and sesame seed oil (2 mL in dichloromethane, 50 mg/mL) were mixed. The solvent was removed by a stream of nitrogen to generate a thin film at the bottom of the glass tube, and the residual organic solvent was further removed under vacuum for 1 h. Subsequently, the thin film was hydrated and suspended in DPBS, and sonication was applied for 10 min to obtain OSW-1-loaded emulsion NPs. The size of the NPs was ~150 nm with a polydispersity index (PDI) of 0.171.

### Statistical analysis

Statistical analyses were carried out using GraphPad Prism 10 software (GraphPad). For cell culture experiments, the results from multiple independent experiments were analyzed. *P* values were calculated using the Student’s *t*-test. For animal experiments, the results from multiple tumors in different animals in each group were analyzed. *P* values were calculated by repeated measures (RM) ANOVA with Fisher’s LSD post-hoc tests. Means ± standard deviation (s.d.) are reported in the figures. Differences were considered significant if *P* < 0.05.

## Supplementary information

Supplementary Figure Legends

Supplementary Figure 1-14

Supplementary Table 1

Supplementary Table 2

Original Western blot pictures

## Data Availability

All data are available from the corresponding authors upon reasonable request.
