# Supplementary material for: Depletion of oxysterol-binding proteins by OSW-1 triggers RIP1/RIP3-independent necroptosis and sensitization to cancer immunotherapy
Source: Cell Death Differ. 2025 May 6;32(11):2038–52. doi: 10.1038/s41418-025-01521-8 (PMC12572256; doi:10.1038/s41418-025-01521-8)
Supplement: Supplementary file 1 — Supplementary Figure Legends [file 41418_2025_1521_MOESM1_ESM.docx]

**Supplementary Figure Legends**

**Supplementary Fig. 1 OSW-1 induces non-apoptotic cell death in CRC cells. (A)** Chemical structure of OSW-1: 3β,16β,17α-trihydroxycholest-5-en-22-one 16-*O*-(2-*O*-4-methoxybenzoyl-β-D-xylopyranosyl)-(1→3)-2-*O*-acetyl-α-L-arabinopyranoside. **(B)**, **(C)** Western blotting of RIP1, RIP3, and MLKL in indicated CRC cell lines **(B)** without or **(C)** with OSW-1 (0.5 nM) treatment for 24 hr. **(D)** HCT116 and RKO cells treated with OSW-1 as indicated concentrations for 24 hr were analyzed for apoptosis by visualizing (*upper*) and counting (*lower*) cells containing condensed and fragmented nuclei after nuclear staining with Hoechst 33258. Cells treated with cisplatin (50 μM) for 24 hr were used as a positive control. Scale bars, 20 μm. **(E)** Caspase activation in HCT116 cells treated with OSW-1 as in (C) was analyzed by measuring caspase 3/7 activity (*upper*) and Western blotting of cleaved caspase 3 (C Casp 3; *lower*). Cells treated with cisplatin as in (D) were used as a positive control. **(F)** Crystal violet staining of HCT116 cells treated for 24 hr with OSW-1 (0.5 nM) alone or in combination with the pan-caspase inhibitor z-VAD-fmk (z-VAD; 10 μM), the RIP1 inhibitor Necrostatin-1 (Nec-1; 20 μM), and/or the MLKL inhibitor Necrosulfonamide (NSA; 2 μM). **(G)**-**(I)** Crystal violet staining (*left*) and ATP levels (*right*) of HCT116 cells treated for 48 hr with OSW-1 (0.5 nM) alone or in combination with **(G)** the autophagy inhibitor Balfilomycin A1 (200 nM), **(H)** the ferroptosis inhibitor Ferrostatin-1 (2 μM), or **(I)** the pyroptosis inhibitor Disulfram (10 μM). Quantitative results in (D), (E), and (G)-(I) are expressed as means ± s.d. of three independent experiments. *NS*, *P* >0.05; ******, *P* <0.01; *******, *P* <0.001.

**Supplementary Fig. 2 OSW-1 induces RIP1/RIP3-independent necroptosis in CRC cells.** **(A)** LDH release in indicated CRC cell lines and NCM356 cells treated with OSW-1 (0.5 nM) for 48 hr.

**(B)-(E)** ATP levels (*upper*) and Western blotting of indicated proteins (*lower*) in whole cell lysates (WCL) and HMGB1 in 20-μl cell culture medium (M) in **(B)** RKO, **(C)** Lim2405, **(D)** LoVo, and **(E)** Lim1215 cells treated for 24 hr with OSW-1 (0.5 nM) alone or in combination with the pan-caspase inhibitor z-VAD-fmk (z-VAD; 10 μM), the RIP1 inhibitor Necrostatin-1 (Nec-1; 20 μM), or the MLKL inhibitor Necrosulfonamide (NSA; 2 μM). **(F)** HCT116 cells transfected with control (Ctr) or *MLKL* siRNA were treated with OSW-1 (0.5 nM) for 24 hr. Necroptosis was analyzed by measuring ATP levels (*upper*), Western blotting of indicated proteins (*middle*), and crystal violet staining (*lower*). **(G)** Lim1215 cells transfected with Ctr or *RIP3* siRNA were treated with OSW-1 and analyzed for necroptosis as in (F). Quantitative results in (A)-(G) are expressed as means ± s.d. of three or four independent experiments. *NS*, *P* >0.05; *****, *P* <0.05; ******, *P* <0.01; *******, *P* <0.001.

**Supplementary Fig. 3 OSW-1-induced necroptosis involves p53-mediated PUMA induction. (A)** Western blotting of indicated proteins in HCT116 cells treated with OSW-1 (0.5 nM) at indicated time points. **(B)** Western blotting of indicated proteins in indicated *p53*-WT and *p53*-mutant CRC cell lines treated with OSW-1 (0.5 nM) for 24 hr. **(C)** Real-time RT-PCR analysis of *PUMA* mRNA expression in WT and *p53*-KO HCT116 cells treated with OSW-1 as in (B). **(D)** WT and *p53*-KO HCT116 cells were transfected overnight with a *PUMA* promoter reporter (pBV-Luc-Frag A), and then treated with OSW-1 as in (B). Reporter activities were measured and normalized to the untreated transfection control. **(E)** Western blotting of indicated proteins in HCT116 cells treated with OSW-1 as in (B). **(F)** Western blotting of indicated proteins in HCT116 cells transfected with control (Ctr) or *p65* siRNA and treated with OSW-1 as in (B). Quantitative results in (C) and (D) are expressed as means ± s.d. of three independent experiments**. *****, *P* <0.01.

**Supplementary Fig. 4 OSW-1-induced necroptosis is dependent on p53 and PUMA in CRC cells. (A)** MTS analysis of WT, *p53*-KO, and *PUMA*-KO LoVo cells treated with OSW-1 at indicated concentrations for 48 hr. **(B)**, **(C)** WT along with **(B)** *p53*-KO or **(C)** *PUMA*-KO LoVo cells were treated with OSW-1 (0.5 nM) for 24 hr. *Upper*, ATP levels in treated cells; *lower*, Western blotting of indicated proteins in whole cell lysates (WCL) and HMGB1 in 20-μl cell culture medium (M). **(D)**, **(E)** RKO cells transfected with control (Ctr), **(D)** *p53* or **(E)** *PUMA* siRNA were treated with OSW-1 and analyzed for necroptosis as in (B). **(F)**, **(G)** Parental and WT-*p53*-knock-in (*p53*-KI) DLD1 cells were treated with OSW-1 as in (B). **(F)** Analysis of necroptosis as in (B). **(G)** Crystal violet staining of viable cells. **(H)** WT, *BAX*-KO, *Bid*-KO, *BAK*-KO, *Bim*-KO, and *Noxa*-KO HCT116 cells were treated with OSW-1 as in (B). *Upper*, ATP levels in treated cells; *lower*, Western blotting of HMGB1 in 20-μl cell culture medium. **(I)** Western blotting of indicated proteins in HCT116 cells treated with OSW-1 (0.5 nM) at indicated time points. Cells treated with 5-FU (60 μg/ml) or oxaliplatin (20 μM) were used as positive controls. **(J)**, **(K)** HCT116 cells were treated with OSW-1 as in (B) or the control sulindac sulfide (120 μM) for 24 hr. Mitochondrial and cytosolic fractions were isolated from the treated cells. **(J)** Bax multimerization was analyzed by dithiobis succinimidyl propionate (DSP) cross-linking of the mitochondrial fractions followed by Western blotting under non-denaturing conditions. **(K)** Cytochrome *c* release was analyzed by Western blotting of cytochrome *c* in the mitochondrial and cytosolic fractions. Cytoplasmic α-tubulin and mitochondrial cytochrome oxidase subunit IV (Cox IV) were used as controls for loading and fractionation. Quantitative results in (A)-(F) and (H) were expressed as means ± s.d. of three or four independent experiments. *NS*, *P* >0.05; *****, *P* <0.05; ******, *P* <0.01; *******, *P* <0.001.

**Supplementary Fig. 5 OSW-1-induced degradation of OSBP and ORP4 mediates p53 K120 acetylation and PUMA upregulation. (A)** Western blotting of indicated DNA damage response markers in HCT116, RKO, Lim1215, and LoVo cells treated with OSW-1 (0.5 nM) for 24 hr. Cells treated with 5-FU (60 μg/ml) was used as a positive control. **(B)**, **(C)** Real-time RT-PCR analysis of **(B)** *Mdm2*, **(C)** *OSBP* and *ORP4* mRNA expression in HCT116 cells treated with OSW-1 (0.5 nM) at indicated time points. **(D)** Western blotting of indicated proteins in HCT116 cells transfected with empty vector, *OSBP*, *ORP4*, or both *OSBP* and *ORP4* and treated with OSW-1 as in (A). **(E)** Western blotting of Mdm2 in HCT116 cells treated with OSW-1 (0.5 nM) along with Salubrinal (5 μM) at indicated time points. **(F)** Western blotting of indicated proteins in HCT116 cells transfected with control (Ctr) or *β-TrCP* siRNA and treated with OSW-1 as in (A). Quantitative results in (B) and (C) were expressed as means ± s.d. of three or four independent experiments. *NS*, *P* >0.05.

**Supplementary Fig. 6 OSW-1-induced necroptosis involves mitochondrial calcium influx and CamKIIδ. (A)** WT, *p53*-KO, and *PUMA*-KO HCT116 cells treated with OSW-1 (0.5 nM) for 24 hr were analyzed for mitochondrial calcium by staining with the cell-permeable fluorescent Ca2^+^ indicator Rhod-2. **(A)** Analysis of Rhod-2-stained cells by confocal microscopy with mitochondrial staining by MitoTracker Green FM. *Left*, representative confocal images with arrows indicating example cells with co-localization (Scale bars, 10 μm); *right*, quantification of Rhod-2^+^ signals. **(B)** Analysis of Rhod-2-stained cells by flow cytometry. **(C)** Western blotting of indicated proteins in HCT116 cells transfected with control empty vector, *OSBP*, or *ORP4* and treated with OSW-1 as in (A). **(D)**-**(F)** HCT116 cells were treated for 24 hr with OSW-1 (0.5 nM) alone or in combination with the CamKII inhibitor KN93 (1 μM). **(D)**, **(E)** Analysis of calcium changes as in (A) and (B), respectively. **(F)** Colony formation assay was done by seeding an equal number of treated cells in 12-well plates and staining the attached cells with crystal violet after 14 days. *Left*, representative pictures of colonies; *right*, enumeration of colony numbers. **(G)** Real-time RT-PCR analysis of mRNA expression of indicated *CaMKII* isoforms (*α, β, γ,* and *δ*) in HCT116 cells. **(H)** Real-time RT-PCR analysis of *CaMKIIδ* in HCT116 cells treated with OSW-1 as in (A). **(I)-(M)** HCT116 cells transfected with control (Ctr) or *CaMKIIδ* siRNA and treated with OSW-1 as in (A) were analyzed by **(I)** crystal violet staining of viable cells, **(J)** real-time RT-PCR for the mRNA expression of *CaMKIIδ* and *CaMKIIγ*, **(K)** Western blotting of indicated proteins in whole cell lysates (WCL) and HMGB1 in 20-μl cell culture medium (M), **(L)** and **(M)** calcium changes as in (A) and (B), respectively. Quantitative results in (A), (D), (F)-(H), (J), and (L) were expressed as means ± s.d. of three or four independent experiments. In (A), (D), and (L), at least 300 cells were counted for each sample. *NS*, *P* >0.05; ******, *P* <0.01; *******, *P* <0.001.

**Supplementary Fig. 7 OSW-1 induces p53/PUMA-dependent immunogenic cell death in CRC cells. (A)** Real-time RT-PCR analysis of mRNA expression of indicated genes in WT and *PUMA-*KO HCT116 cells treated with OSW-1 (0.5 nM) for 24 hr. **(B)** Flow cytometry analysis of cell-surface calreticulin (CRT) in WT and *PUMA-*KO HCT116 cells treated with OSW-1 as in (A). *Left*, a representative flow cytometry picture; *right*, quantification of flow cytometry data. **(C)-(E)** WT, *p53* KO, and *PUMA*-KO HCT116 cells treated with OSW-1 as in (A) and DCs differentiated from healthy donors’ PBMCs were labelled with CFSE and Far Red, respectively, and co-incubated at a 1:1 ratio. **(C)** Analysis of DC phagocytosis by fluorescence microscopy using green (for CFSE) and magenta (for Far Red) channels (Scale bars, 20 μm). **(D)**, **(E)** Analysis of DC phagocytosis by flow cytometry. **(D)** Representative flow cytometry pictures. **(E)** Quantification of flow cytometry data. Quantitative results in (A), (B), and (E) are expressed as means ± s.d. from three or four independent experiments. *, *P*<0.05; **, *P*<0.01; ***, *P*<0.001.

**Supplementary Fig. 8 The *in vivo* therapeutic effects of OSW-1 involve p53/PUMA-mediated antitumor immune response. (A)-(C)** C57BL/6 mice were injected s.c. with 5×10^5^ WT, *p53*-KO, or *PUMA*-KO MC38 cells. After tumor growth for 7 days, mice were treated with OSW-1 (i.p.; 12.5 μg/kg) as indicated in Fig. 6A. **(A)** Volumes of individual tumors at indicated time points (n=9-10 in each group). **(B)** Survival of the treated mice. **(C)** Body weight of the treated mice. **(D)**, **(E)** Paraffin-embedded tumor tissues from mice treated as in (A) and resected at day 11 were analyzed by immunostaining for **(D)** CD3 and **(E)** CD11c. *Left*, representative staining pictures with arrows indicating example cells with positive staining (Scale bars, 20 μm); *right*, quantification of CD3^+^ and CD11c^+^ cells (n=5 in each group). At least 300 nuclei from 3 randomly selected fields were counted for each tumor. **(F)**, **(G)** Real-time RT-PCR analysis of mRNA expression of indicated immune markers in OSW-1-treated **(F)** WT and *PUMA*-KO MC38 tumors and **(G)** spleens of tumor-bearing mice. Pooled mRNA samples from 3 randomly selected tissues in each group were analyzed. Results were expressed as means ± s.d. of three independent experiments. ******, *P* <0.01; *******, *P* <0.001.

**Supplementary Fig. 9 The *in vivo* antitumor activity of OSW-1 is mediated by MLKL-dependent necroptosis.** C57BL/6 mice were injected s.c. with 5×10^5^ WT or *MLKL*-KO MC38 cells. After tumor growth for 7 days, mice were treated with OSW-1 (i.p.; 12.5 μg/kg) as indicated in Fig. 6F. **(A)** Volumes of individual tumors at indicated time points. **(B)** Body weight of treated mice. **(C)** Paraffin-embedded tumor tissues from mice treated as in Fig. 6F and resected at day 11 were analyzed by immunostaining for HMGB1 (n=3 in each group). *Left*, representative staining pictures with arrows indicating example cells with extranuclear HMGB1 staining and hollow nuclei (scale bars, 20 μm); *right*, quantification of cells with nuclear HMGB1 staining. At least 300 nuclei from 3 randomly selected fields were counted for each tumor. *******, *P* <0.001.

**Supplementary Fig. 10 Nanoparticle packaging improves the efficacy of OSW-1**. **(A)-(C)** C57BL/6 mice were injected with 5×10^5^ WT MC38 cells. After tumor growth for 7 days, mice were treated with OSW-1 packaged in nanoparticles (Nano-OSW-1; i.p.; 5 μg/kg) as indicated in (A) (n=6 in each group). **(A)** Average tumor volume at indicated time points. **(B)** Volumes of individual tumors at indicated time points. **(C)** Body weight of the treated mice. **(D)**, **(E)** BALB/c mice were injected with 1×10^6^ WT CT26 cells. After tumor growth for 7 days, mice were treated with Nano-OSW-1 at indicated route, dose, and frequency as in (D) (n=6 in each group). **(E)** Average tumor volume at indicated time points. **(F)** Body weight of the treated mice. **, *P* < 0.01; ***, *P* < 0.001.

**Supplementary Fig. 11 OSW-1 combined with anti-PD-1 antibody enhances tumor suppression and antitumor immune response.** C57BL/6 mice bearing WT MC38 tumors were treated with OSW-1 (i.p.; 5 μg/kg), anti-mouse-PD-1 (i,p.; 150 μg/dose), or their combination as indicated in Fig. 7A. **(A)** Volumes of individual tumors at indicated time points (n=11-12 in each group). **(B)** Survival of the treated mice. **(C)** Body weight of the treated mice. **(D)-(F)** Paraffin-embedded tumor tissues resected on day 11 were analyzed by immunostaining for **(D)** CD3+, **(E)** CD8+, and **(F)** CD11c+ cells. *Left*, representative staining pictures with arrows indicating example cells with positive staining (Scale bars, 20 μm); *right*, quantification of positive cells (n=5 in each group). At least 300 nuclei from 3 randomly selected fields were counted for each tumor. **(G)** Flow cytometry analysis of infiltrating CD4+ T cells in the resected tumors (n=6 in each group). *NS*, *P* >0.05; *, *P* <0.05; **, *P*<0.01*,* *******, *P* <0.001.

**Supplementary Fig. 12 The enhanced therapeutic and immunogenic effects of OSW-1 combined with anti-PD-1 antibody are dependent on p53 and PUMA.** C57BL/6 mice bearing WT, *p53*-KO, or *PUMA*-KO MC38 tumors were treated with OSW-1 (i.p.; 5 μg/kg), anti-mouse-PD-1 (i,p.; 150 μg/dose), or their combination as indicated in Fig. 7G. **(A)** Volumes of individual tumors at indicated time points (n=7-12 in each group). **(B)** Survival of the treated mice. **(C)** Body weight of the treated mice. **(D)-(F)** Paraffin-embedded tumor tissues resected on day 11 were analyzed by immunostaining for **(D)** CD3+, **(E)** CD8+, and **(F)** CD11c+ cells. *Left*, representative staining pictures with arrows indicating example cells with positive staining (Scale bars, 20 μm); *right*, quantification of positive cells (n=3 in each group). At least 300 nuclei from 3 randomly selected fields were counted for each tumor. ***, *P*<0.001.

**Supplementary Fig. 13 The *in vivo* therapeutic effects of OSW-1 require antitumor immune response. (A)**, **(B)** C57BL/6 mice were injected s.c. with 5×10^5^ WT MC38 cells. After tumor growth for 5 or 7 days, mice were treated with OSW-1 (i.p.; 12.5 μg/kg), anti-CD8a (i,p.; 150 μg/dose) or their combination as indicated in (A) (n=7-8 in each group). **(A)** Average tumor volume at indicated time points. **(B)** Volumes of individual tumors at indicated time points. **(C)**, **(D)** NSG mice implanted with 5×10^5^ WT MC38 cells were treated with OSW-1 (i.p.; 12.5 μg/kg) as indicated in (C) (n=6 in each group). **(C)** Average tumor volume at indicated time points. **(C)** Volumes of individual tumors at indicated time points. **(E)**, **(F)** NSG mice were injected with 5×10^5^ WT MC38 cells. After tumor growth for 7 days, mice were treated with OSW-1 (i.p.; 5 μg/kg), anti-mouse-PD-1 (i,p.; 150 μg/dose), or their combination as indicated in (E) (n=6 in each group). **(E)** Average tumor volume at indicated time points. **(F)** Volumes of individual tumors at indicated time points. ***, *P*<0.001.

**Supplementary Fig. 14 A model depicting the mechanism of OSW-1-induced and RIP1/RIP3-independent necroptosis and tumor suppression.** OSW-1 treatment induces β-TrCP to promote Mdm2 degradation and p53 stabilization. OSW-1 treatment also degrades OSBP and ORP4 to promote ER stress and p53 K120 acetylation. As a result, PUMA is selectively induced by p53-mediated transcriptional activation. However, other p53 downstream apoptosis regulators such as Noxa, Bax, Bid and Bim are not induced. Therefore, apoptosis pathways cannot be turned on. A high level of PUMA favors induction of necroptosis by causing mitochondrial calcium influx to activate CamKIIδ, which binds to and phosphorylates MLKL to trigger necroptotic death in a RIP1/RIP3-independent manner. Cells undergoing necroptosis release DAMPs such as HMGB1, which activates immune cells and reshapes the tumor immune microenvironment, leading to sensitization to anti-PD-1 antibody.
