# Supplementary material for: Depletion of oxysterol-binding proteins by OSW-1 triggers RIP1/RIP3-independent necroptosis and sensitization to cancer immunotherapy
Source: Cell Death Differ. 2025 May 6;32(11):2038–52. doi: 10.1038/s41418-025-01521-8 (PMC12572256; doi:10.1038/s41418-025-01521-8)

Supplementary Fig. 1

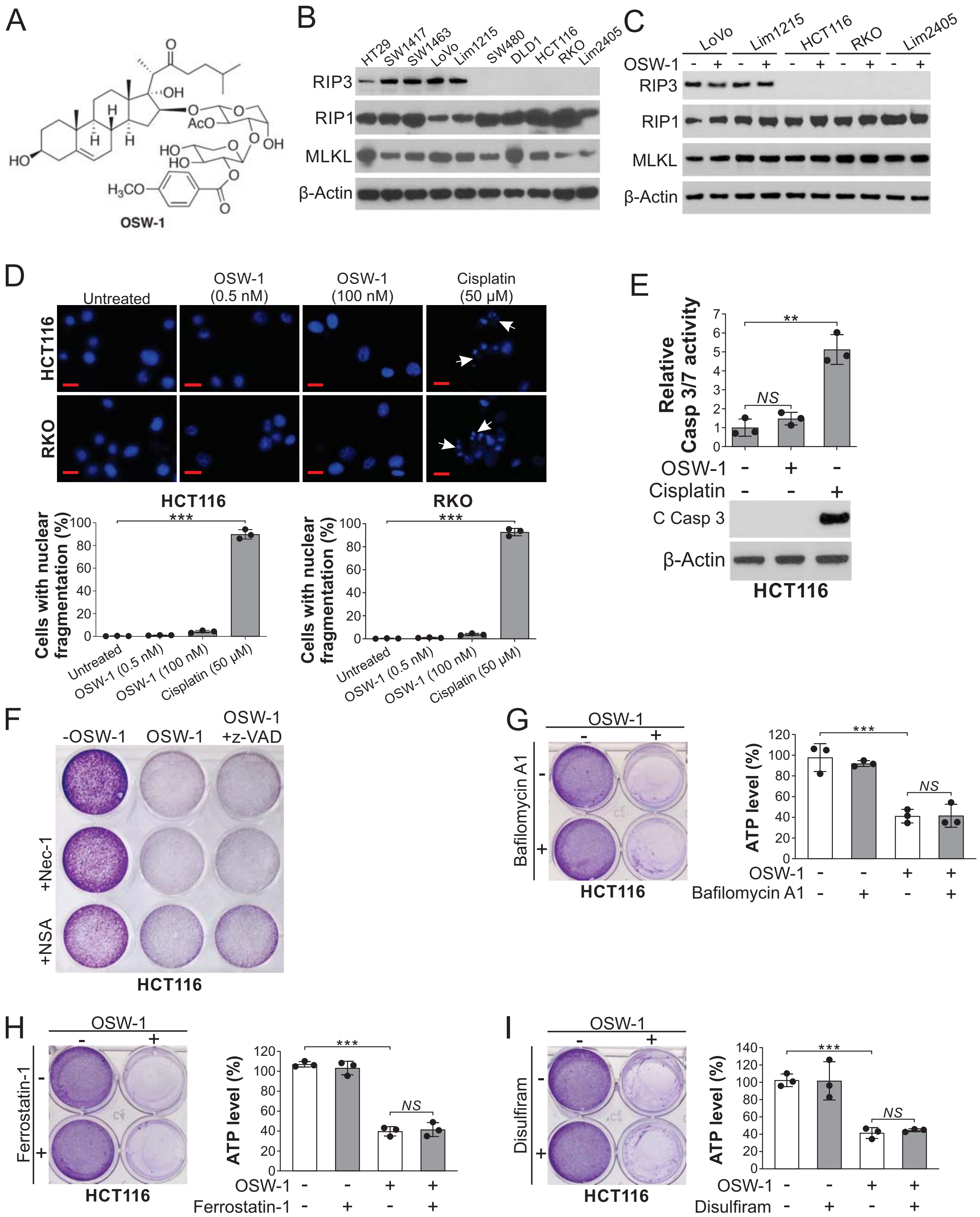

Supplementary Fig. 2

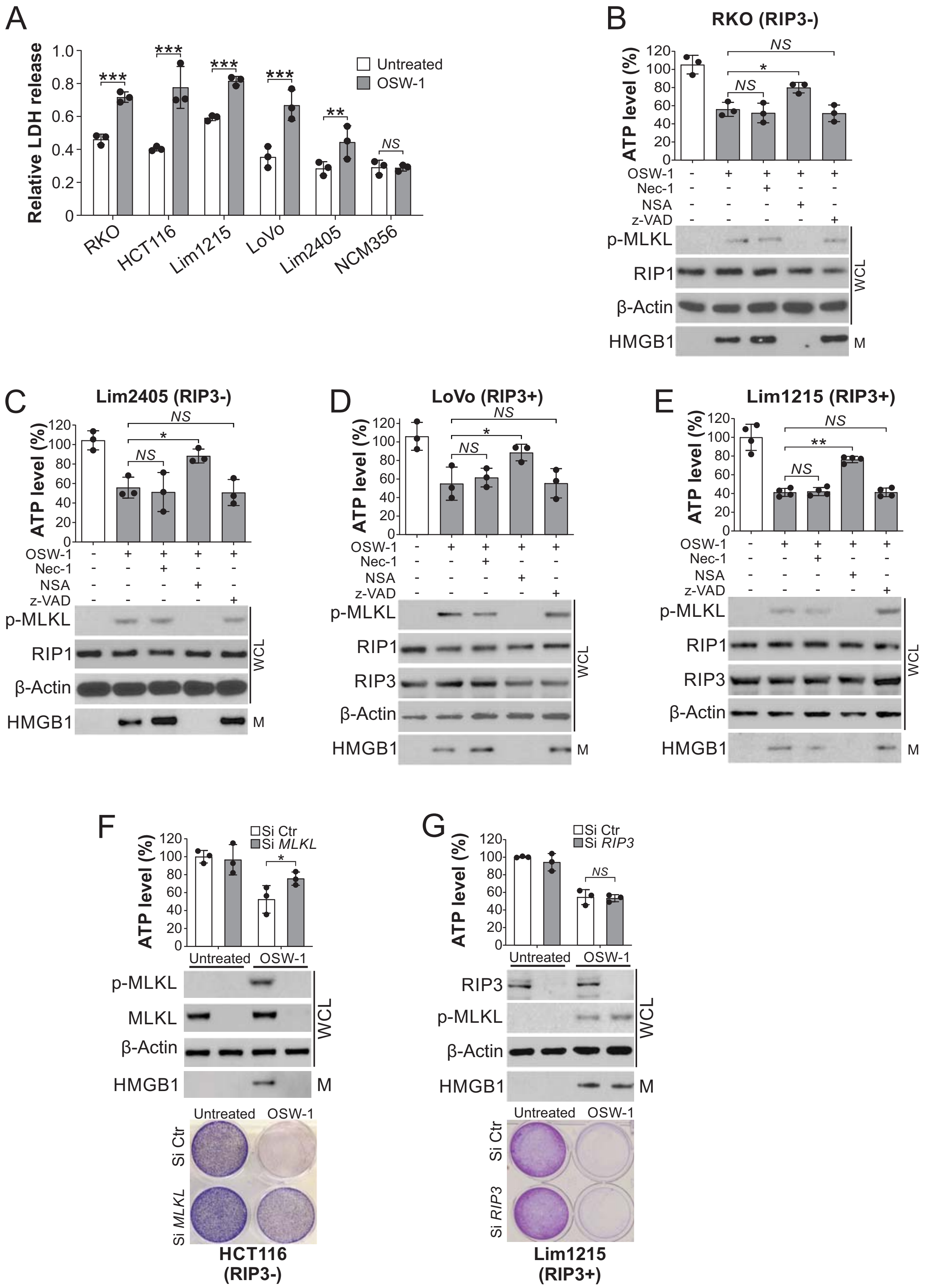

Supplementary Fig. 3

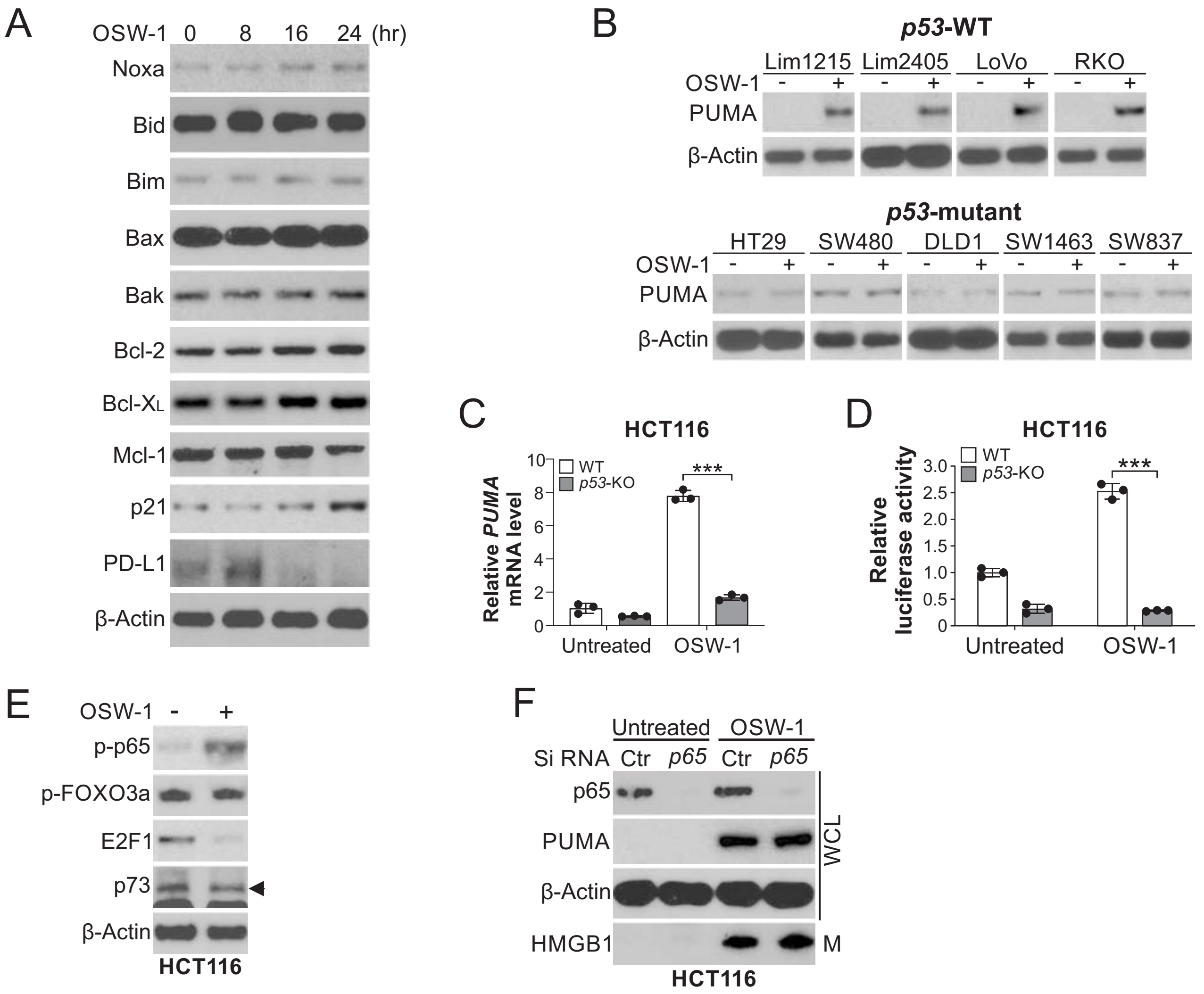

Supplementary Fig. 4

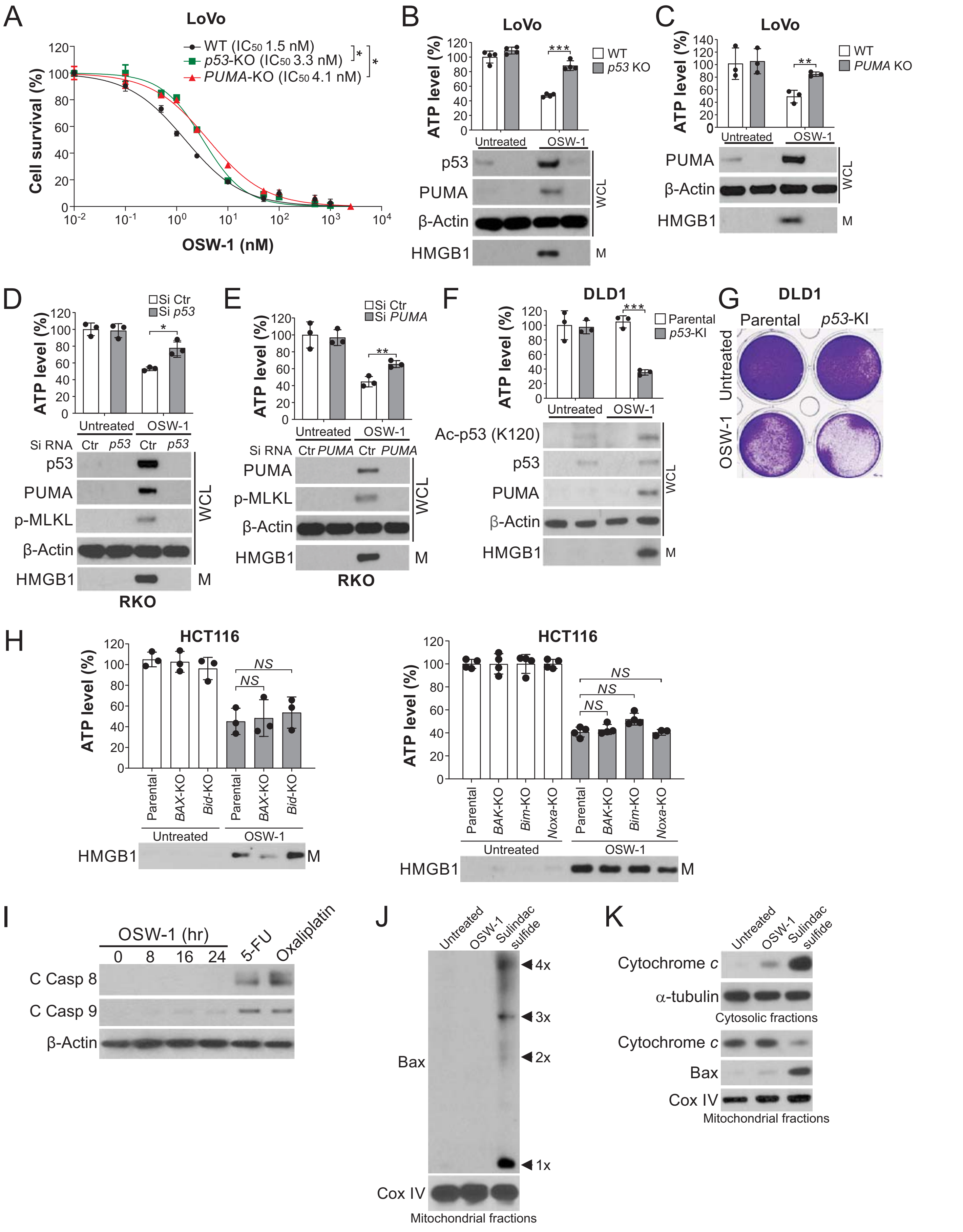

Supplementary Fig. 5

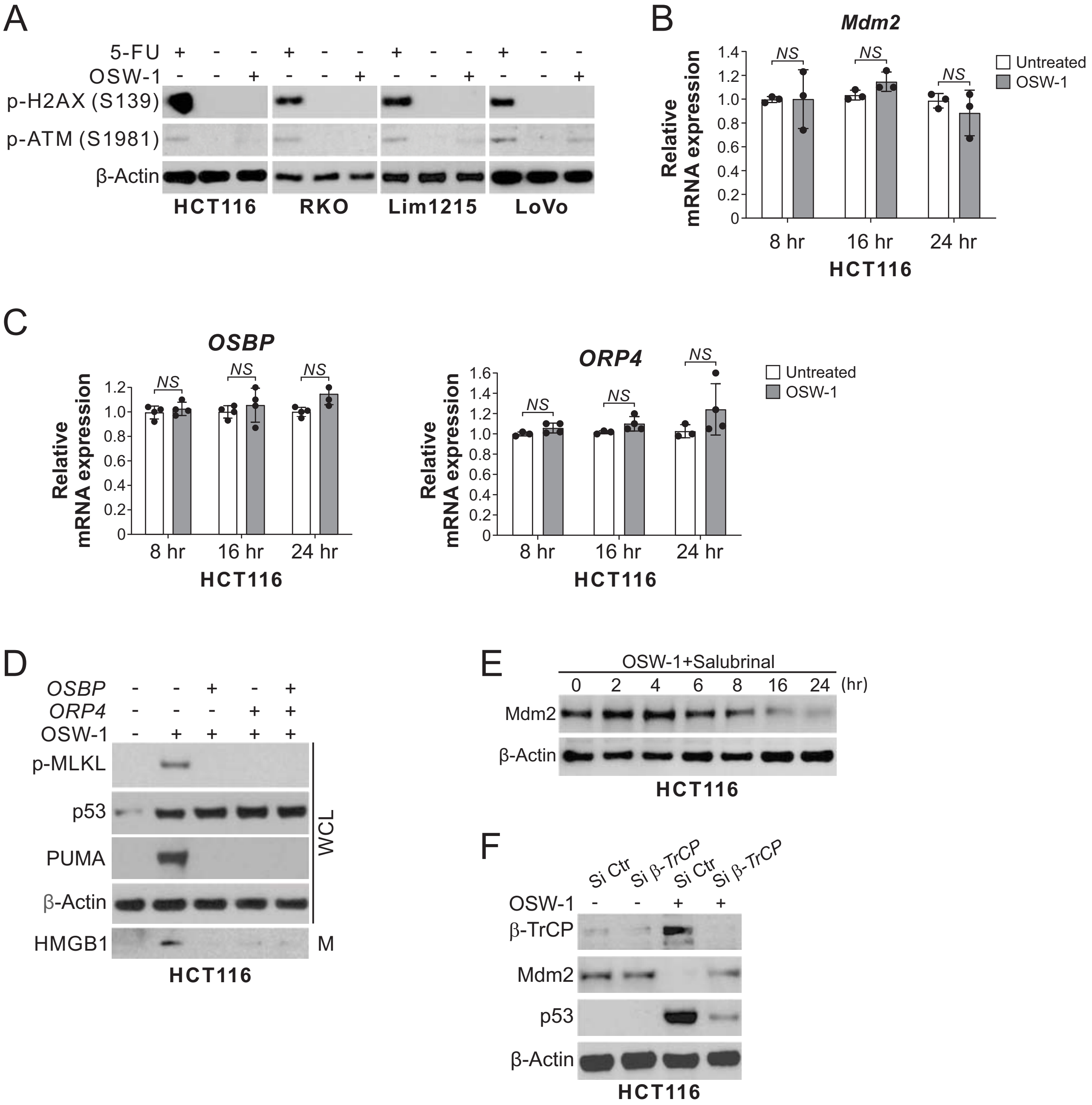

Supplementary Fig. 6

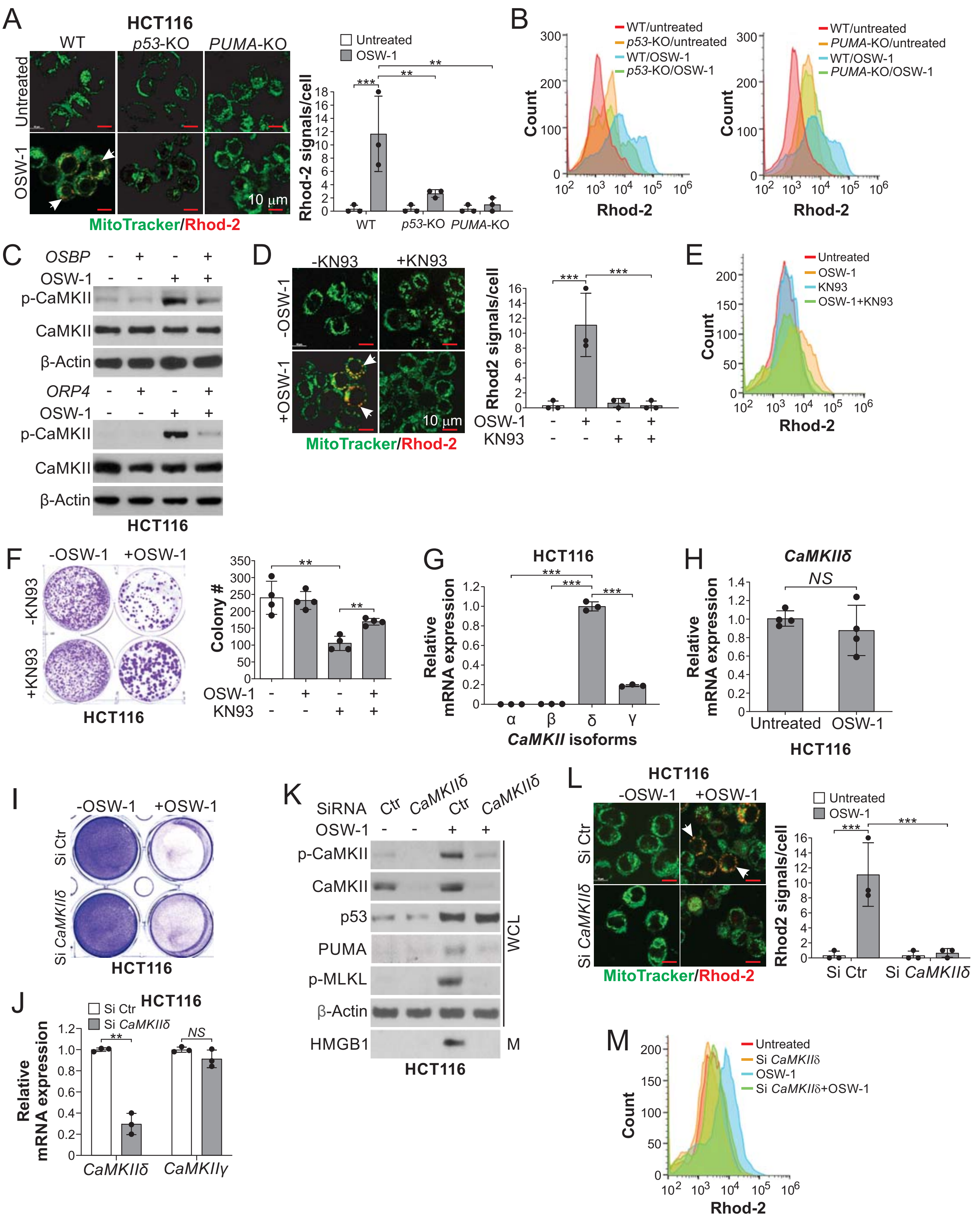

Supplementary Fig. 7

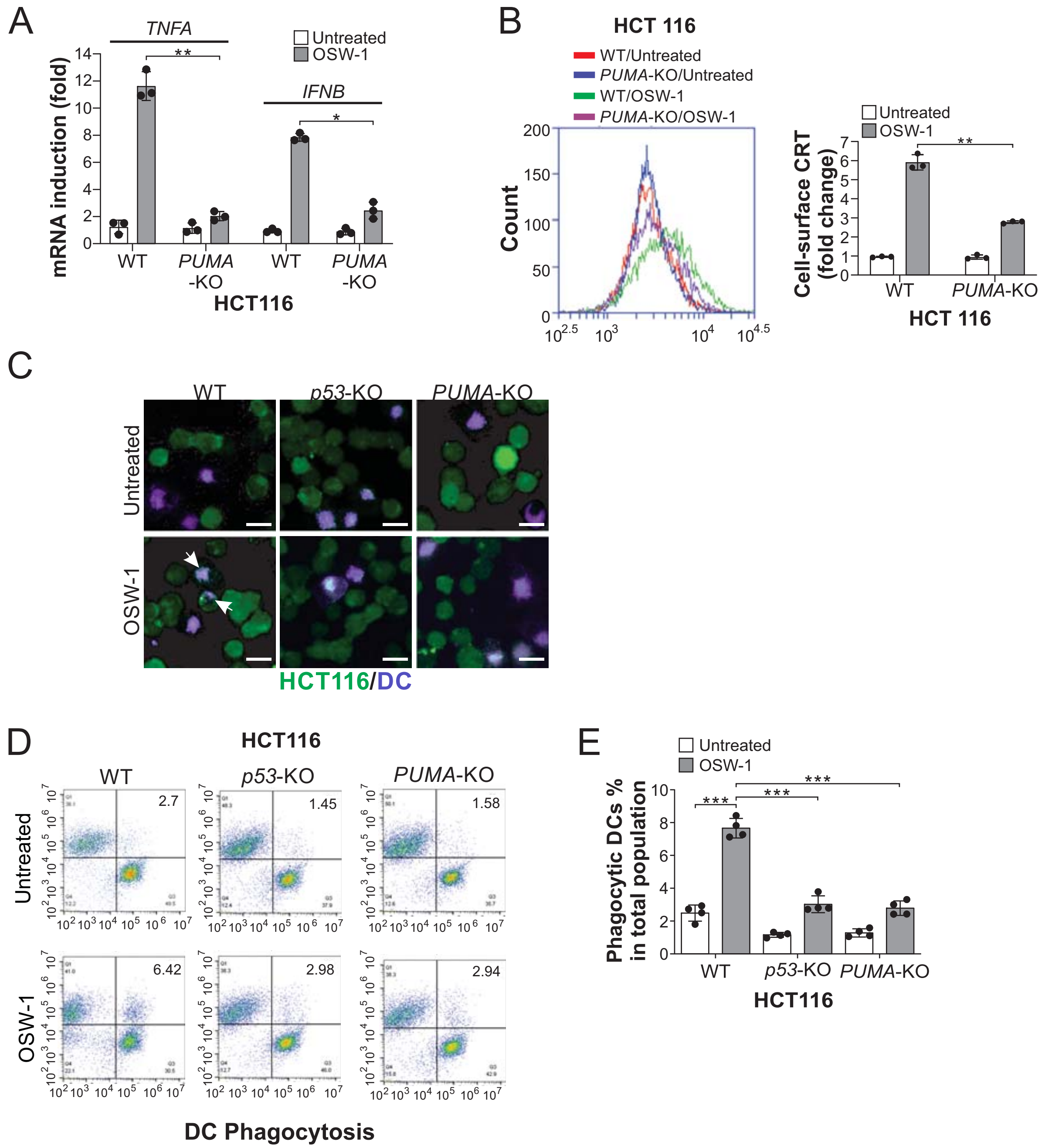

Supplementary Fig. 8

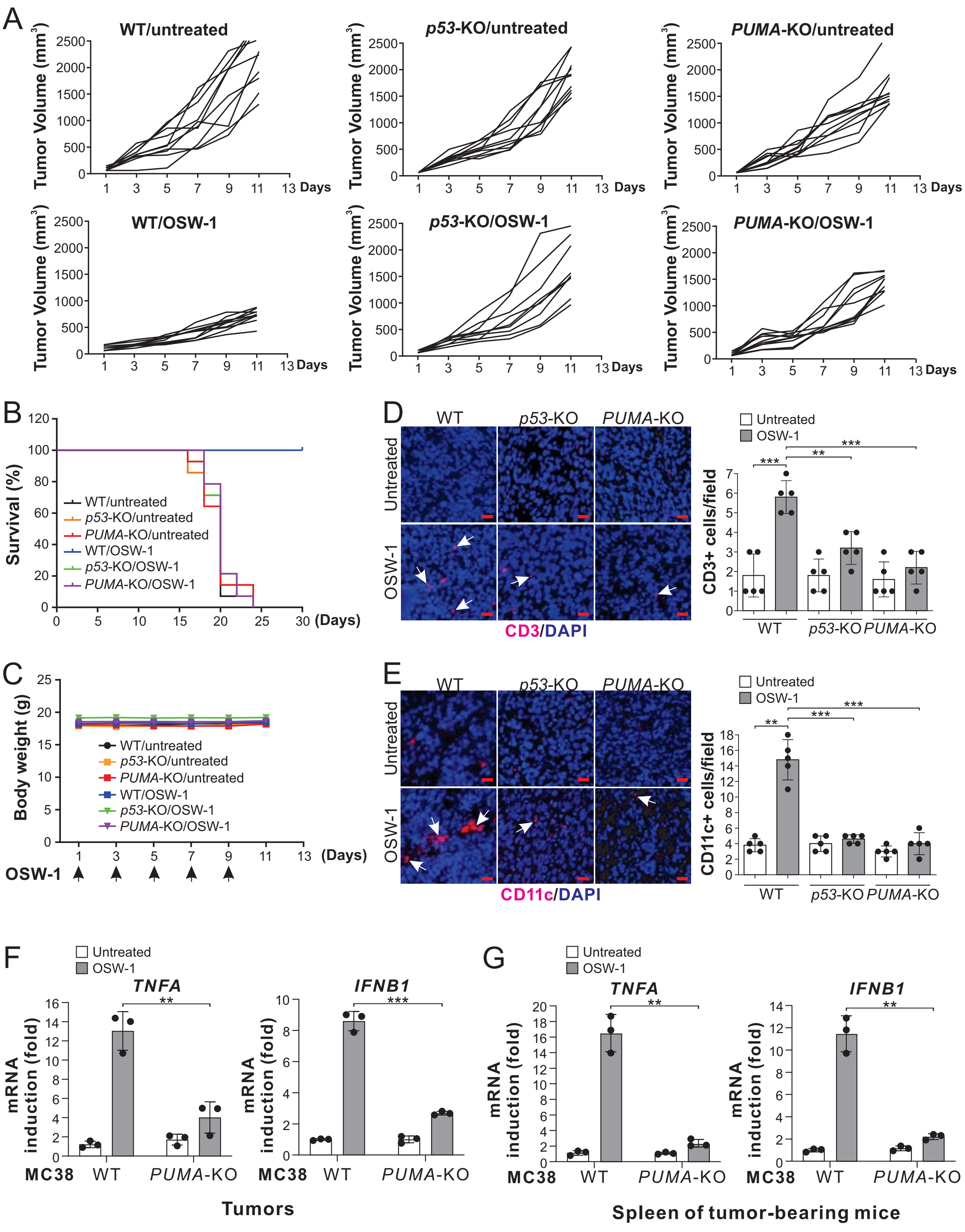

Supplementary Fig. 9

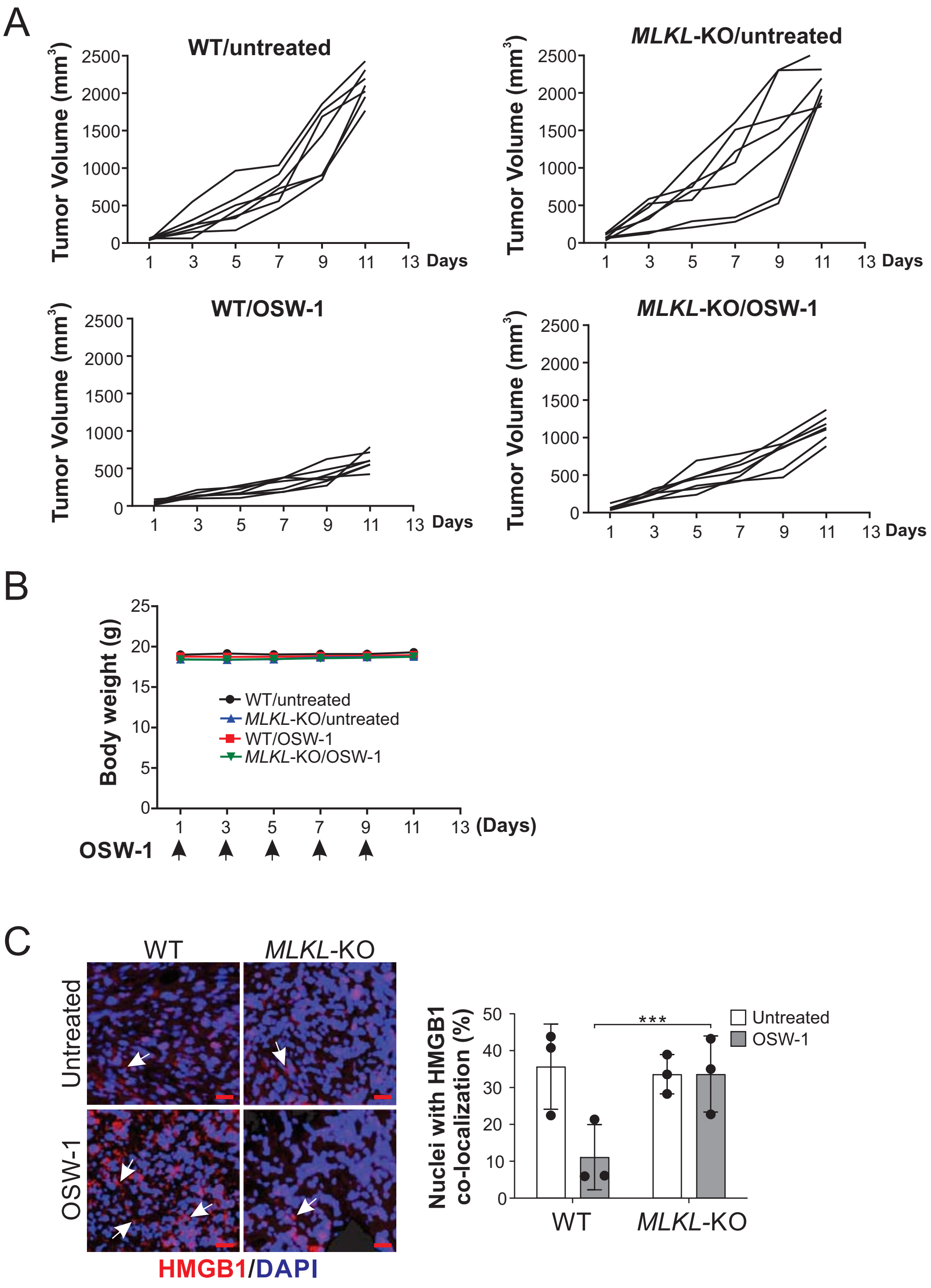

Supplementary Fig. 10

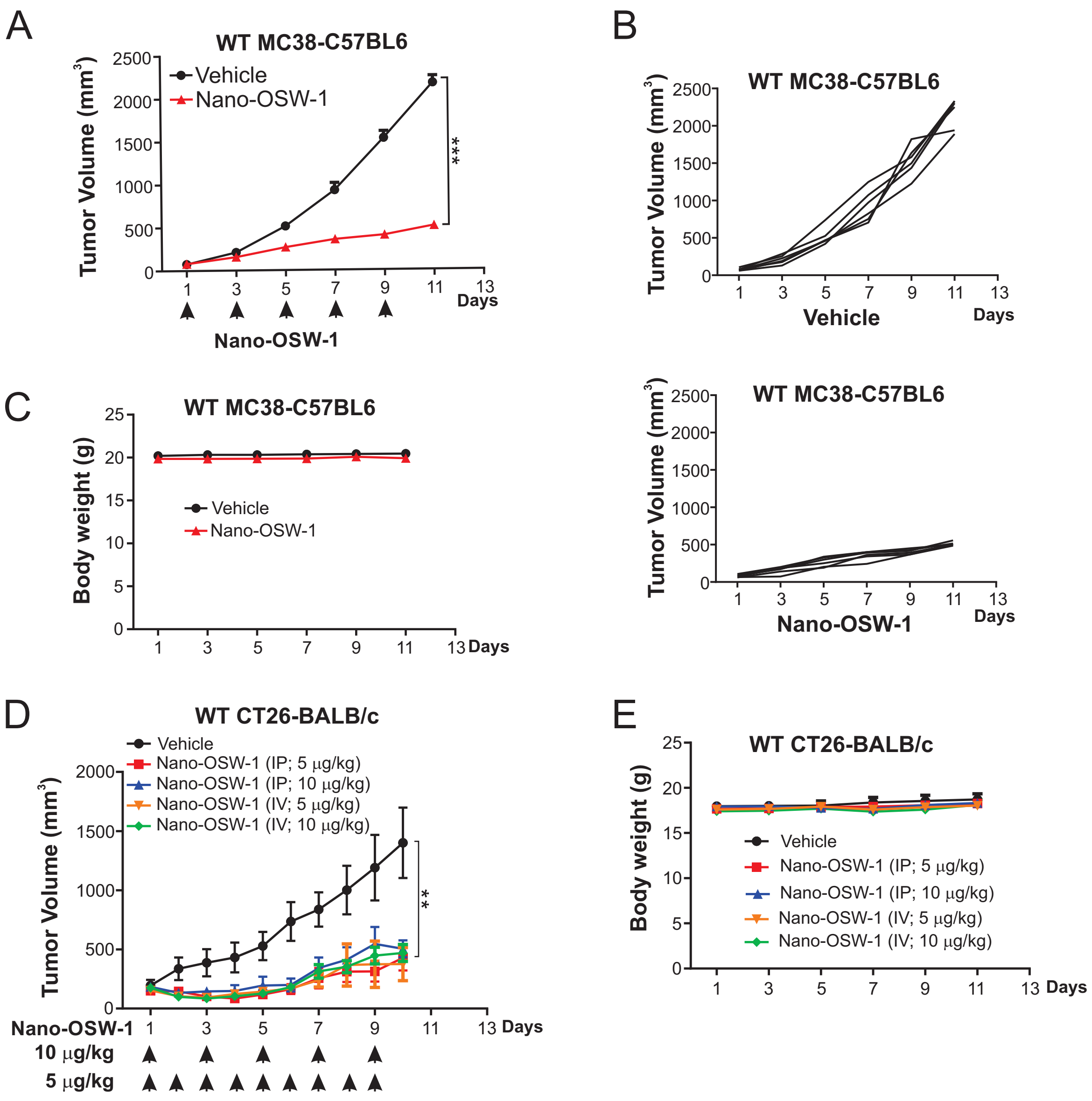

Supplementary Fig. 11

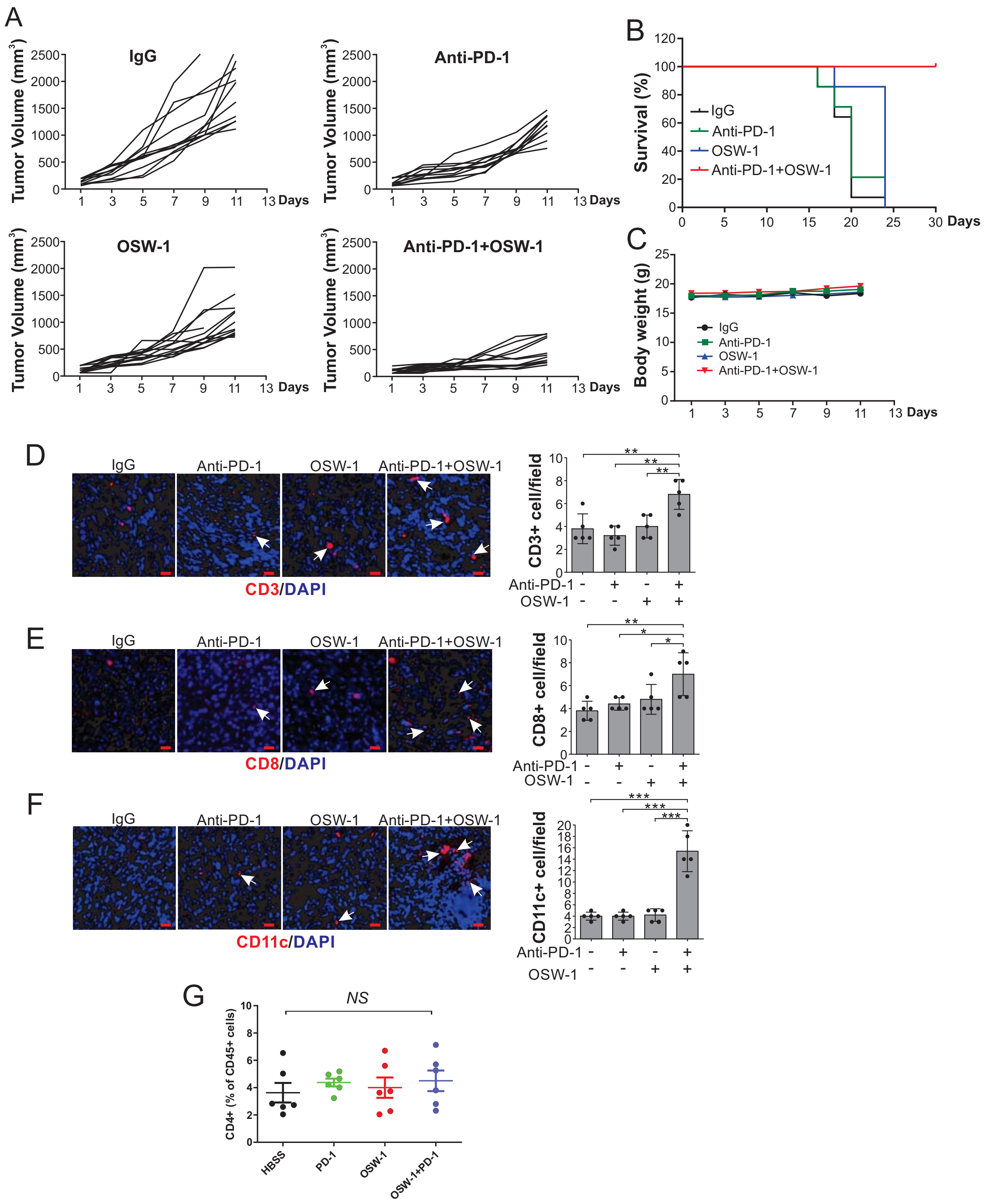

Supplementary Fig. 12

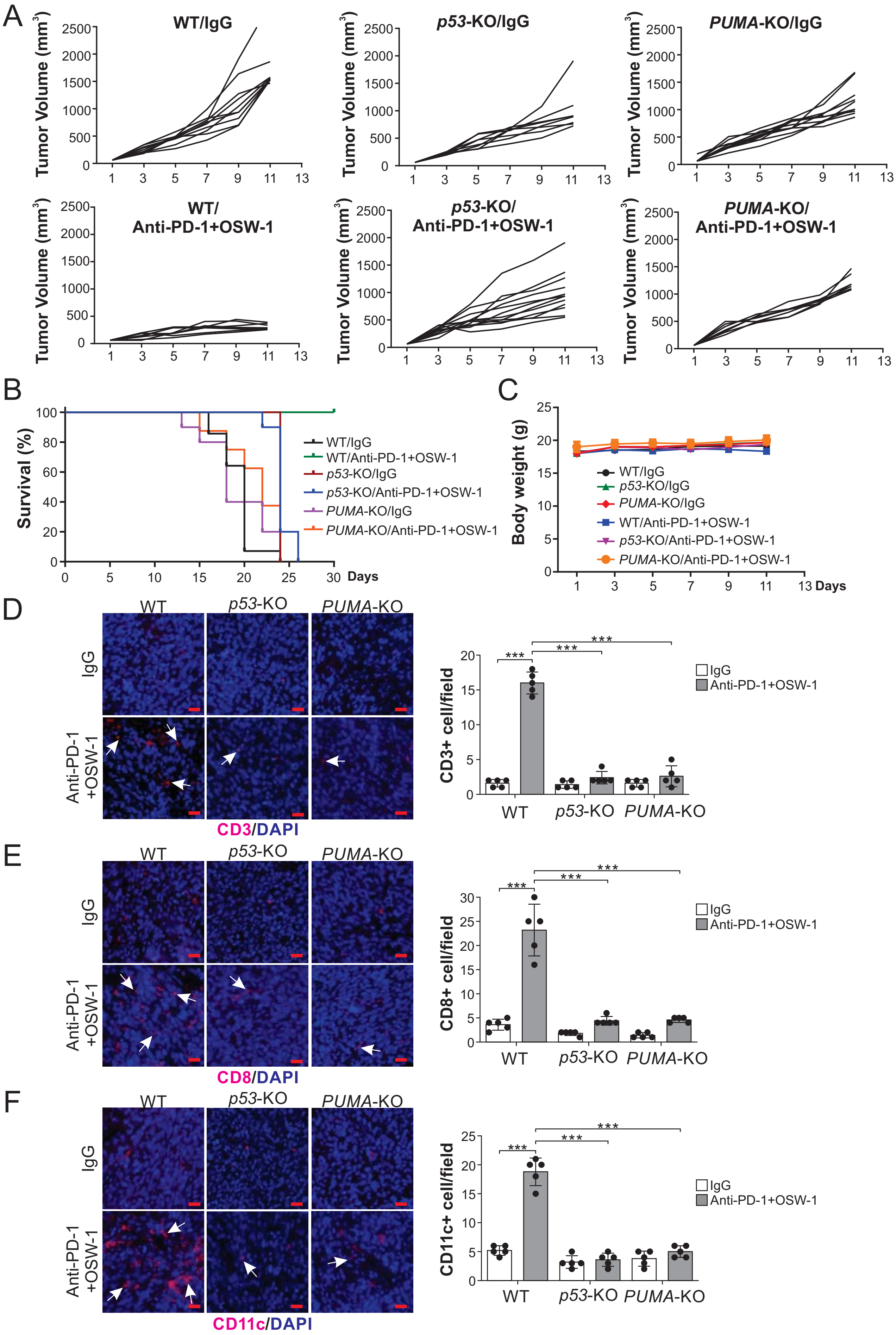

Supplementary Fig. 13

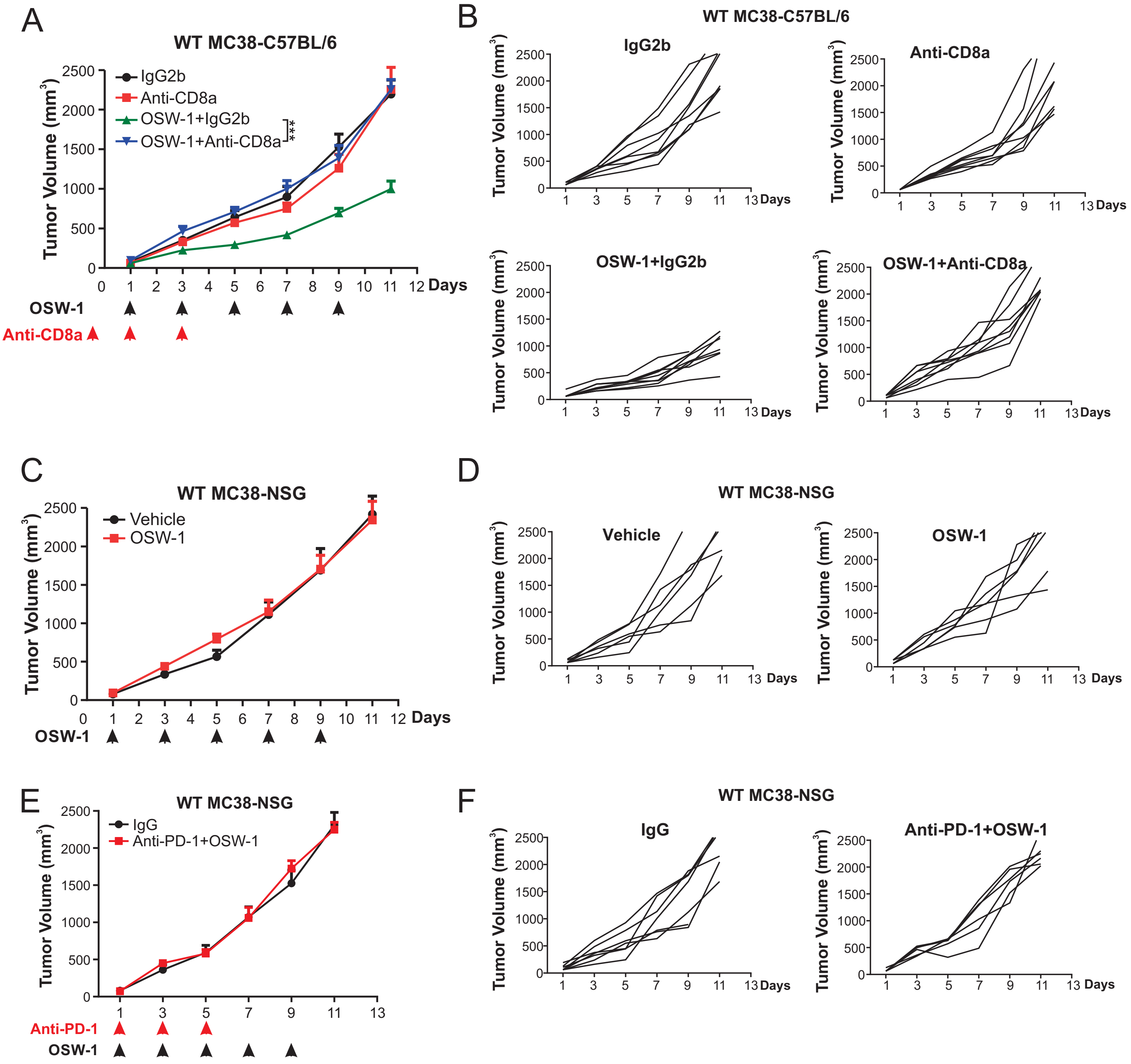

Supplementary Fig. 14

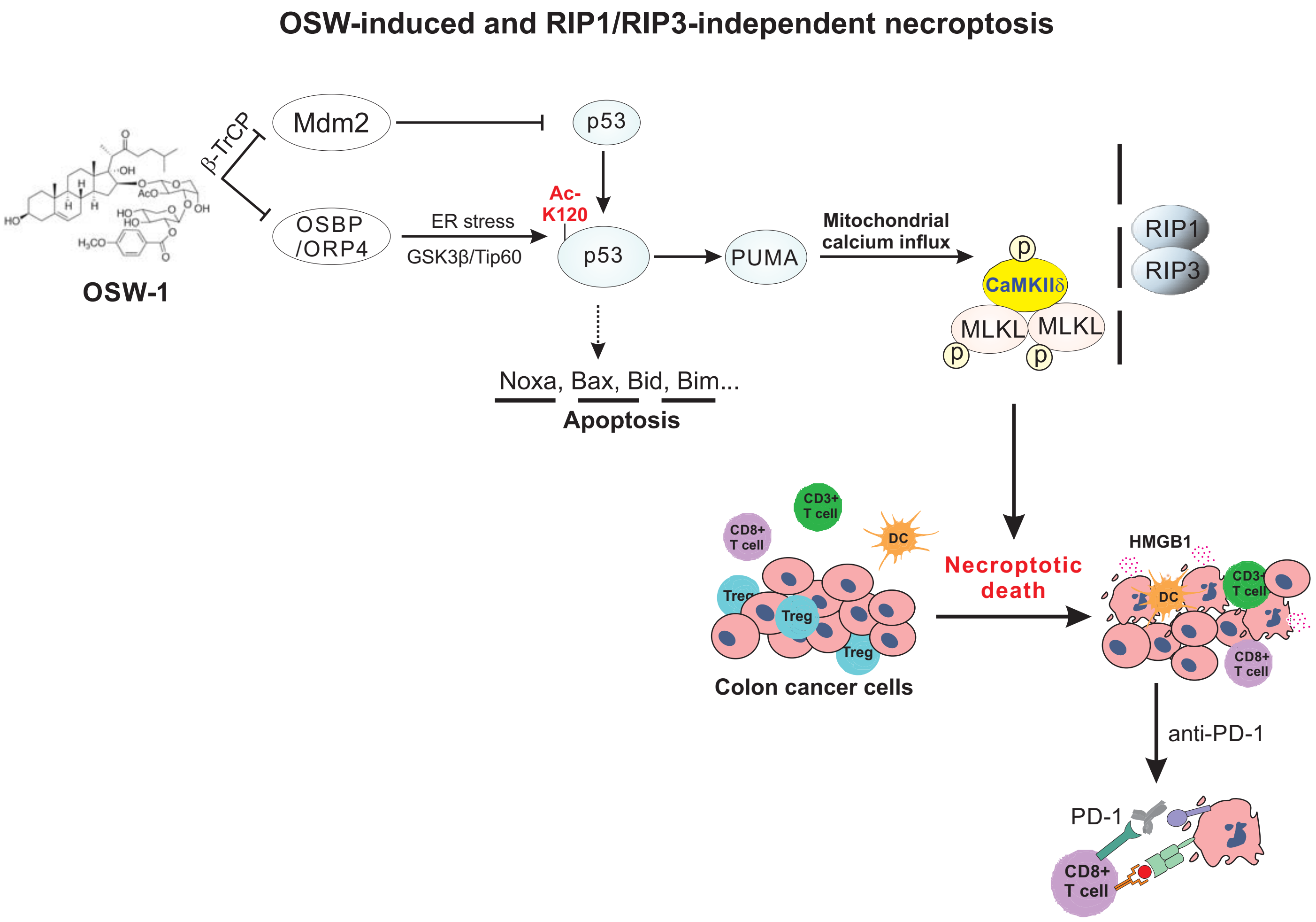

Supplement: Supplementary file 2 — Supplementary Figure 1-14 [file 41418_2025_1521_MOESM2_ESM.pdf]
