# Supplementary material for: Depletion of oxysterol-binding proteins by OSW-1 triggers RIP1/RIP3-independent necroptosis and sensitization to cancer immunotherapy
Source: Cell Death Differ. 2025 May 6;32(11):2038–52. doi: 10.1038/s41418-025-01521-8 (PMC12572256; doi:10.1038/s41418-025-01521-8)
Supplement: Supplementary file 3 — Supplementary Table 1 [file 41418_2025_1521_MOESM3_ESM.docx]

| **Supplementary Table 1. List of cell lines and reagents** | | |
| --- | --- | --- |
| **Cell lines** | **Source/reference** | **Catalogue #** |
| HCT116 | ATCC | #CCL-247 |
| *p53-*KO HCT116 | Gift from Dr. Bert Vogelstein | N/A |
| *PUMA-*KO HCT116 | Gift from Dr. Bert Vogelstein | N/A |
| *BAX*-KO HCT116 | Gift from Dr. Bert Vogelstein | N/A |
| *Bid*-KO HCT116 | Leibowitz et al *PNAS* 2014; PMID: 25368155 | N/A |
| *Bak*-KO HCT116 | Gift from Dr. Bert Vogelstein | N/A |
| *Bim*-KO HCT116 | Tong et al *Cancer Res* 2018; PMID: 29895675 | N/A |
| *Noxa*-KO HCT116 | Tong et al *Oncotarget* 2014; PMID: 25237903 | N/A |
| Lim1215 | Horizon Discovery | #HD PAR-108 |
| Lim2405 | Gift from Dr. Alberto Bardelli | N/A |
| HT29 | ATCC | #HTB-38 |
| DLD1 | ATCC | #CCL-221 |
| WT-*p53*-KI DLD1 | Gift from Dr. Bert Vogelstein | N/A |
| LoVo | ATCC | #CCL-229 |
| *p53-*KO LoVo | Chen et al *Mol Cancer Ther* 2024; PMID: 37992761 | N/A |
| RKO | ATCC | #CRL-2577 |
| SW480 | ATCC | #CCL-228 |
| SW1463 | ATCC | #CCL-234 |
| SW837 | ATCC | #CCL-235 |
| NCM356 | INCELL | NCM356D |
| MC38 | Gift from Dr. David L. Bartlett (originally from NCI) | N/A |
| *p53-*KO MC38 | Chen et al *Mol Cancer Ther* 2024; PMID: 37992761 | N/A |
| *PUMA-*KO MC38 | Chen et al *Mol Cancer Ther* 2024; PMID: 37992761 | N/A |
|  |  |  |
| **Constructs** | **Source/reference** | **Catalogue #** |
| V5-WT-p53 | Hao el al *PNAS* 2022; PMID: 36508676 | N/A |
| pcDNA3.1-HA | Addgene | #128034 |
| pGLS3-5xHRE-p53(K120R) | Addgene | #72554 |
| pGLS3-5xHRE-p53(K164R) | Addgene | #72555 |
| pcDNA-p53(K120R) | This study | N/A |
| pcDNA-p53(K164R) | This study | N/A |
| pcDNA3.1-V5 | ThermoFisher | #V81020 |
| pLJM1-FLAG-GFP-OSBP | Addgene | #134659 |
| MGC Human OSBP2 Sequence-Verified cDNA | Horizon Discovery | #MHS6278-211690367 |
| pcDNA-OSBP | This study | N/A |
| pcDNA-ORP4 | This study | N/A |
| pBV-Luc-Frag A | Yu el al *Mol Cell* 2001; PMID:11463391 | N/A |
| Ad-ΔBH3 | Yu el al *Mol Cell* 2001; PMID: 11463391 | N/A |
| Ad-PUMA | Yu el al *Mol Cell* 2001;PMID: 11463391 | N/A |
| pSpCas9-2A-GFP | Addgene | #48138 |
| pSpCas9-2A-GFP-*hMLKL* | This study | N/A |
| pSpCas9-2A-GFP-*hCaMKIIδ* | This study | N/A |
| pSpCas9-2A-GFP-*mMLKL* | This study | N/A |
|  | | |
| **Chemicals** | **Source** | **Catalogue #** |
| DMSO | Sigma-Aldrich | #D2650 |
| Crystal violet | Sigma-Aldrich | #C0775 |
| Hoechst 33258 | ThermoFisher | #H1398 |
| z-VAD-fmk | ApexBio | #A1920 |
| Necrostatin (Nec-1) | ApexBio | #50-101-0399 |
| Necrosulfonamide (NSA) | Caymen Chemical | #17729 |
| KN-93 | MedChemExpress | #HY-15465 |
| OSW-1 | Caymen Chemical | #30310 |
| OSW-1 | Gift from Dr. Zhendong Jin | N/A |
| Cycloheximide | Sellleckchem | #S7418 |
| DAPI | Vector Laboratories | #H-1200 |
| CHIR99021 | LC Laboratories | #C-6556 |
| 5-Fluorouracil | Sigma-Aldrich | #F6627 |
| Cisplatin | Sigma-Aldrich | #15663-27-1 |
| Oxaliplatin | LC Laboratories | #O-7111 |
| CFSE | ThermoFisher | #C34554 |
| Far Red | ThermoFisher | #C34564 |
| Salubrinal | ApexBio | #B2025 |
| Dithiobis succinimidylpropionate (DSP) | ThermoFisher | #A35393 |
|  | | |
| **Antibodies** | **Source** | **Catalogue #** |
| ***Western Blotting*** |  |  |
| Rabbit polyclonal, PUMA | Abcam | #ab9643 |
| Mouse monoclonal, cleaved caspase 8 | Cell Signaling Technology | #9746 |
| Rabbit polyclonal, cleaved caspase 3 | Cell Signaling Technology | #9661 |
| Mouse monoclonal, p53 | Santa Cruz Biotechnology | #sc-126 |
| Rabbit polyclonal, p53 | Aviva Systems Biology | ARP37897-P050 |
| Mouse monoclonal, β-Actin | Sigma-Aldrich | #A5441 |
| Mouse monoclonal, p21 | EMD Millipore | #OP64 |
| Rabbit monoclonal, phospho--ATM (S1981) | Abcam | #ab81292 |
| Mouse monoclonal, p53 (K120) | Abcam | #ab78316 |
| Rabbit polyclonal, Tip60(S86) | Abcam | #ab73207 |
| Rabbit Polyclonal Phospho-GSK3β (S9) | Cell Signaling Technology | #9336 |
| Rabbit monoclonal, GSK3β | Cell Signaling Technology | #9315 |
| Mouse monoclonal, cytochrome *c* | BD Biosciences | #556433 |
| Mouse monoclonal, V5 | ThermoFisher | #R960-25 |
| Rabbit polyclonal, p73 | Bethyl Laboratories | #A300-126A |
| Rabbit polyclonal,  Phospho-NF-κB p65 (S536) | Cell Signaling Technology | #3033 |
| Rabbit polyclonal, HMGB1 | Abcam | #Ab18256 |
| Rabbit monoclonal, CaMKII | Cell Signaling Technology | #4436 |
| Rabbit monoclonal,  Phospho-CaMKII (Thr286) | Cell Signaling Technology | #12716 |
| Mouse monoclonal, OSBP | Santa Cruz Biotechnology | #sc-365771 |
| Mouse monoclonal, ORP4 | Santa Cruz Biotechnology | #sc-365922 |
| Rabbit polyclonal, p65 | Santa Cruz Biotechnology | #sc-109 |
| Rabbit monoclonal,  Phospho-eIF2α (Ser51) | Cell Signaling Technology | #3398 |
| Rabbit monoclonal, GRP78/BiP | Cell Signaling Technology | #3177 |
| Mouse monoclonal, Bcl-2 | DAKO | #M0887 |
| Mouse monoclonal, Mcl-1 | BD Biosciences | #559027 |
| Rabbit monoclonal, human phospho-MLKL (S358) | Abcam | #Ab187091 |
| Rabbit monoclonal, mouse phospho-MLKL (S345) | Abcam | #Ab196436 |
| Rabbit polyclonal, MLKL | Abgent | AP-14272b |
| Rabbit polyclonal, MLKL | Sigma-Aldrich | MABC604 |
| Rabbit polyclonal, pChk2 (Thr68) | Novus Biologicals | NB100-92502 |
| Rabbit polyclonal, Chk2 | Santa Cruz Biotechnology | #sc-9064 |
| Mouse Monoclonal, γ-H2AX  (Ser139) | EMD Millipore | #05-636 |
| Mouse Monoclonal, Noxa | EMD Millipore | #OP180 |
| Mouse monoclonal, BAX | BD Biosciences | #610983 |
| Rabbit polyclonal, Bim | Cell Signaling Technology | #2819 |
| Rabbit polyclonal, BAK | Sigma-Aldrich | #06-536 |
| Rabbit polyclonal, Bid | Cell Signaling Technology | #2002 |
| Mouse monoclonal, Mdm2 | Santa Cruz Biotechnology | #sc-965 |
| Rabbit polyclonal, Phospho-FoxO1 (T24)/FoxO3a (T32) | Cell Signaling Technology | #9464 |
| Mouse monoclonal, E2F1 | Santa Cruz Biotechnology | #sc-251 |
| Mouse monoclonal, RIP1 | BD Biosciences | #610458 |
| Rabbit polyclonal, RIP3 | Abcam | #ab72106 |
| Mouse monoclonal, phospho-ATM (S1981) | Santa Cruz Biotechnology | #47739 |
| Rabbit monoclonal, PD-L1 | Cell Signaling Technology | #13684S |
| Rabbit polyclonal, cytochrome *c* | BD Biosciences | # BDB556433 |
| Mouse monoclonal, COX IV | ThermoFisher | #A21348 |
| Mouse monoclonal, α-tubulin | EMD Millipore | #CP06 |
| Mouse monoclonal, β-TrCP1 | ThermoFisher | # 37-3400 |
|  | | |
| ***Animal Treatment*** |  |  |
| *InVivoPure* Dilution Buffer | BioXCell | #IP0070 |
| *InVivo*MAb IgG2b isotype control | BioXCell | #BE0090 |
| *InVivo*MAb anti-mouse CD8a | BioXCell | #BE0061 |
| *InVivo*MAb polyclonal Syrian hamster IgG | BioXCell | #BE0087 |
| *InVivo*MAb anti-mouse PD-1 (CD279a) | BioXCell | #BP0033-2 |
|  | | |
| ***Flow Cytometry*** |  |  |
| Rabbit polyclonal, CRT (calreticulin) | Abcam | #ab2907 |
| True Stain FcX Plus anti-mouse CD16/32 | Biolegend | #156604 |
| Brilliant Violet 711 anti-mouse CD45 (Clone 30-F11) | Biolegend | #103147 |
| APC/Cyanine7 anti-mouse CD3 | Biolegend | #100722 |
| PE anti-mouse CD4 | Biolegend | #100408 |
| PE/ Cyanine7 anti-mouse CD8a | Biolegend | #100722 |
| Brilliant Violet 421 anti-mouse CD11c (Clone N418) | Biolegend | #117343 |
| APC/Cyanine7 anti-mouse CD25 | Biolegend | #102026 |
| FITC anti-mouse FoxP3 | ThermoFisher | #11-5773-82 |
| APC anti-mouse CD86 (Clone GL-1) | Biolegend | #105011 |
| Aombie Aqua dye | Biolegend | #NC0498216 |
| APC anti-human CD14 | Biolegend | #367117 |
| FITC anti-human CD86 | Biolegend | #374203 |
| PE anti-human HLA-DR | Biolegend | #361605 |
| APC anti-human CD83 | Biolegend | #305325 |
|  | | |
| ***Immunostaining*** |  |  |
| Goat anti-Mouse secondary antibodies, AlexaFluor 488 | ThermoFisher | #A-11059 |
| Goat anti-Rabbit secondary antibodies, AlexaFluor 594 | ThermoFisher | #A-11012 |
| Goat anti-Mouse secondary antibodies, AlexaFluor 594 | ThermoFisher | #A-21125 |
| Rabbit polyclonal, CD3 | Novus Biologicals | #NB600-1441 |
| Rabbit monoclonal, CD8α | ThermoFisher | #14-0195-82 |
| Rabbit monoclonal, CD11c | Cell Signaling | #97585 |
| Biotinylated goat anti-rat | ThermoFisher | #31830 |
| Goat anti-rabbit secondary antibodies, AlexaFluor 488 | ThermoFisher | #A-11008 |
| Rabbit IgG | Jackson ImmunoResearch | #011-000-002 |
| VectaShield + DAPI | Vector Laboratories | #H-1500 |
|  | | |
| **Assay Kits** |  |  |
| CellTiter-Glo Luminescent Cell Viability Assay | Promega | #G7572 |
| Chromatin Immunoprecipitation (ChIP) Assay Kit | Merck Millipore | #17-295 |
| DAB Kit | Vector Laboratories | #SK-4100 |
| CytoTox 96 Non-Radioactive Cytotoxicity Assay | Promega | #G1780 |
| CellTiter 96 AQueous One Solution Cell Proliferation Assay (MTS) | Promega | #G3582 |
| BD Annexin V: FITC Apoptosis Detection Kit II | BD Biosciences | #556570 |
| SensoLyte Homogeneous AMC Caspase-3/7 Assay Kit Fluorimetric-1 Kit | AnaSpec | AS-71118 |
| Quick-RNA Miniprep Kit | ZYMO Research | #R1055 |
| Luciferase Assay System | Promega | #E4550 |
| Venor^TM^ GeM Mycoplasma Detection Kit, PCR-based | Sigma-Aldrich | #MP0025 |
| Mitochondrial Fractionation Kit | Active Motif | #40015 |
| Rhod-2, AM, cell permeant | ThermoFisher | #R1244 |
| MitoTracker™ Green FM | ThermoFisher | # M7514 |
| EZview™ Red Protein G Affinity Gel | Sigma-Aldrich | #E3403 |
