# Supplementary material for: Depletion of oxysterol-binding proteins by OSW-1 triggers RIP1/RIP3-independent necroptosis and sensitization to cancer immunotherapy
Source: Cell Death Differ. 2025 May 6;32(11):2038–52. doi: 10.1038/s41418-025-01521-8 (PMC12572256; doi:10.1038/s41418-025-01521-8)
Supplement: Supplementary file 4 — Supplementary Table 2 [file 41418_2025_1521_MOESM4_ESM.docx]

| **Supplementary Table 2. PCR primer, siRNA, and sgRNA sequences** | |  |
| --- | --- | --- |
| **RT-PCR primers** | **Sequence** |  |
| Human *p53*-F | 5'-TGCGTGTTTGTGCCTGTCCT-3' |  |
| Human *p53*-R | 5'-GTGCTCGCTTAGTGCTCCCT-3' |  |
| Human *PUMA*-F | 5'-CGACCTCAACGCACAGTACGA-3' |  |
| Human *PUMA*-R | 5'-AGGCACCTAATTGGGCTCCAT-3' |  |
| Human *β-actin*-F | 5'-GACCTGACAGACTACCTCAT-3' |  |
| Human *β-actin*-R | 5'-AGACAGCACTGTGTTGGCTA-3' |  |
| Human *OSBP*-F | 5'-GATCCATCAGGAAAAGTCCAC-3' |  |
| Human *OSBP*-R | 5'-CAGTGCCACTTTCCCAAGCA-3' |  |
| Human *ORP4*-F | 5'-AAGCTCTGGATCGACCAGTCA-3' |  |
| Human *ORP4*-R | 5'-CTGGGACTGCTATGCATGACC-3' |  |
| Human *Mdm2*-F | 5'-GCAGTGAATCTACAGGGACGC-3' |  |
| Human *Mdm2*-R | 5'-ATCCTGATCCAACCAATCACC-3' |  |
| Human *CaMKII-α*-F | 5'-TCCGGAGGGAAGAGTGGGGGAAAC-3' |  |
| Human *CaMKII-α*-R | 5'-GCTGTCATGCCAGGGTCGCACAT-3' |  |
| Human *CaMKII-β*-F | 5'-AATTTCTCAGTGGGCAGACAGAC-3' |  |
| Human *CaMKII-β*-R | 5'-CTCCTTAATCCCGTCCACTG-3' |  |
| Human *CaMKIIδ*-F | 5'-GGATCTGTCAACGTTCTACT-3' |  |
| Human *CaMKIIδ*-R | 5'-TGTGGATTACAGTAGTTTGG-3' |  |
| Human *CaMKII-γ*-F | 5'-TCCTGTATATCCTCCTGGT-3' |  |
| Human *CaMKII-γ*-R | 5'-CATCTGGTTGATCAAGTTC-3' |  |
| Human *IFNB*-F | 5'-TTGAATGGGAGGCTTGAATA-3' |  |
| Human *IFNB*-R | 5'-CTATGGTCCAGGCACAGTGA-3' |  |
| Human *TNFA*-F | 5'-CCTCTCTCTAATCAGCCCTCTG-3' |  |
| Human *TNFA*-R | 5'-GAGGACCTGGGAGTAGATGAG-3' |  |
| Mouse *IFNB*-F | 5'-AAGAGTTACACTGCCTTTGCCATC-3' |  |
| Mouse *IFNB*-R | 5'-CACTGTCTGCTGGTGGAGTTCATC-3' |  |
| Mouse *TNFA*-F | 5'-TTCTGTCTACTGAACTTCGGGGTGATCGGTCC-3' |  |
| Mouse *TNFA*-R | 5'-GTATGAGATAGCAAATCGGCTGACGGTGTGGG-3' |  |
| **siRNA** | **Sequence** |  |
| Control scrambled | 5'-AACGTACGCGGAATACTTCGA-3' |  |
| Human *MLKL* | 5’- CAAACTTCCTGGTAACTCA -3’ |  |
| Human *RIP1-1* | 5’-CAGCTTGATTTACGTCAGCCA-3’ |  |
| Human *RIP1-2* | 5’-CCGACATTTCCTGGCATTGAA-3’ |  |
| Human *RIP3* | 5’-CCAGAGACCTCAACTTTCA-3’ |  |
| Human *CaMKIIδ* | 5’-AAGACATAGTGGCAAGAGAAT-3’ |  |
| Human *OSBP* | 5’-TACTGGGAGTGTAAAGAAA-3’ |  |
| Human *ORP4* | 5'-GCAAUGGUUUGCUCUCUUA-3' |  |
| Human *PUMA* | 5’-CCAGAGACCTCAACTTTCA-3’ |  |
| Human *p53* | 5’-GTGAGCGCTTCGAGATGTT-3’ |  |
| Human *β-TRCP1* and *2* | 5’-AAGUGGAAUUUGUGGAACAUC-3’ |  |
| **sgRNA** | **Sequence** |  |
| Human *MLKL* | 5’-GCTTTCCAGATGCTAAGAAG-3’ |  |
| Human *CaMKIIδ* | 5’-ATGACAATGGAGAATTGAGG-3’ |  |
| Mouse *MLKL* | 5’-GACTTCATCAAAACGGCCCA-3’ |  |
