# Supplementary material for: Depletion of oxysterol-binding proteins by OSW-1 triggers RIP1/RIP3-independent necroptosis and sensitization to cancer immunotherapy
Source: Cell Death Differ. 2025 May 6;32(11):2038–52. doi: 10.1038/s41418-025-01521-8 (PMC12572256; doi:10.1038/s41418-025-01521-8)

Original Western blot pictures

Lu *et al* “Depletion of oxysterol-binding proteins by OSW-1 triggers RIP1/RIP3-independent necroptosis and sensitization to cancer immunotherapy”

Fig. 1D

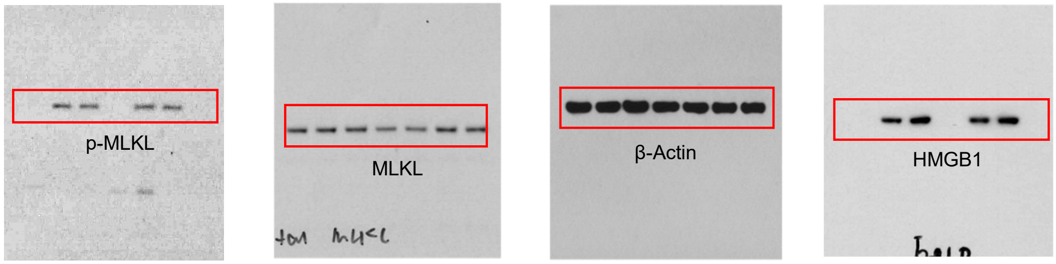

Fig. 1F

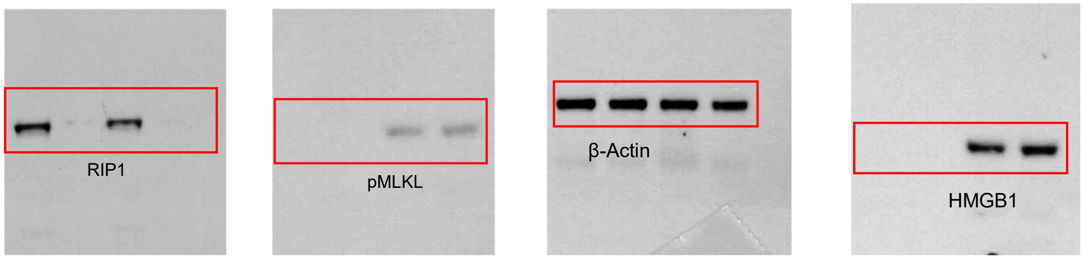

Fig. 1G

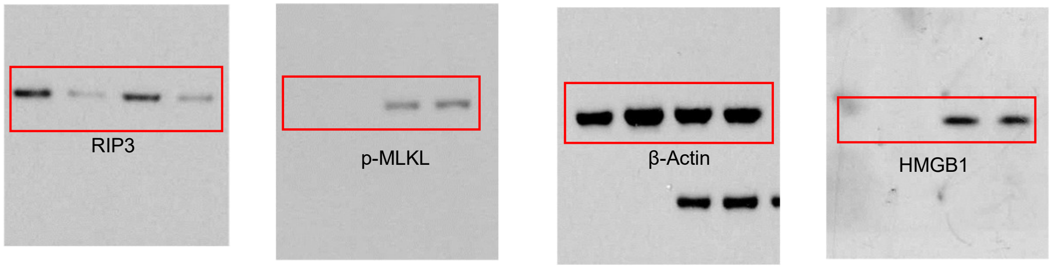

Fig. 1H

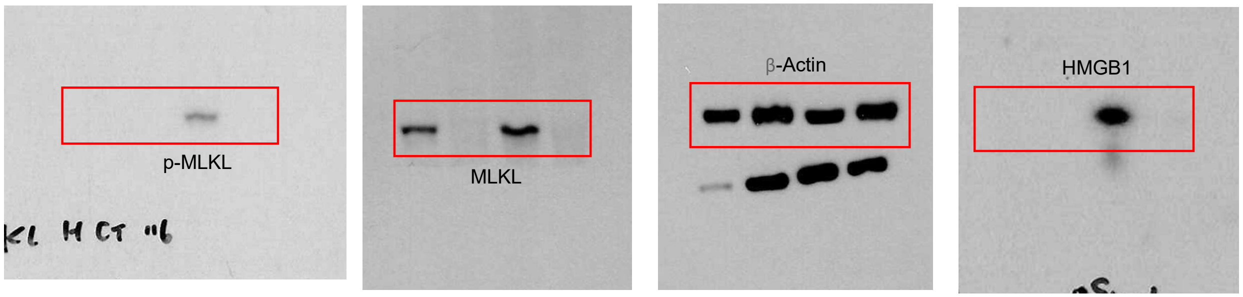

Fig. 1I

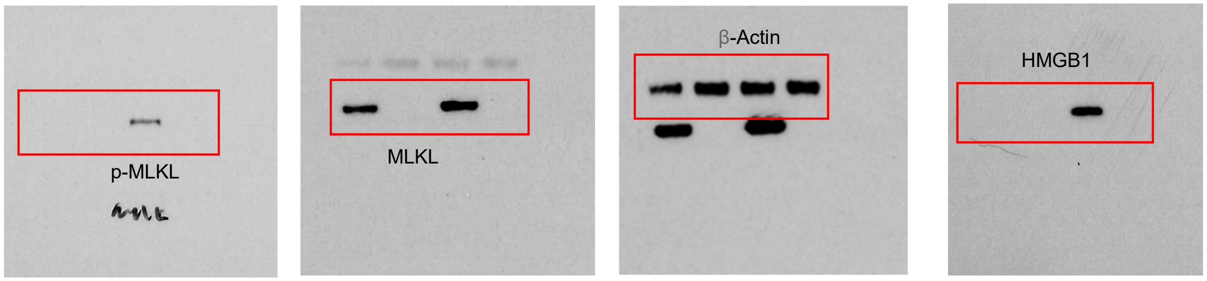

Fig. 2B

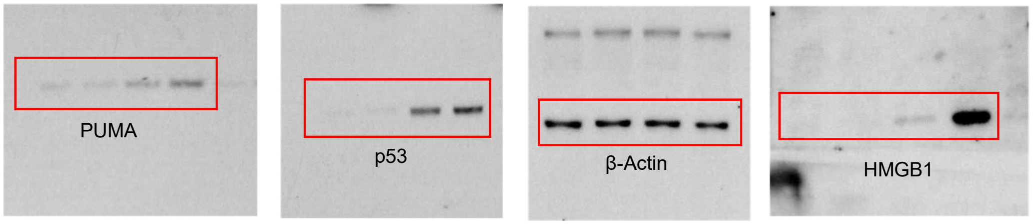

Fig. 2C

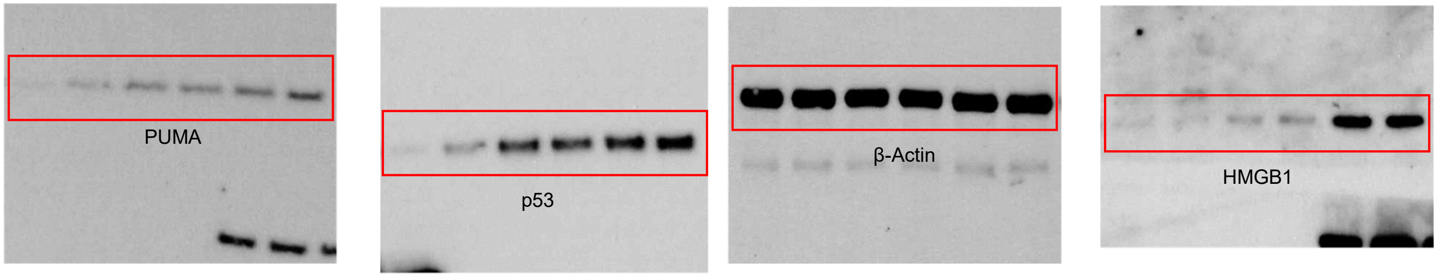

Fig. 2D

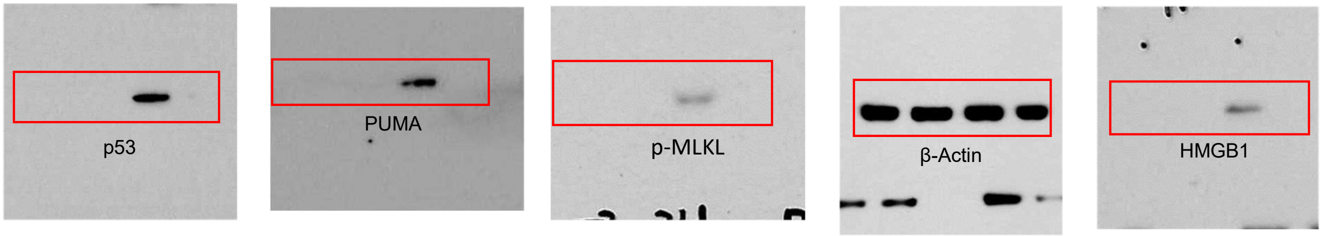

Fig. 2E

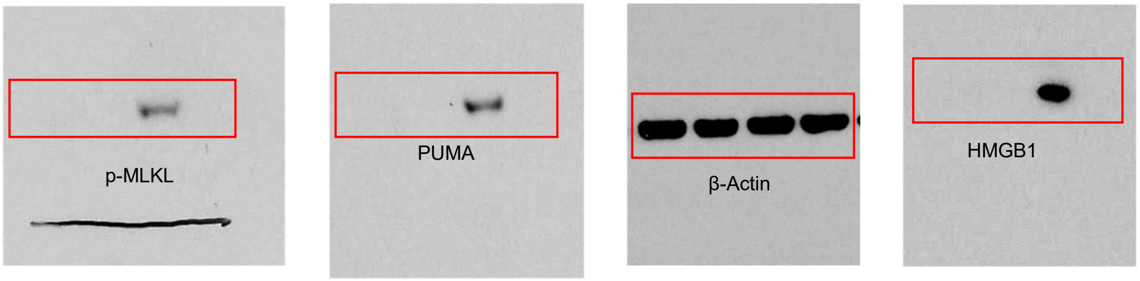

Fig. 2L

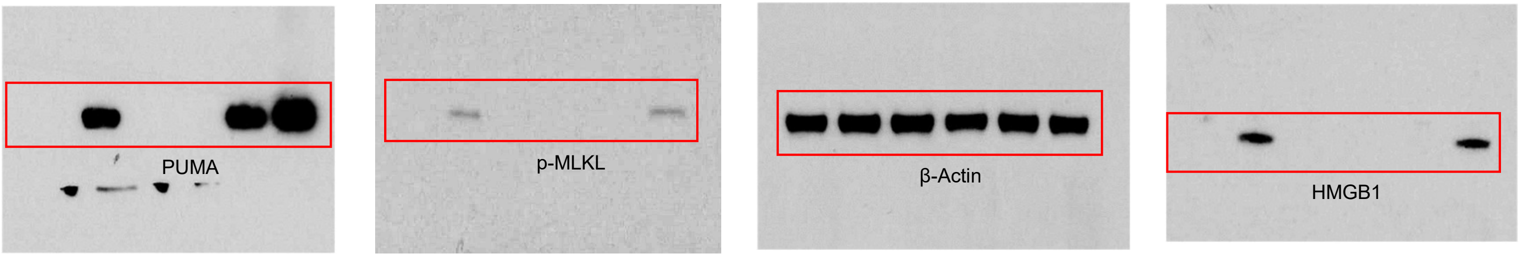

Fig. 3A

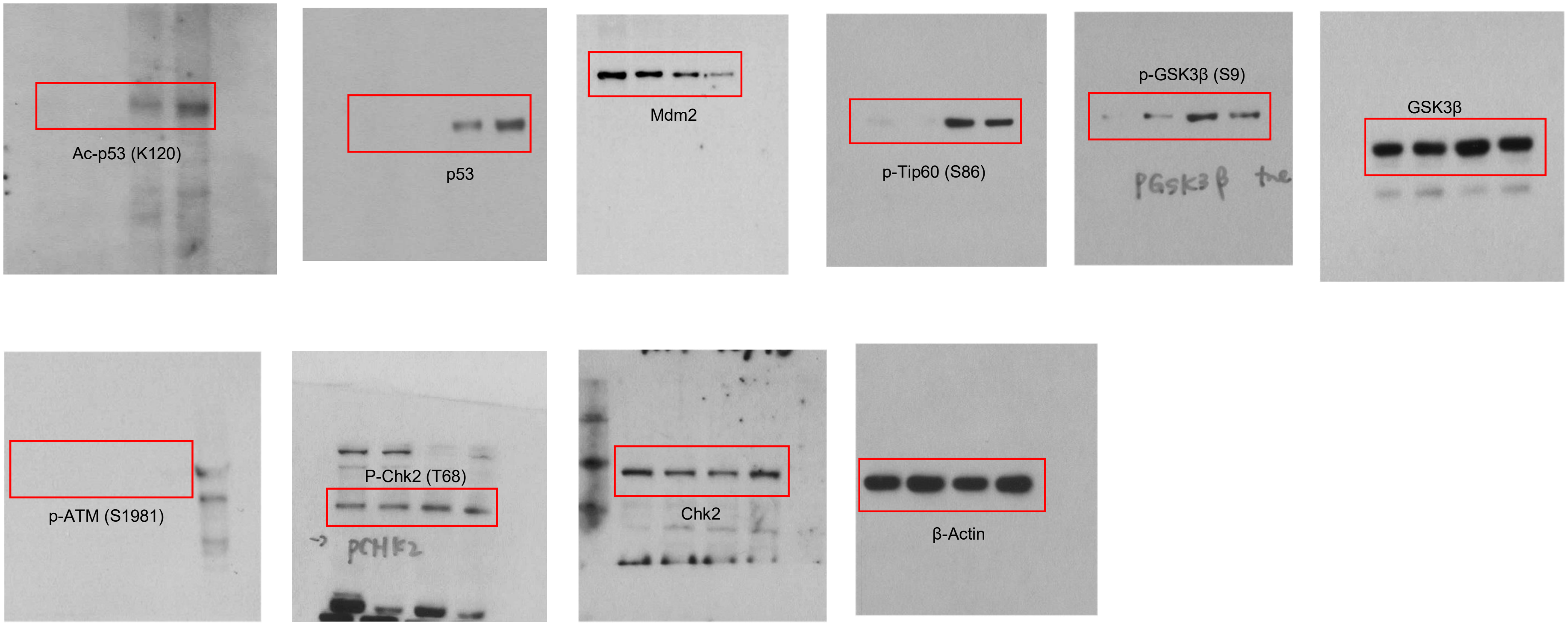

Ac-p53 (K120)
p53
Mdm2
p-Tip60 (S86)
p-GSK3β (S9)
GSK3β
p-ATM (S1981)
P-Chk2 (T68)
Chk2
β-Actin

Fig. 3B

Top panel

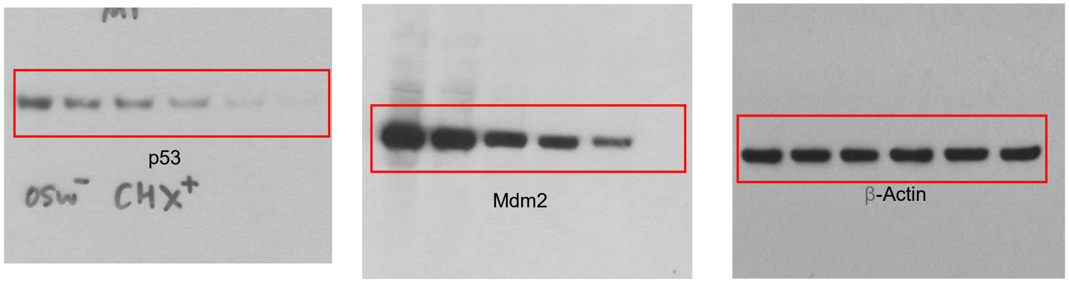

Bottom panel

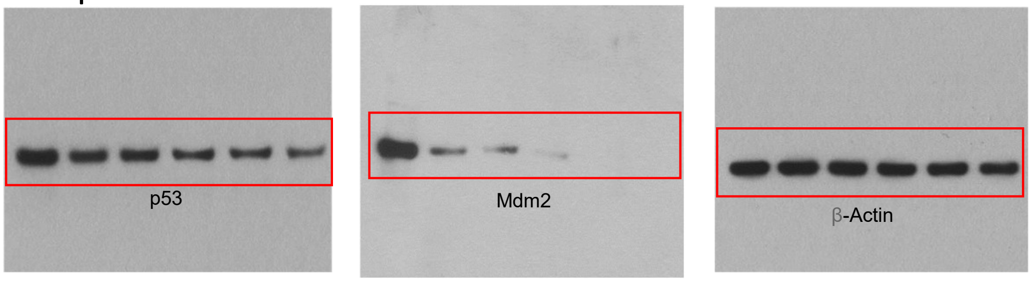

Fig. 3D

Top panel

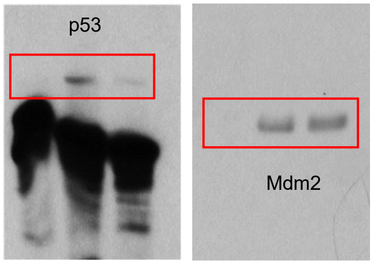

Bottom panel

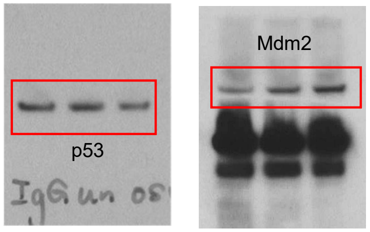

Fig. 3E

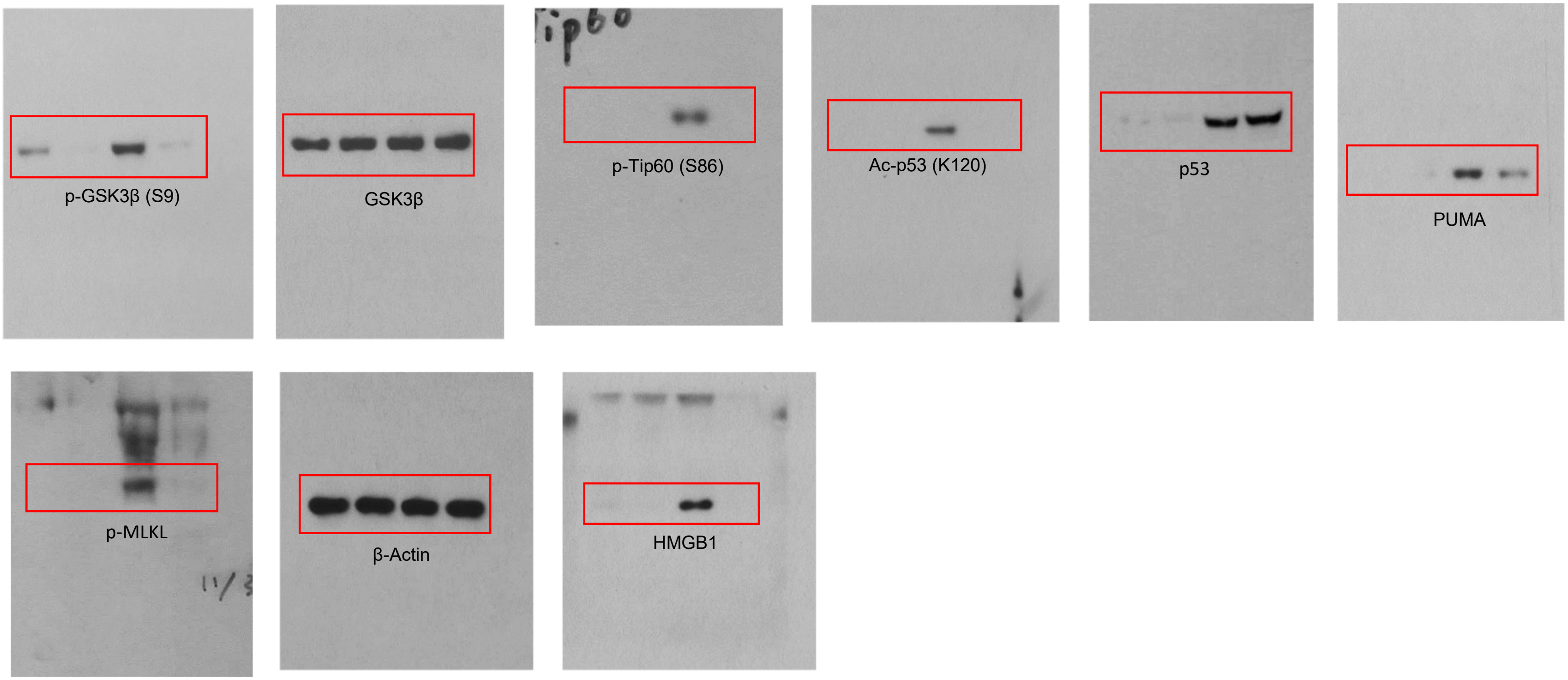
p-GSK3β (S9)
GSK3β
p-Tip60 (S86)
Ac-p53 (K120)
p53
PUMA
p-MLKL
β-Actin
HMGB1

Fig. 3G

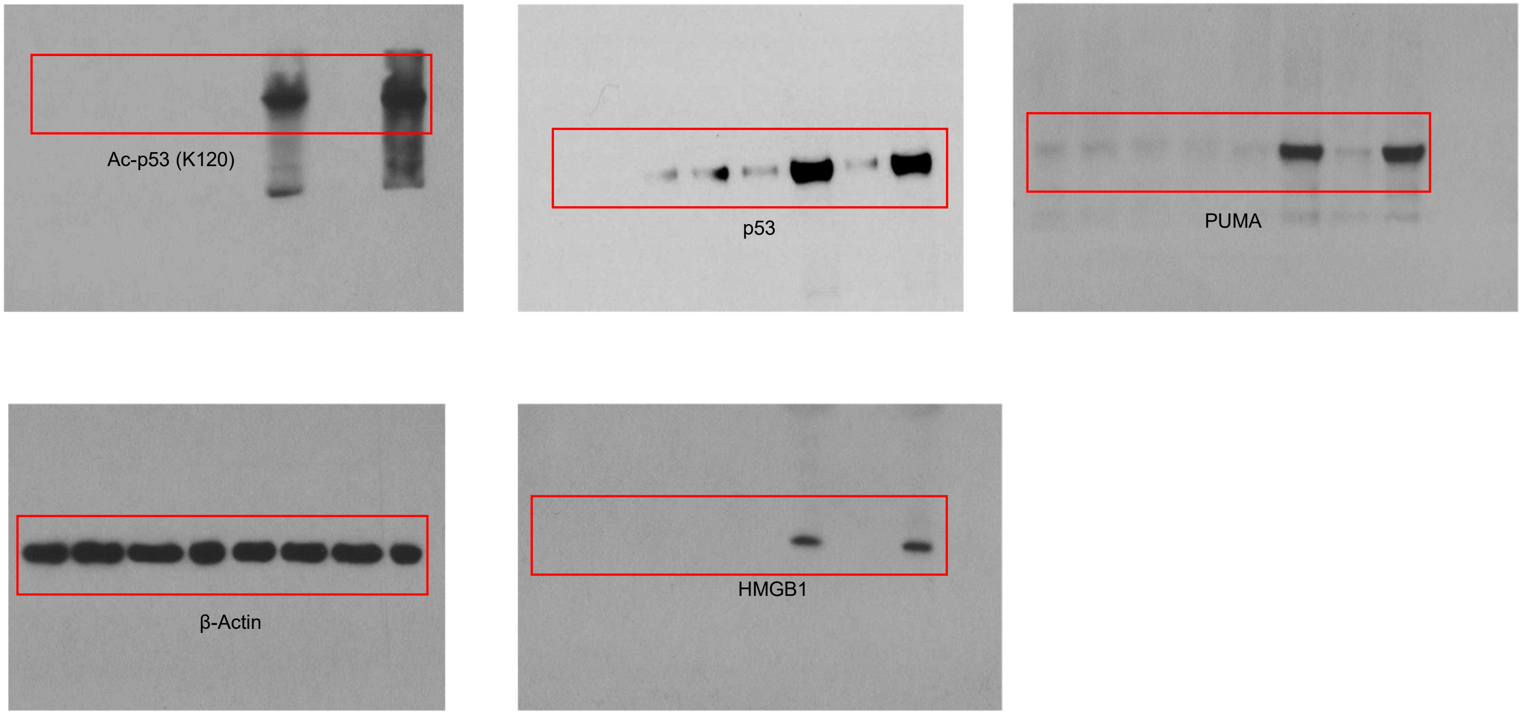

Fig. 4A

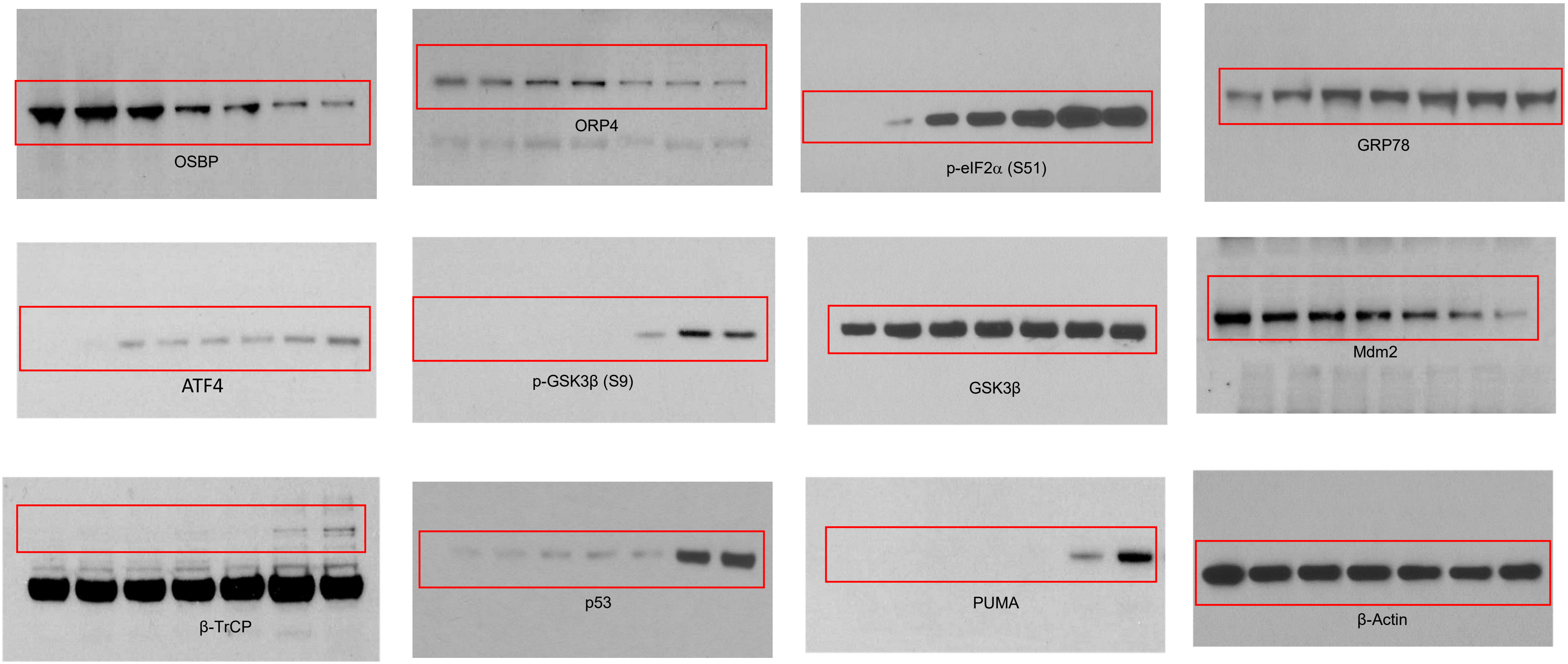
OSBP
ORP4
p-eIF2α (S51)
GRP78
ATF4
p-GSK3β (S9)
GSK3β
Mdm2
β-TrCP
p53
PUMA
β-Actin

Fig. 4C

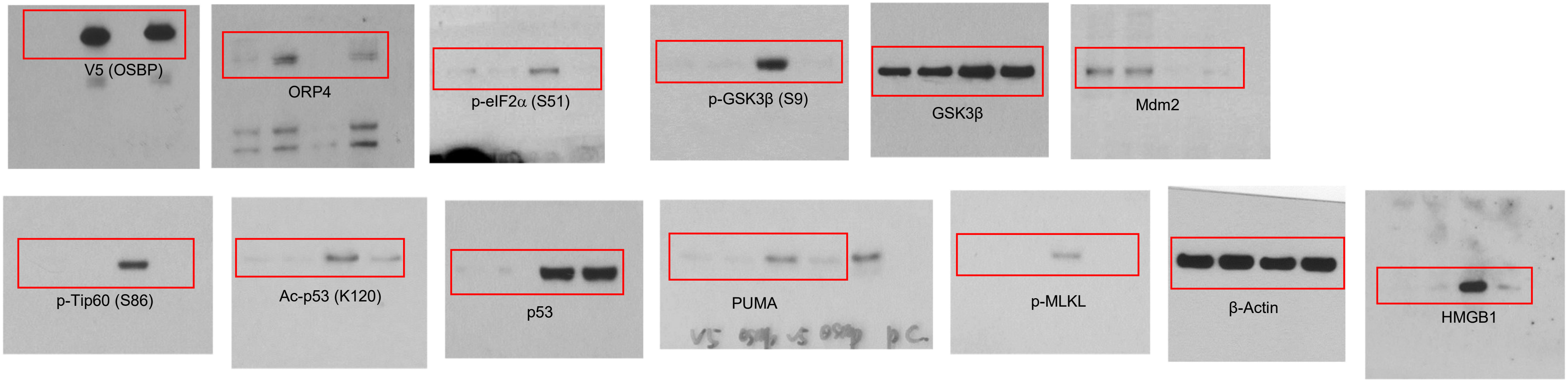
V5 (OSBP)
ORP4
p-eIF2α (S51)
p-GSK3β (S9)
GSK3β
Mdm2
p-Tip60 (S86)
Ac-p53 (K120)
p53
PUMA
p-MLKL
β-Actin
HMGB1

Fig. 4D

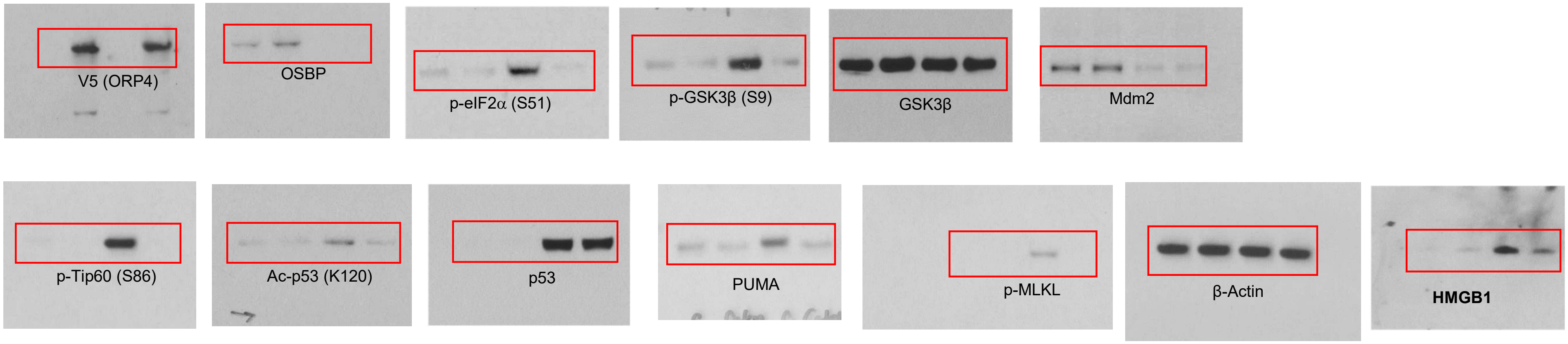
V5 (ORP4)
OSBP
p-eIF2α (S51)
p-GSK3β (S9)
GSK3β
Mdm2
p-Tip60 (S86)
Ac-p53 (K120)
p53
PUMA
p-MLKL
β-Actin
HMGB1

Fig. 4E

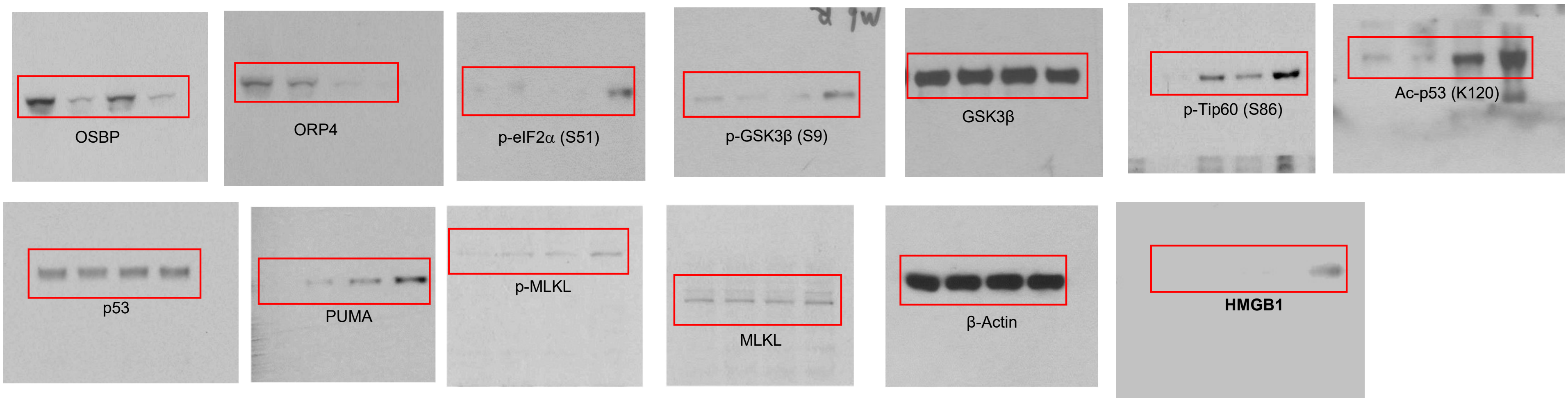
OSBP
ORP4
p-eIF2α (S51)
p-GSK3β (S9)
GSK3β
p-Tip60 (S86)
Ac-p53 (K120)
p53
PUMA
p-MLKL
MLKL
β-Actin
HMGB1

Fig. 4H

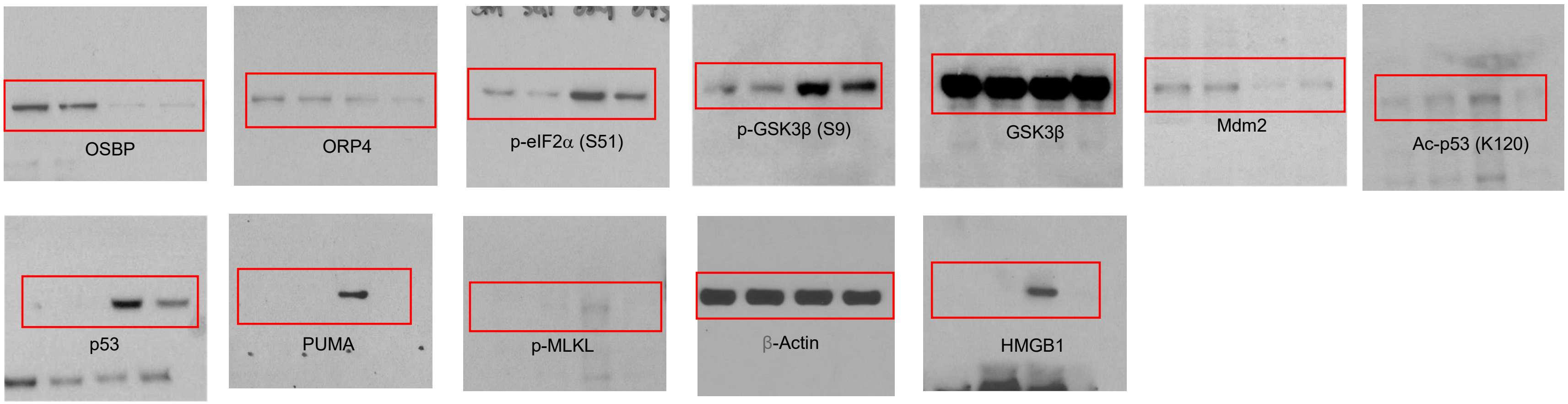
OSBP
ORP4
p-eIF2α (S51)
p-GSK3β (S9)
GSK3β
Mdm2
Ac-p53 (K120)
p53
PUMA
p-MLKL
β-Actin
HMGB1

Fig. 5A

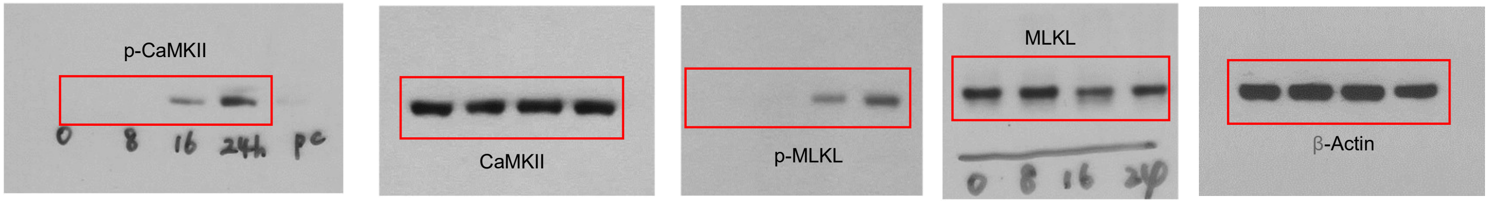

Fig. 5B

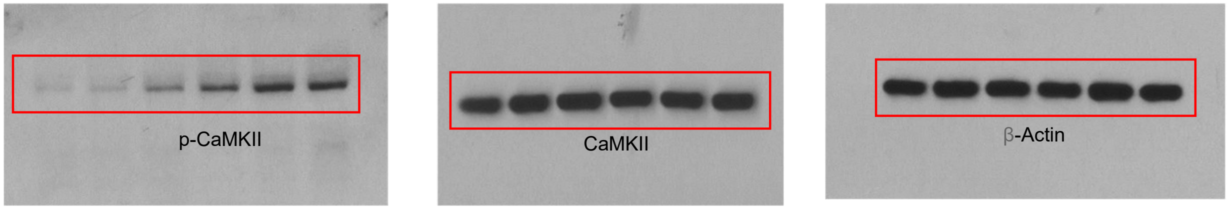

Fig. 5C

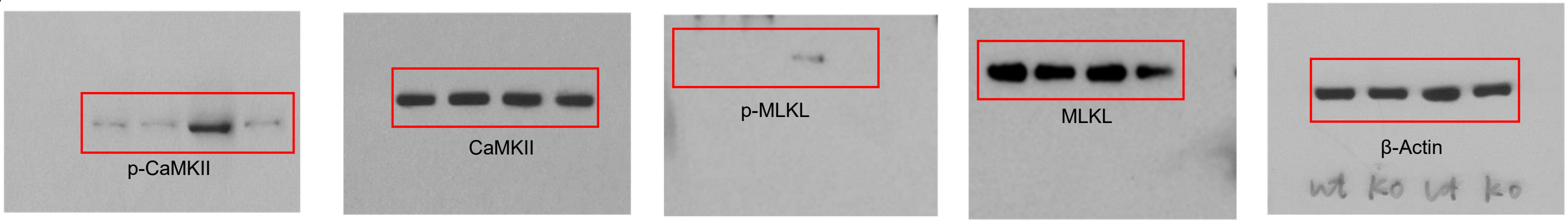

Fig. 5D

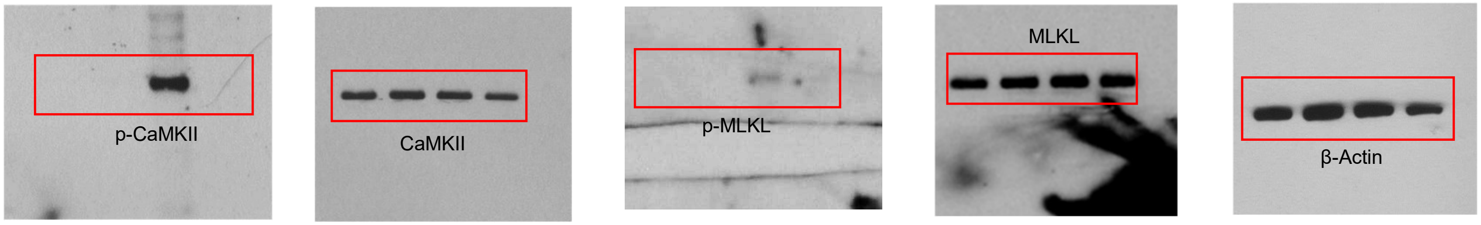

Fig. 5I

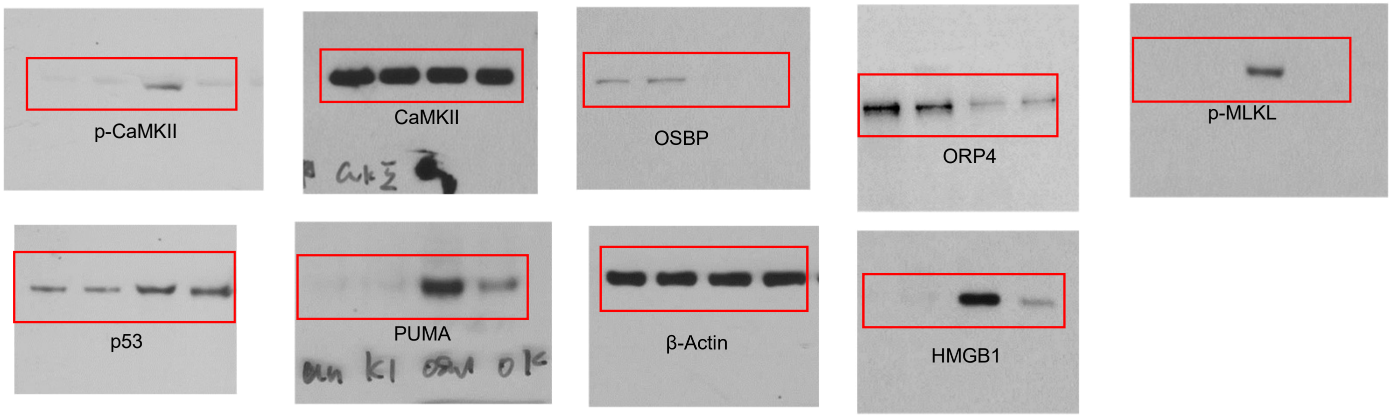

Fig. 5J

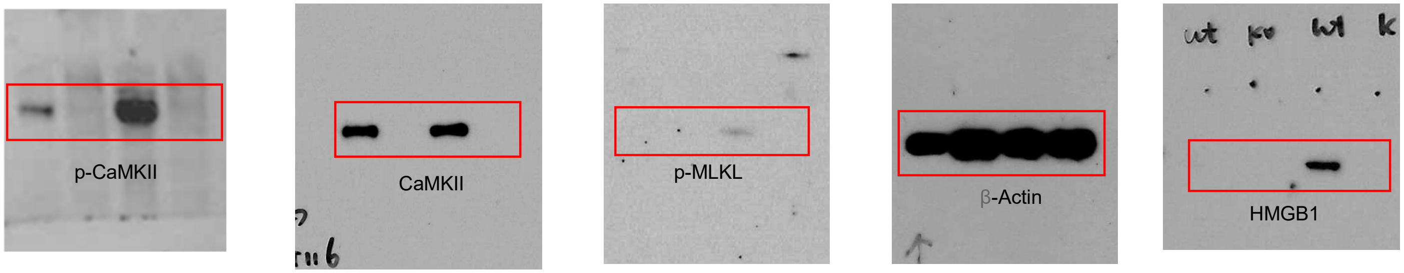

Fig. 5K

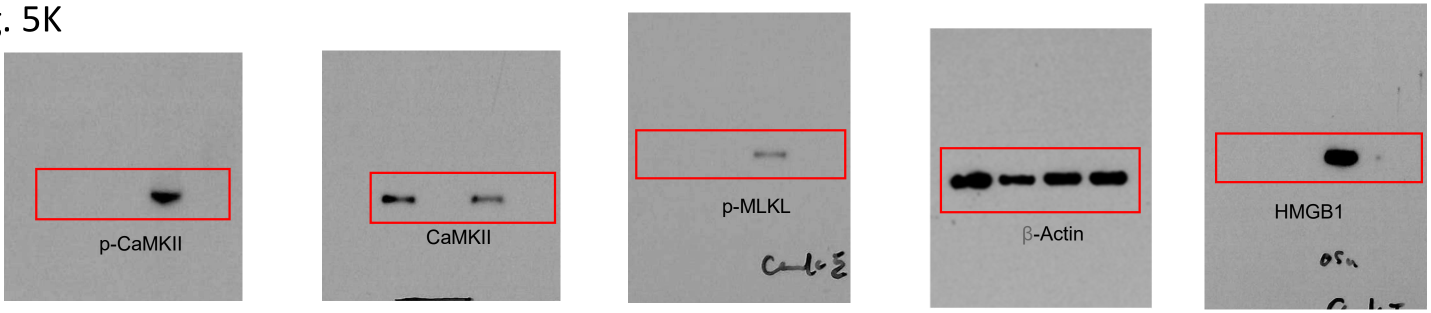

Fig. 5L

Left panel

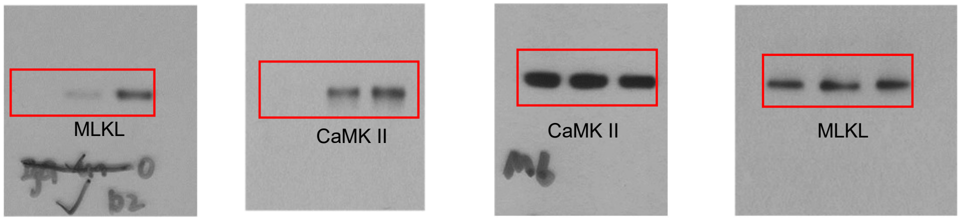

Right panel

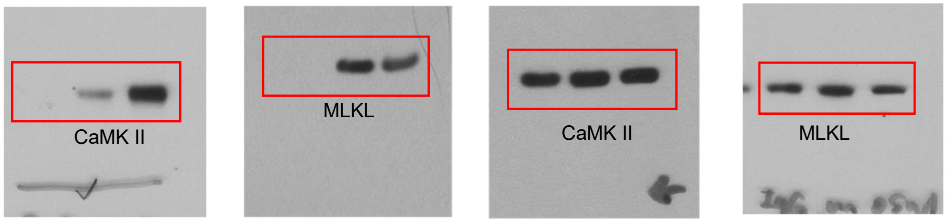

Fig. 6D

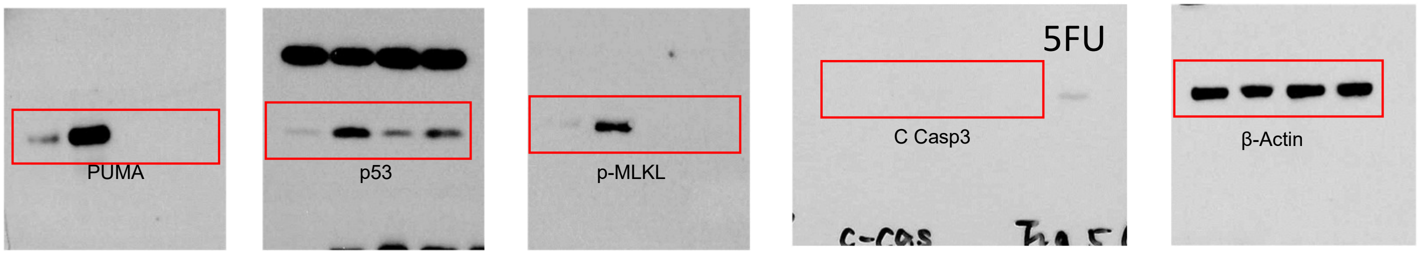

Fig. 6H

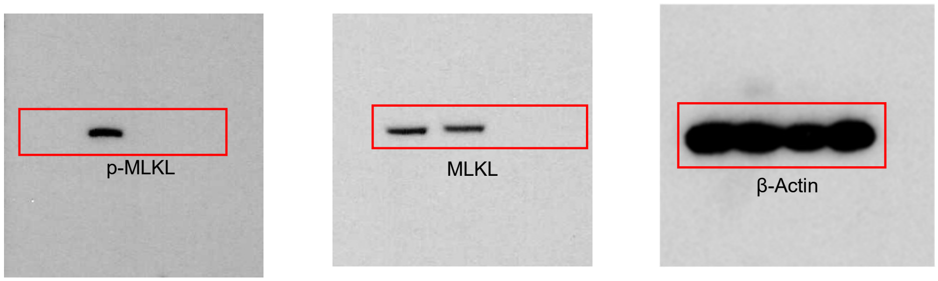

Supplementary Fig. 1B

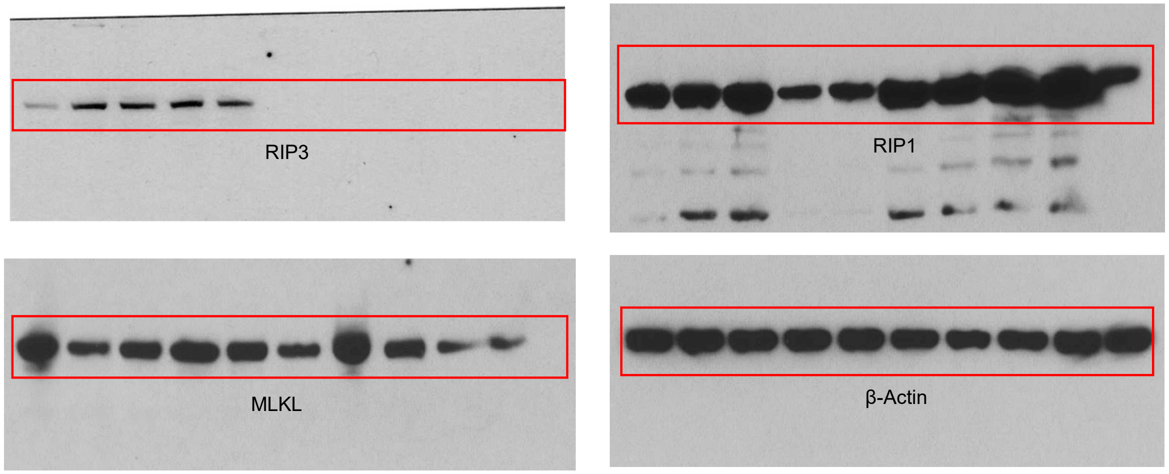

Supplementary Fig. 1C

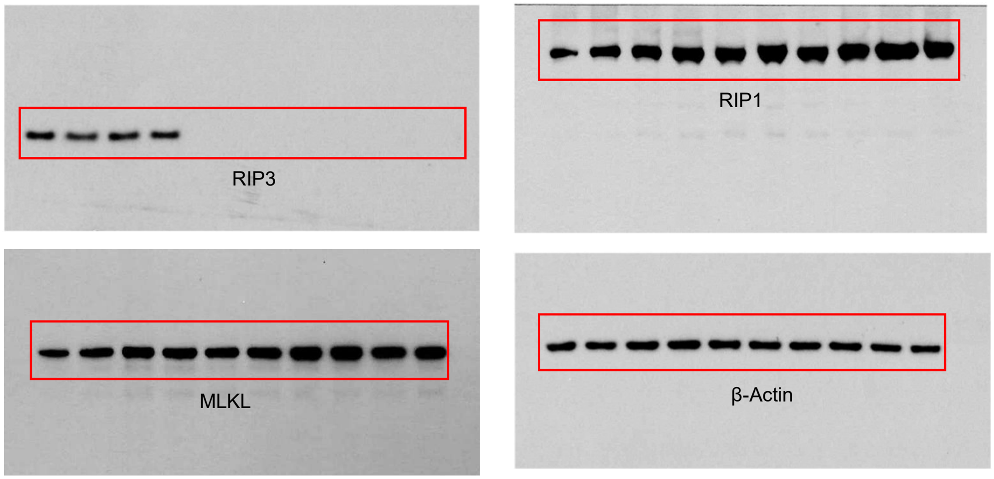

Supplementary Fig. 1E

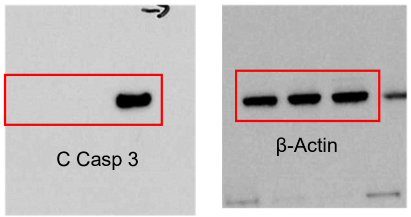

Supplementary Fig. 2B

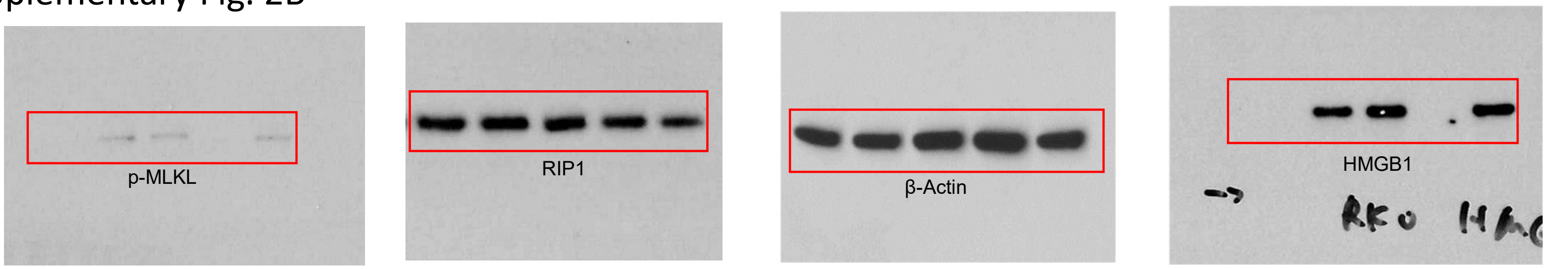

Supplementary Fig. 2C

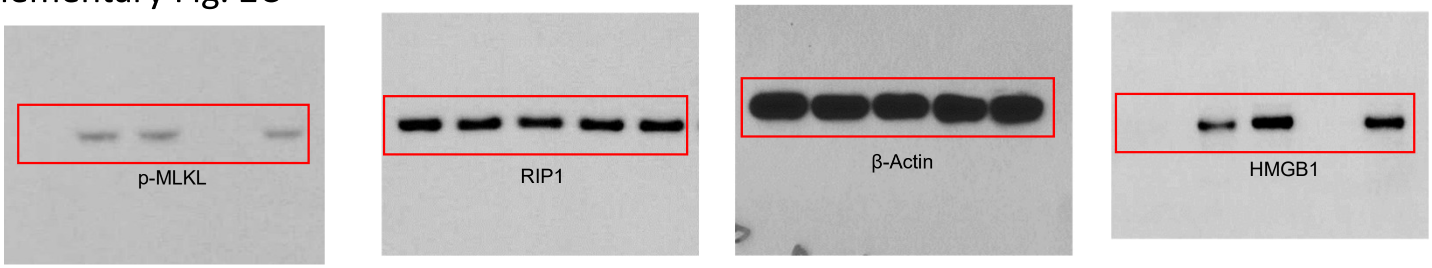

Supplementary Fig. 2D

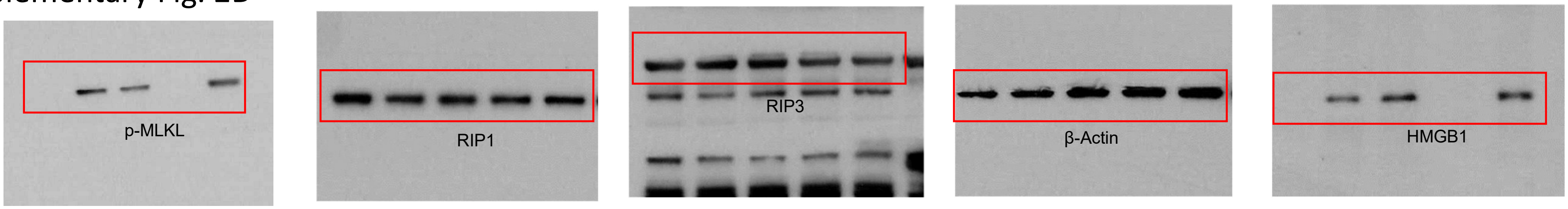

Supplementary Fig. 2E

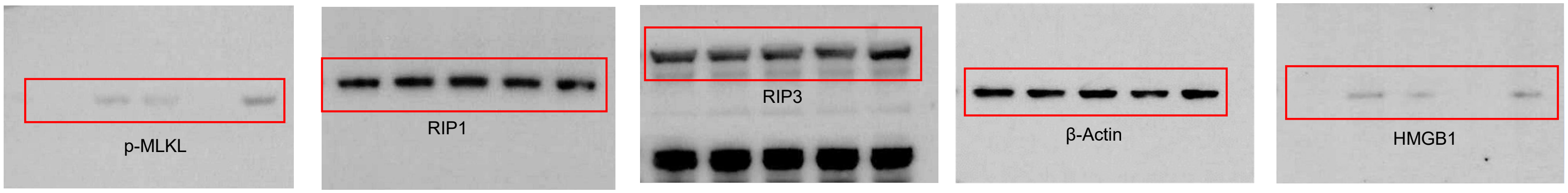

Supplementary Fig. 2F

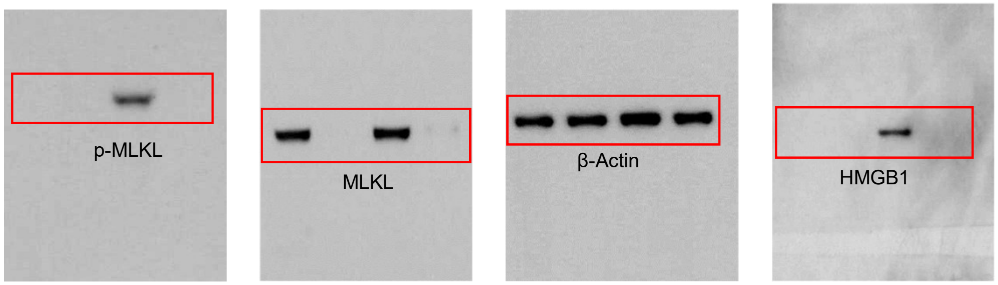

Supplementary Fig. 2G

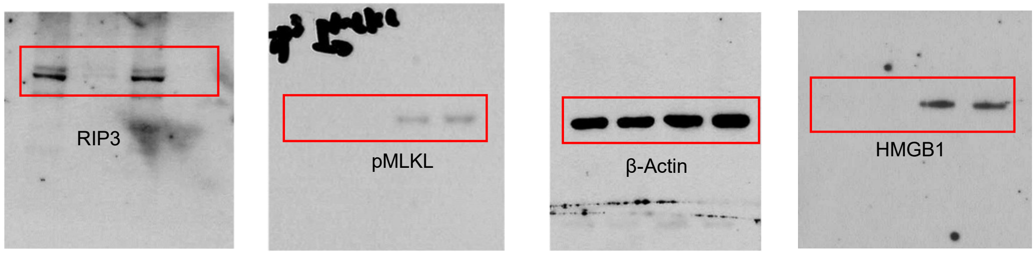

Supplementary Fig. 3A

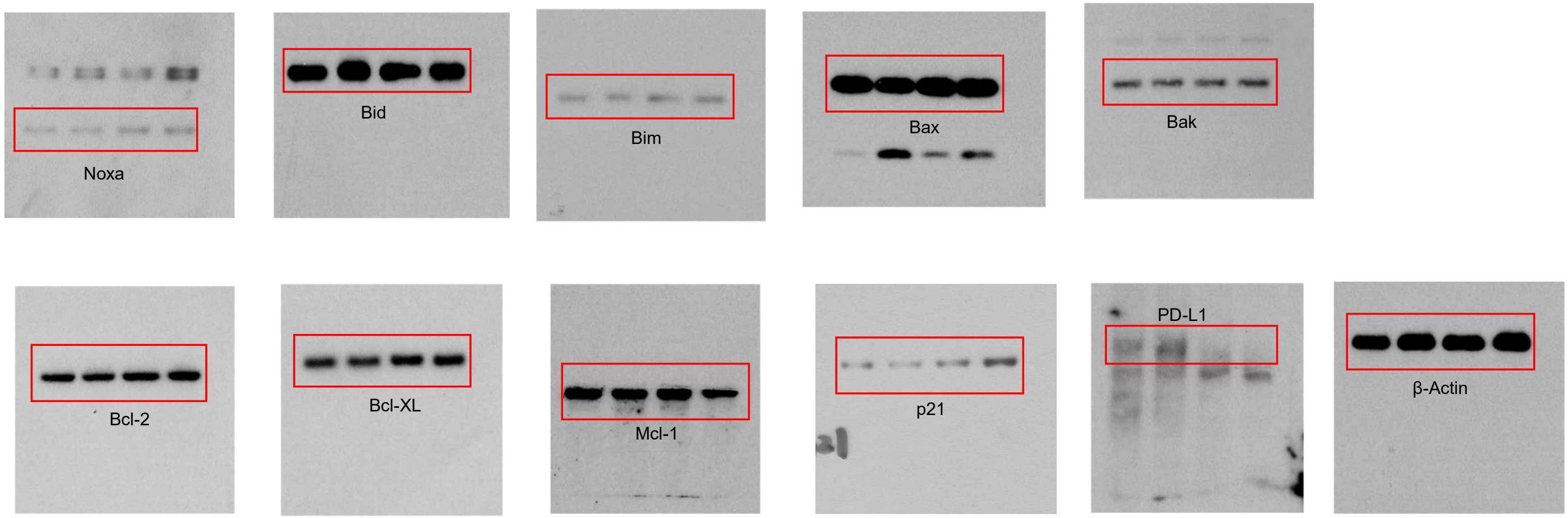

Supplementary Fig. 3B

Top panel

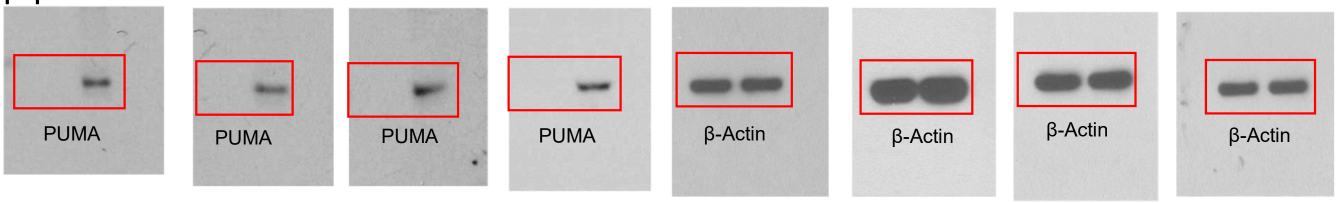

Bottom panel

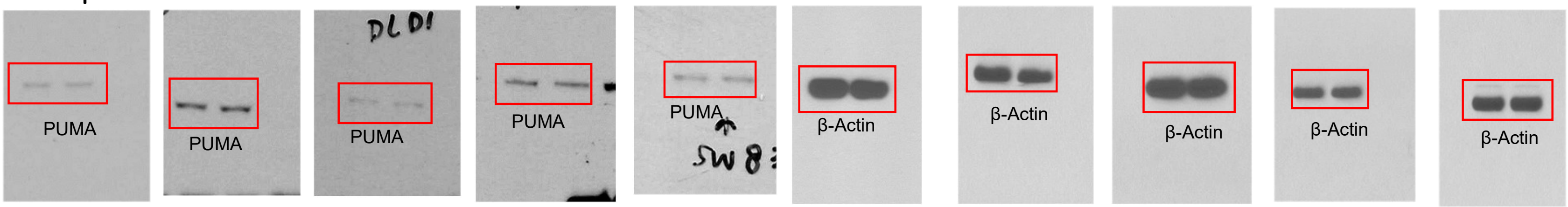

Supplementary Fig. 3E

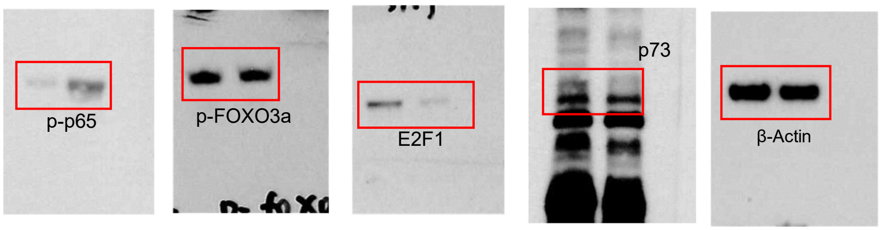

Supplementary Fig. 3F

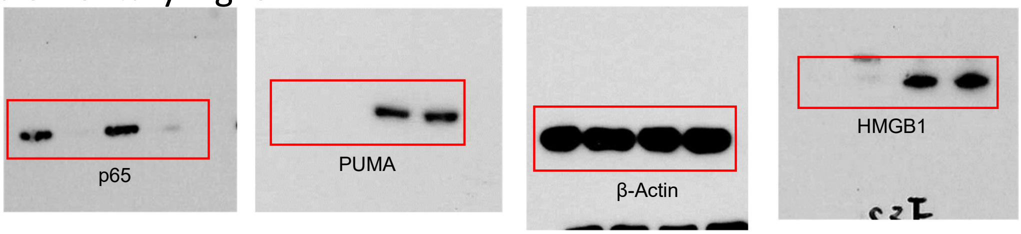

Supplementary Fig. 4B

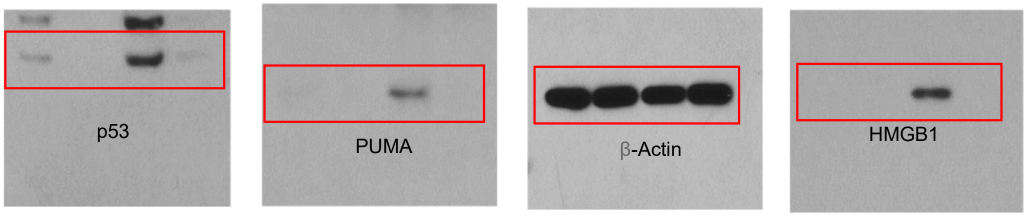

Supplementary Fig. 4C

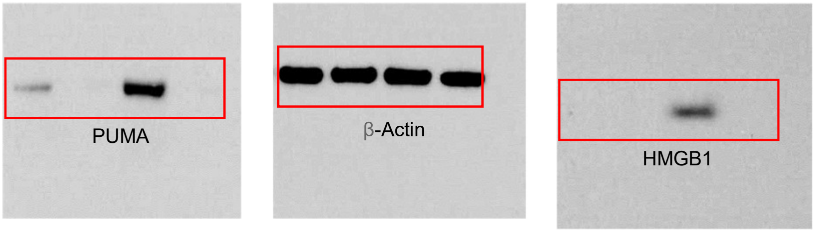

Supplementary Fig. 4D

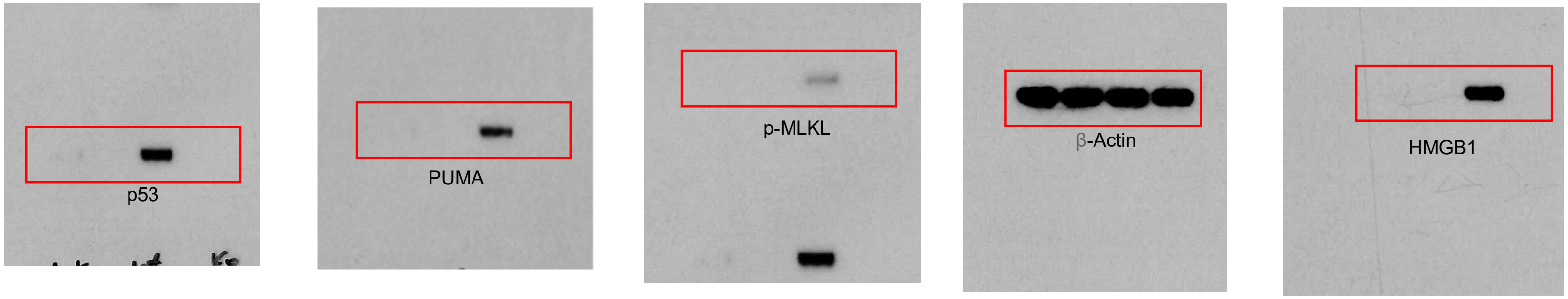

Supplementary Fig. 4E

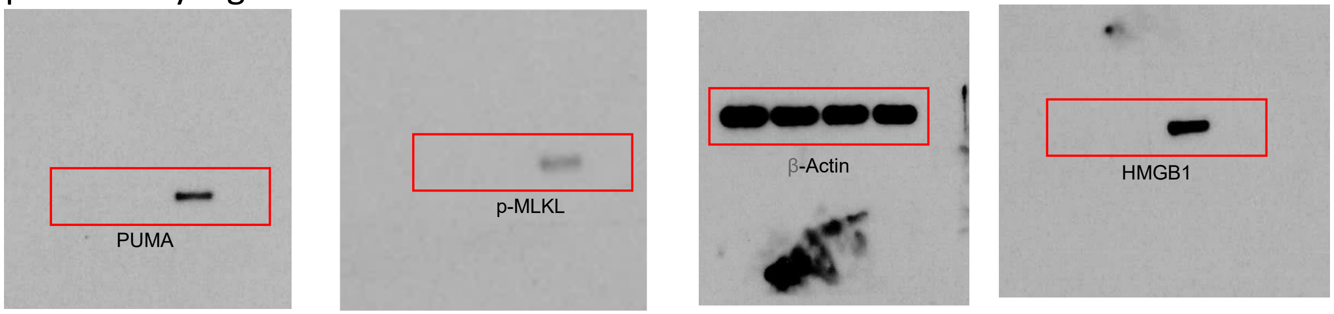

Supplementary Fig. 4F

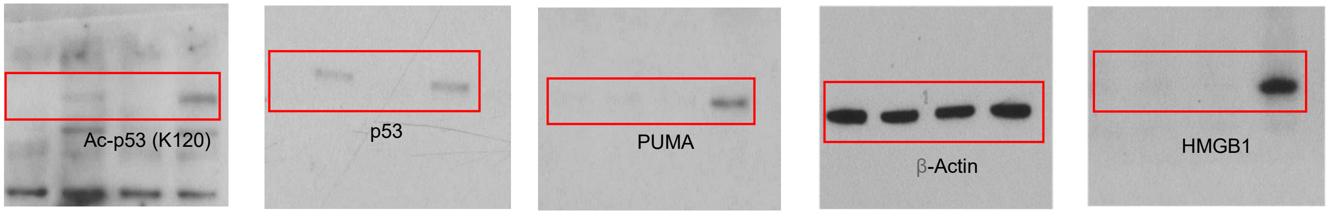

Supplementary Fig. 4H

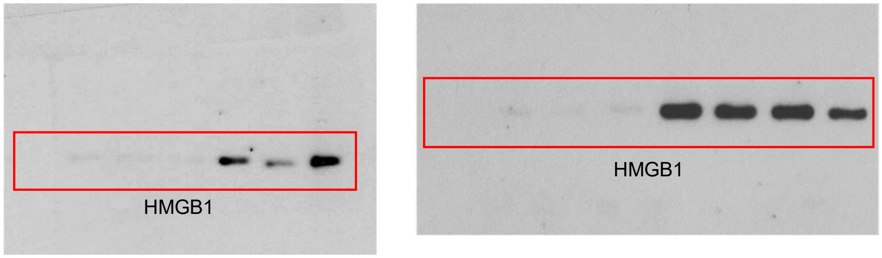

Supplementary Fig. 4I

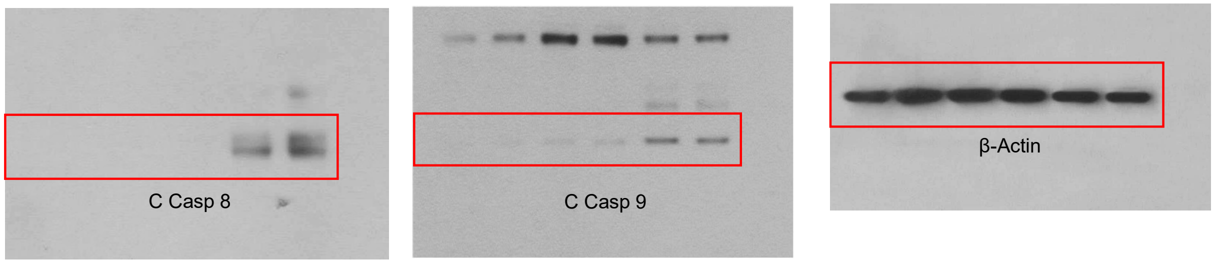

Supplementary Fig. 4J

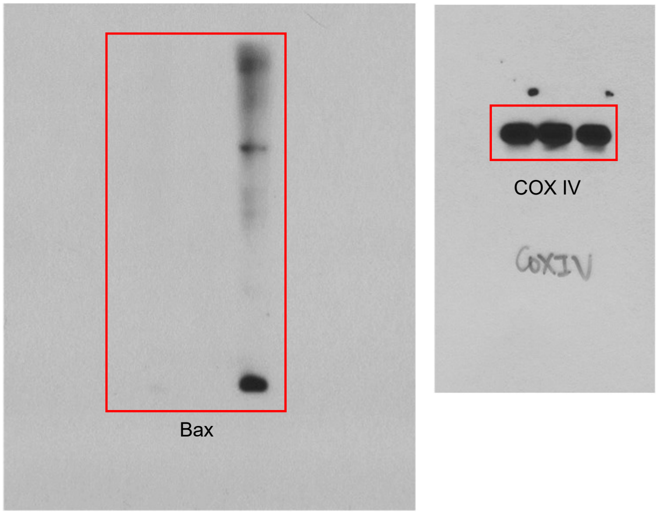

Supplementary Fig. 4K

Top panel

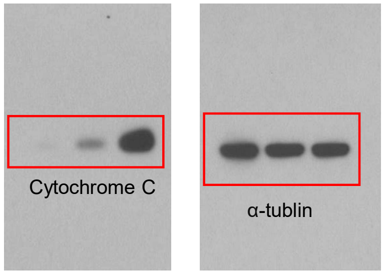

Bottom panel

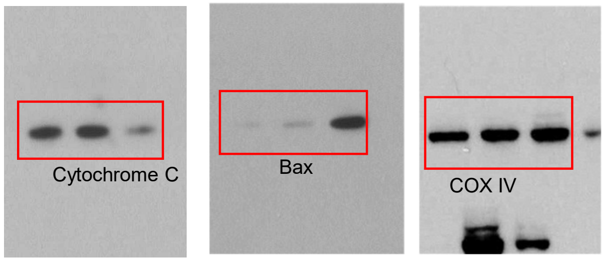

Supplementary Fig. 5A

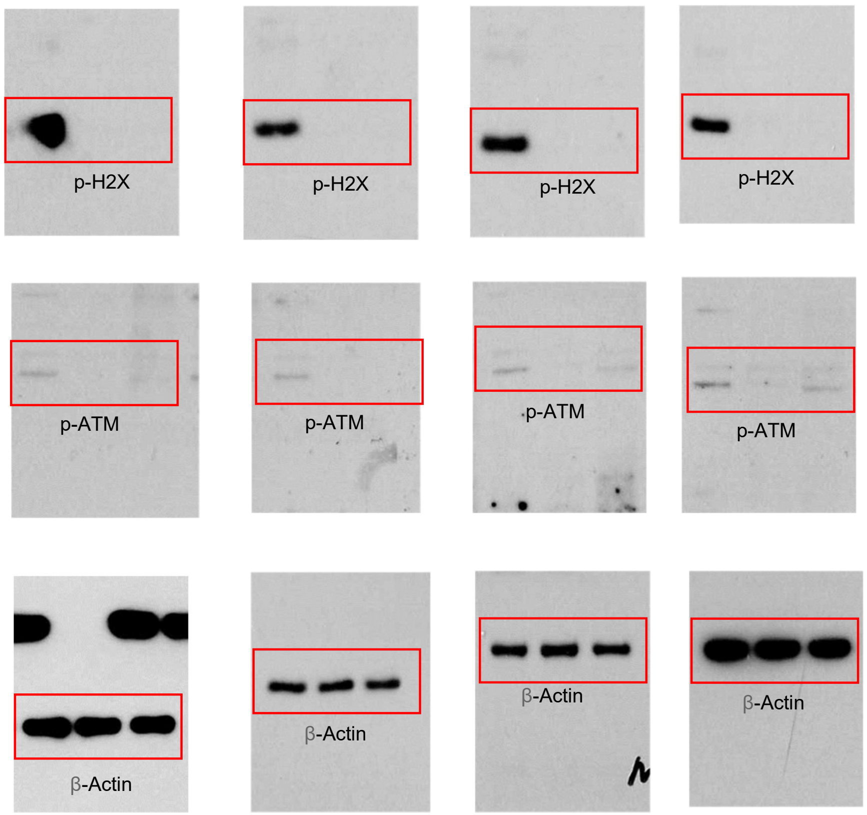

Supplementary Fig. 5D

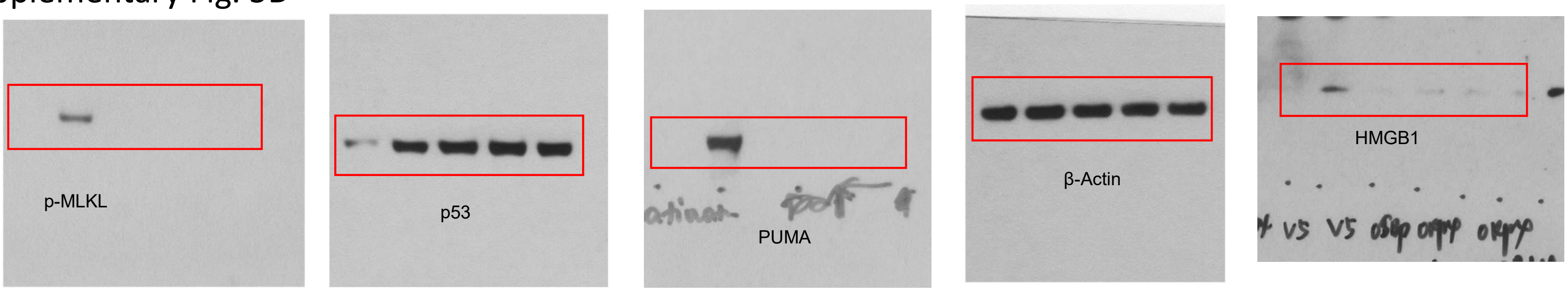

Supplementary Fig. 5E

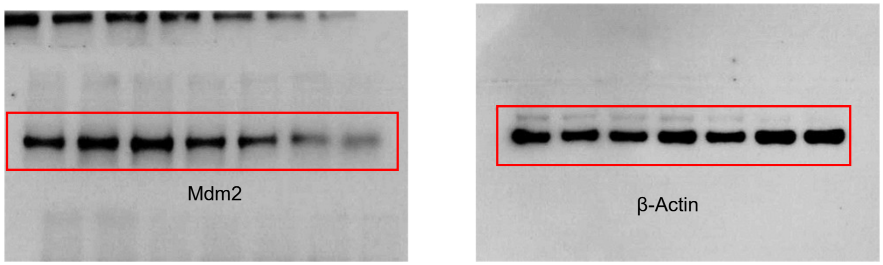

Supplementary Fig. 5F

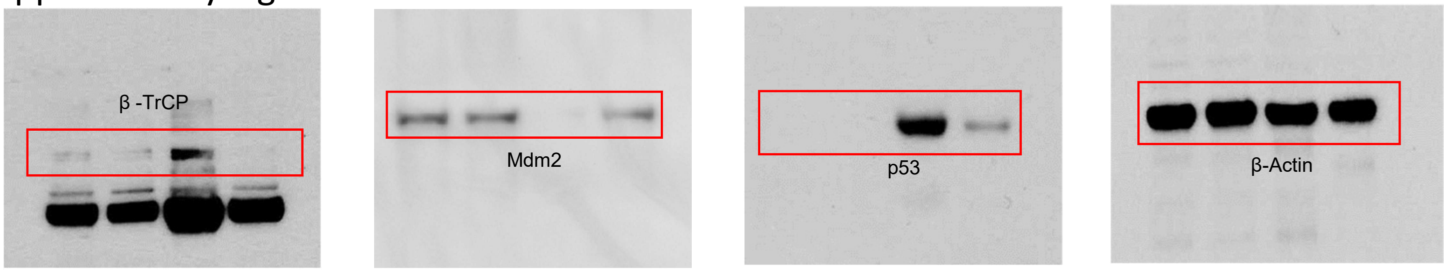

Supplementary Fig. 6C

Top panel

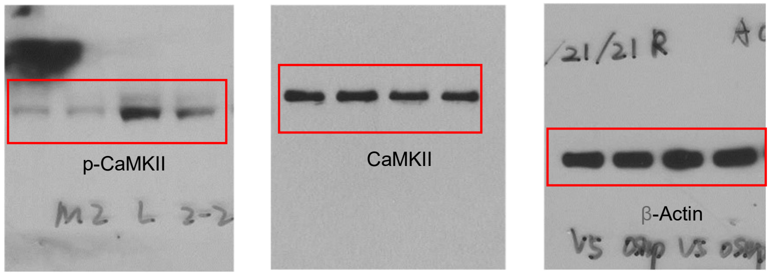

Bottom panel

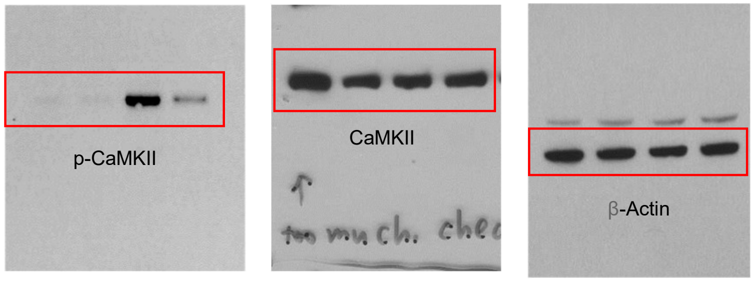

Supplementary Fig. 6K

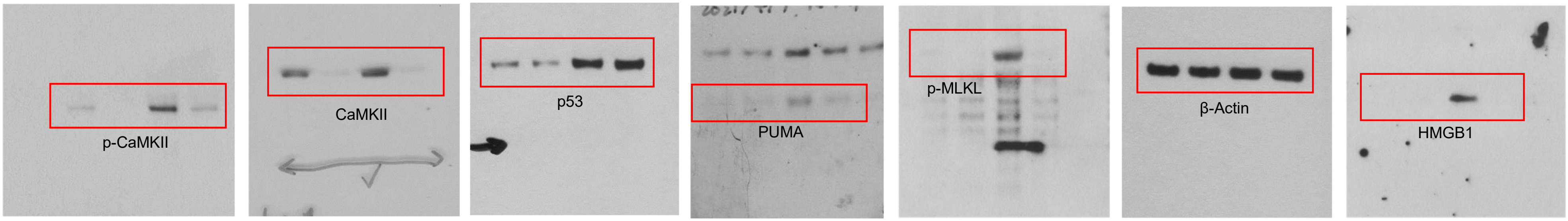

Supplement: Supplementary file 5 — Original Western blot pictures [file 41418_2025_1521_MOESM5_ESM.pdf]
